# Reconsidering Catechol Contributions in Quantitative ^31^P NMR Analyses of Lignins

**DOI:** 10.1021/acsomega.6c09845

**Published:** 2026-10-28

**Authors:** Nicolò Pajer, Paula A. Nousiainen, Monika Österberg

**Affiliations:** Department of Bioproducts and Biosystems, 174277 Aalto University, Vuorimiehentie 1, Espoo 02150, Finland

## Abstract

Quantitative ^31^P NMR spectroscopy is widely used to determine phenolic hydroxyl group contents in lignins, yet the contribution of catechol functionalities is generally ambiguous. Using a series of catechol-containing lignin model compounds, we demonstrate that phosphitylated catechol hydroxyl groups yield signals that overlap extensively with conventional guaiacyl and syringyl regions and cannot be reliably distinguished by quantitative ^31^P NMR spectroscopy. Similar results were obtained by aromatic ^1^H–^13^C HSQC correlations under standard conditions. To address this limitation, we developed a complementary methodology combining quantitative ^31^P NMR with a semiquantitative chelometric/UV–vis determination of catechol groups. Application to technical (kraft and organosolv) and analytical (milled wood and enzymatic) grade lignins from softwoods, hardwoods, and grasses revealed measurable catechol contents in all samples examined and indicated that catechol functionalities accounted for approximately 6–27% of the phenolic hydroxyl content conventionally assigned to guaiacyl units, especially, in technical and modified lignins. Additionally, model-compound data further revealed that 5-methoxy-catechyl structures can contribute to the region conventionally assigned to syringyl phenolic hydroxyls. Taken together, our results suggest that conventional ^31^P NMR assignments may overestimate guaiacyl and syringyl hydroxyls in lignins containing catechol groups.

## Graphical abstract

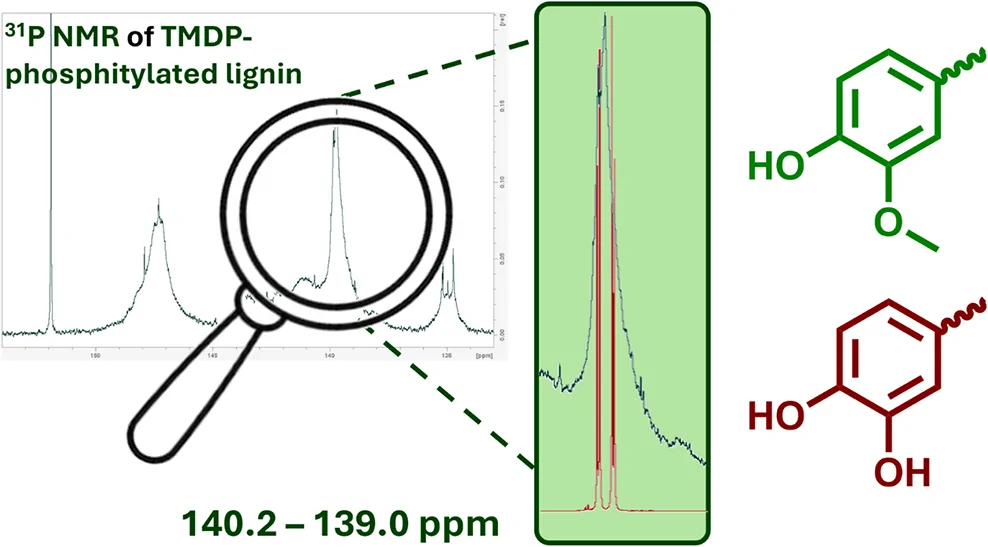

## Introduction

The traditional view of lignin as a branched polymer composed exclusively of *p*-coumaryl, coniferyl, and sinapyl alcohol-derived units^1^ has been revised in recent years, based on evidence of catechyl and 5-methoxy-catechyl units incorporated in nonwoody lignins.^2,3^ Relevant steps in this shift were represented by the isolation of linear, benzodioxane-rich lignin from seed coatings^4^ as well as by studies demonstrating the in vivo biosynthesis of catechyl-containing lignins or through plant genetic engineering.^5,6^

The presence of catechyl groups in lignins, however, is not solely associated with biosynthetic processes. Various chemical transformations can also alter the structure of lignin, including demethylation of phenolic units to yield catechol functionalities. This occurs, for example, during alkaline pulping processes such as soda and kraft pulping, where nucleophilic attack by hydroxide and hydrosulfide anions promotes demethylation, generating catechyl units alongside the release of methanol and mercapto-methanol, respectively.^7–11^ Enzymatic and microbial processes are also known to yield the same type of transformations,^12–14^ but they still remain marginally reported.

Catechol groups are of considerable relevance in lignocellulosic biomass processing and valorization, as they offer valuable opportunities for advanced applications owing to their strong adhesion and metal-chelating ability, arising from catecholate formation, and their antioxidant and antimicrobial potential, associated with the enhanced oxidation of catechols to o-quinones.^15,16^

Despite their importance, catechol groups remain comparatively difficult to quantify in lignins because of limitations associated with currently available analytical methods. Although catechyl units can be detected by ^13^C NMR spectroscopy, its routine application is hindered by (i) the large number of transients required to obtain spectra of sufficient quality for quantitative analysis, (ii) the substantial instrument time required, (iii) the associated operational costs, and (iv) the high amount of sample required.^17,18^ Furthermore, while catechyl resonances may be distinguished in guaiacyl-rich lignins, they become increasingly hidden in syringyl- or 5-methoxy-catechyl-rich samples, as demonstrated by Kringstad et al and Morck,^19^ owing to extensive signal overlap, thereby limiting the broader applicability of ^13^C NMR. Two-dimensional NMR, particularly heteronuclear single quantum coherence (^1^H–^13^C HSQC) NMR, has partially overcome these limitations and has enabled the structural elucidation of catechyl-rich lignins in seeds.^2^ However, its application to the analysis of catechol functionalities in technical lignins remains limited due to overlap of signals, and the occurrence of catechol groups in these materials has therefore received comparatively little attention.

By considering the dihydroxyphenyl nature of catechols, the use of ^31^P NMR spectroscopy after phosphitylation, the standard analytical tool in lignin analytics for lignin hydroxylated moieties,^20,21^ may appear obvious. However, despite its widespread use, its ability to distinguish and accurately quantify catechol functionalities has not been systematically evaluated. Consequently, the reliability of current analytical approaches for determining catechol contents in lignins remains largely unexplored.

Herein, we report a lignin model-compound-driven investigation of the use of ^31^P NMR spectroscopy following derivatization with 2-chloro-4,4′,5,5′-tetramethyl-1,3,2-dioxaphospholane (TMDP), together with ^1^H–^13^C HSQC NMR spectroscopy, for the detection and quantification of catechyl groups in lignins. Significant overlap between signals arising from catechol, guaiacyl, 5-methoxy-catechyl, and syringyl units was observed using both techniques, highlighting important limitations in their quantitative and semiquantitative application, particularly for technical and highly modified lignins. These early findings suggested that catechyl contents may be substantially underestimated in technical lignins. To address this challenge, we suggest a combined analytical strategy integrating ^31^P NMR spectroscopy with a highly selective differential UV–vis method based on iron­(III)-mediated chelation of catechol groups. This approach enabled the determination of a revised guaiacyl content based on catechol quantification for a range of technical and analytical lignins, providing improved insight into their distribution and origin. Moreover, it revealed the unexpected presence of trace catechol functionalities in conventional lignins, including *Picea excelsa* milled wood lignin, which were not detectable by conventional ^1^H–^13^C HSQC analysis.

## Methods

o-Catechol (1,2-dihydroxybenzene) (Merck, Finland) was recrystallized from toluene before use. *p*-Methyl catechol (1,2-dihydroxy-4-methyl-benzene) was purchased in analytical grade from Merck (Finland). Caffeic acid (3-(4-hydroxyphenyl)-prop-2-en-oic acid) (**1**) was purchased from Merck (Finland). Caffeoyl alcohol (3-(4-hydroxyphenyl)-prop-2-en-ol) (**2**) was prepared via DIBAL-H reduction of caffeic acid ethyl ester according to Quideau and Ralph.^22^ Dimeric models, such as catechyl-guaiacyl β-O-4′ dimer (4-(1,3-dihydroxy-2-(2-methoxyphenoxy)­propyl)­benzene-1,2-diol) (**3**), 5-methoxy-catechyl-guaiacyl β-O-4′ dimer (5-(1,3-dihydroxy-2-(2-methoxyphenoxy)­propyl)-3-methoxybenzene-1,2-diol) (**4**), and benzodioxane-dimer (4-(3-(hydroxymethyl)-2,3-dihydrobenzo­[b]­[1,4]­dioxin-2-yl)-2-methoxyphenol)) (**5**) were synthesized as reported by Nousiainen et al.^23^ Softwood kraft lignins were provided by UPM, BioPiva 100 (Finland) and FP Innovations (Canada); hardwood kraft lignin was provided by FP Innovations (Canada). Organosolv and enzymatic lignins from *Alnus glutinosa* and *Picea excelsa* were isolated based on standard protocols.^18^ The purity of lignins was determined via the Klason method, leading to contents, respectively, of 88%, 84%, and 91%. Wheat straw organosolv lignin was obtained from Fortum (Finland). Milled wood lignins from *Picea excelsa* and *Alnus glutinosa* were isolated according to the original method described by Björkman^24^ with purities, respectively, of 94% and 92% as determined by the semimicro Klason method. Analytical-grade *N*-hydroxy-5-norbornene-2,3-dicarboximide (NHND), chromium­(III) acetylacetonate, 2-chloro-4,4́,5,5́-tetramethyl-1,3,2-dioxaphospholane (TMDP), anhydrous iron­(III) chloride, sodium sulphite, acetic anhydride, and solvents used for both UV–vis measurements (dimethyl sulfoxide (DMSO), 2-methoxy-ethanol, and pyridine) and NMR measurements (*d*_6_-DMSO, and CDCl_3_) were obtained from Merck (Finland). Solvents used for NMR measurements had a deuteration degree >98%.

### NMR Spectroscopy Analyses

^31^P and ^1^H–^13^C HSQC spectra of lignin model compounds and lignins were recorded using a Bruker Avance NEO 400 NMR spectrometer equipped with a 5 mm double-resonance broadband probe (BBFO iProbe), according to established procedures.^18,20,21^ In the case of ^31^P analyses, 30 mg of lignin, or 10 mg of lignin model compound, were dissolved in 500 μL of a 1.6:1 (V/V) mixture composed of anhydrous pyridine and deuterochloroform. To the resulting solution, 100 μL of a 0.100 M internal standard solution, NHND, in a 1.6:1 (V/V) pyridine–deuterochloroform solution containing chromium­(III) acetylacetonate (acting as a relaxation agent, 5 mg mL^–1^) were added and the mixture was stirred up to complete dissolution. 100 μL of TMDP were then added; after stirring, the mixture was transferred into an NMR tube and analyzed. The ^31^P NMR spectra were recorded at room temperature using the standard proton-decoupling inverse-gated pulse sequence (*zgig*) with a 90° flip angle. 128 transients were acquired, with a pulse delay of 12 s, and spectral width of 100 ppm centered at 140 ppm. Spectra were referenced to the peak of phosphitylated residual water (132.2 ppm) and processed using TopSpin 4.4. In the case of ^1^H–^13^C HSQC, 70 mg of lignin or 10 mg of lignin model compound were dissolved in 500 μL of *d*_6_-DMSO. The resulting solution was analyzed using the hsqcetgpsisp2 pulse sequence obtaining a 1024 × 128 fid-matrix recording 36 transients. Spectral widths of 11.00 and 215.0 ppm were used for F2 and F1, with spectral centers at 5.50 and 105.0 ppm, respectively. Spectra were referenced to the peak of residual DMSO (39.5/2.50 ppm, δ_C_/δ_H_) and processed using TopSpin 4.4.

### Determination of Catechol Groups in Lignins

Catechol groups in lignin samples were determined via differential UV–vis spectroscopy based on the method introduced by Falkehag et al.^25^ with the following modifications. 35 mg of lignin were dissolved in a 5 mL volumetric flask with 4 mL of a mixture composed of 2-methoxy-ethanol/DMSO (8:2 v/v). To the resulting solution, 1 mL of 10 mM sodium sulfite in water was added, followed by dilution to the mark with additional 2-methoxy-ethanol/DMSO mixture. The resulting solution was divided into two 5 mL flasks, one acting as reference, the other as analyte. In both samples, 1 mL of a 2-methoxy-ethanol/water/pyridine solution (5:4:1 v/v) was added. To the analyte solution, 250 μL of a 10 mM ferric chloride solution in 2-methoxy-ethanol/DMSO (8:2 v/v) was added. Analyte and reference solutions were finally diluted with additional 2-methoxy-ethanol/DMSO solution to 5 mL. Solutions were filtered through a 0.22 μm PTFE filter and allowed to equilibrate in the dark for 2 h. The content of catechols (mmol g^–1^) of the sample was determined via difference spectroscopy at absorption maximum of 572 nm from the UV–vis spectra measured on a Shimadzu UV-2550 spectrophotometer using quartz cuvettes with an optical path of 10 mm. 2-methoxy-ethanol/DMSO was used as a reference and suitable dilutions with 2-methoxy-ethanol/DMSO were made to obtain absorbance values below 0.9 during measurements. If A_lignin, 572_ and A_lignin‑Fe, 572_ represent the absorbances at 572 nm of the reference lignin solution and of the lignin solution in the presence of Fe­(III) (the analyte), ε the molar absorptivity, and *l* the optical path in the utilized cuvette, the concentration of catechol in the lignin solution ([catechol]_lignin_) is given by the Lambert–Beer law:

1 $${[catechol]}_{lignin}=\frac{A_{lignin-Fe,572}-A_{lignin,572}}{\varepsilon \cdot l}$$

The molar absorptivity value for lignin catechol groups (1050 L•mol^–1^•cm^–1^) was determined as an average of various catechol group-containing model compounds (*o*-catechol, *p*-methyl catechol, protocatechuic aldehyde, and protocatechuic acid). The experimental procedure used for calculating it is described in Supporting Information, “Determination of average molar absorptivity and linear range of quantification”. To calculate the content of catechols in the original lignin, the ratio between the moles of catechols in the analyte solution (n_catechol, lignin_), estimated by multiplying the catechol concentration ([catechol]_lignin_) for the volume of the solution (5 mL = 0.005 L), and the amount of lignin in the solution (m_lignin, solution_), was used:

2 $$n_{catechol,lignin}\left(\mathrm{mmol}/g\right)=\frac{{[catechol]}_{lignin}\cdot 0.005L\cdot 1000}{\left(m_{lignin,solution}\right)}$$

The multiplying factor “1000” appearing in the numerator allows conversion of the moles content to millimoles. m_lignin, solution_ was determined from the initial mass of dissolved lignin (m_lignin, dissolved_) in 2-methoxy-ethanol/DMSO (5 mL) and finally corrected by a 1:2 dilution factor:

3 $$m_{lignin,solution}=\frac{m_{lignin,dissolved}}{5\mathrm{mL}}\cdot \frac{1}{2}\mathrm{mL}=\frac{m_{lignin,dissolved}}{10}$$

If a different dilution factor is used (1:x), the previous equation to calculate m_lignin, solution_ becomes

4 $$m_{lignin,solution}=\frac{m_{lignin,dissolved}}{5\cdot x}$$

### Lignin Acetylation

Acetylation was performed according to Adler et al.^26^ with the following modifications to demonstrate the selectivity of iron­(III) chelation by terminal catechol units. 300 mg of softwood kraft lignin, previously dried under vacuum at 40 °C for 48 h, were dissolved in 8 mL of a 1:1 (v/v) mixture of acetic anhydride and anhydrous pyridine, and stirred for 48 h at room temperature. The reaction was quenched with 20 mL of ethanol, and the solvent was evaporated under reduced pressure. The addition of ethanol followed by evaporation was repeated seven times. The resulting acetylated lignin was then dried under vacuum at 40 °C for 48 h.

## Results and Discussion

### Catechyl Units in ^31^P and ^1^H-^13^C HSQC Spectra

To evaluate whether catechol functionalities contribute to the typical hydroxyl regions of lignin ^31^P NMR spectra, a series of catechol-containing model compounds were synthesized (Figure 1).

**Figure 1:**
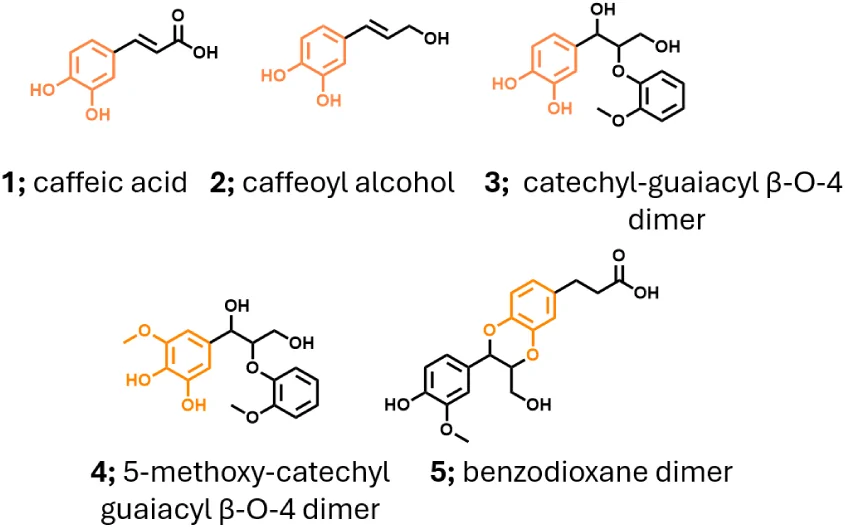
Synthesized catechyl-containing lignin model compounds.

The ^31^P NMR spectra of model compounds **1**-**4** and commercially available *o*-catechol and *p*-methyl catechol were recorded following standard phosphitylation with TMDP, and the chemical shifts of phosphitylated hydroxyl moieties are summarized in Table 1. Comparison with the previously reported chemical shift range for TMDP-derivatized guaiacyl hydroxyl groups in lignin (139.0–140.2 ppm)^27–29^ reveals substantial overlap with signals arising from catechyl hydroxyl groups.

**Table 1:** ^31^P Chemical Shifts of TMDP-Labeled Lignin Models Containing Terminal Catechyl Units (**1** to **4**)­^a^

**Compound**	**Aliphatic OH (ppm)**	**Phenolic OH (ppm)**	**-COOH (ppm)**
**Guaiacyl in lignins** ^20,27–30^	-	140.2–139.0	-
**Syringyl in lignins** ^20,27,30^	-	143.2–142.5	-
***o*** **-catechol**	-	139.0^b^	-
		139.4	
***p*** **-methyl catechol**	-	138.9^b^	-
		139.2	
**1**	-	138.6^b^	135.0
		139.6	
**2**	148.0	138.9^b^	-
		139.2	
**3**	147.3	138.7^b^	-
	148.2	139.1	
**4**	147.2	138.8^b^	-
	148.2	142.5	

a Spectra are reported in Supporting Information (Figures S3–S7).

b The ^31^P NMR spectra of these phosphitylated model compounds show the phenolic hydroxyl groups as doublets; the reported chemical shifts correspond to the center of each doublet. As the inverse-gated pulse sequence used for spectral acquisition suppresses ^1^H–^31^P coupling, the observed doublets were attributed to long-range coupling of ^31^P nuclei (^5^J_P–P_).

These observations demonstrate that catechyl hydroxyls cannot be unambiguously distinguished from guaiacyl hydroxyls on the basis of ^31^P NMR chemical shifts alone and therefore contribute to the apparent guaiacyl hydroxyl content. This issue is particularly relevant for technical lignins (e.g., kraft and soda lignins) and enzymatically modified lignins, where catechol functionalities arise from demethylation reactions promoted by nucleophilic attack or other degradation pathways.^8–10^ Moreover, the same limitation extends to the emerging class of catechyl-rich lignins in seeds and genetically modified plants,^4–6^ which have attracted considerable interest since their discovery owing to their distinctive structural and physicochemical properties.

Model compound **4** provided further evidence that also 5-methoxy-catechyl- structures influence multiple regions of the ^31^P NMR spectrum. In fact, two signals of comparable intensities were observed at 138.8 and 142.5 ppm. According to conventional assignments,^30^ these resonances would typically be attributed to guaiacyl and 5-substituted or condensed phenolic hydroxyl groups, respectively. This finding indicates that not only catechyl units of 3,4-dihydroxyphenyl type but also 5-substituted 3,4-dihydroxyphenyl structures contribute to multiple regions of the ^31^P NMR spectrum of lignin, thereby affecting the interpretation of spectra and the quantification of guaiacyl, and condensed phenolic hydroxyl groups. This phenomenon is particularly relevant in hardwood kraft lignins, where the methoxy group located in the ortho position to the phenolic hydroxyl of terminal syringyl moieties can undergo cleavage through reaction with hydrosulfide anions under pulping conditions, leading to the formation of 5-methoxy-catechyl units. Because of substantial overlap and the relatively minor contribution of catechyl hydroxyls, reliable spectral deconvolution is not feasible.

The presence of catechyl units in catechyl-rich lignins has been frequently assigned by ^1^H–^13^C HSQC analysis,^31^ as demonstrated for *V. planifolia* following acetylation.^32^ This is facilitated by the unique monolignol composition, primarily consisting of caffeoyl alcohol. However, such assignments cannot necessarily be directly extended to technical lignins, in which signals arising from guaiacyl, syringyl, and *p*-hydroxyphenyl units, as well as from other unsaturated substrates, may overlap extensively. Therefore, the unambiguous identification of catechyl units in technical lignins by HSQC alone may be challenging. This ambiguity was confirmed by the ^1^H–^13^C HSQC analysis of models 1 to 5, which revealed further analytical complexity. Examination of the aromatic -^1^H-^13^C correlation signals, summarized in Table 2, showed substantial overlap between catechyl- and guaiacyl-derived resonances. In particular, the C_2_/H_2_ and C_5_/H_5_ correlations of the catechol nucleus fall within the region typically assigned to guaiacyl C_5_/H_5_ correlations, while the C_6_/H_6_ correlations also occur in close proximity to those of guaiacyl units. For model compound **4**, additional signal overlap was observed, with the C_2_/H_2_ and C_6_/H_6_ correlations appearing nearly coincident and located close to the region commonly assigned to syringyl units. This overlap is evident in Figure 2, where the aromatic regions of the ^1^H–^13^C HSQC spectra of softwood and hardwood kraft lignins are overlaid with the ^1^H–^13^C HSQC spectra of *p*-methyl catechol and the 5-methoxy-catechyl-guaiacyl β-O-4′(**4**) dimer.

**Table 2:**
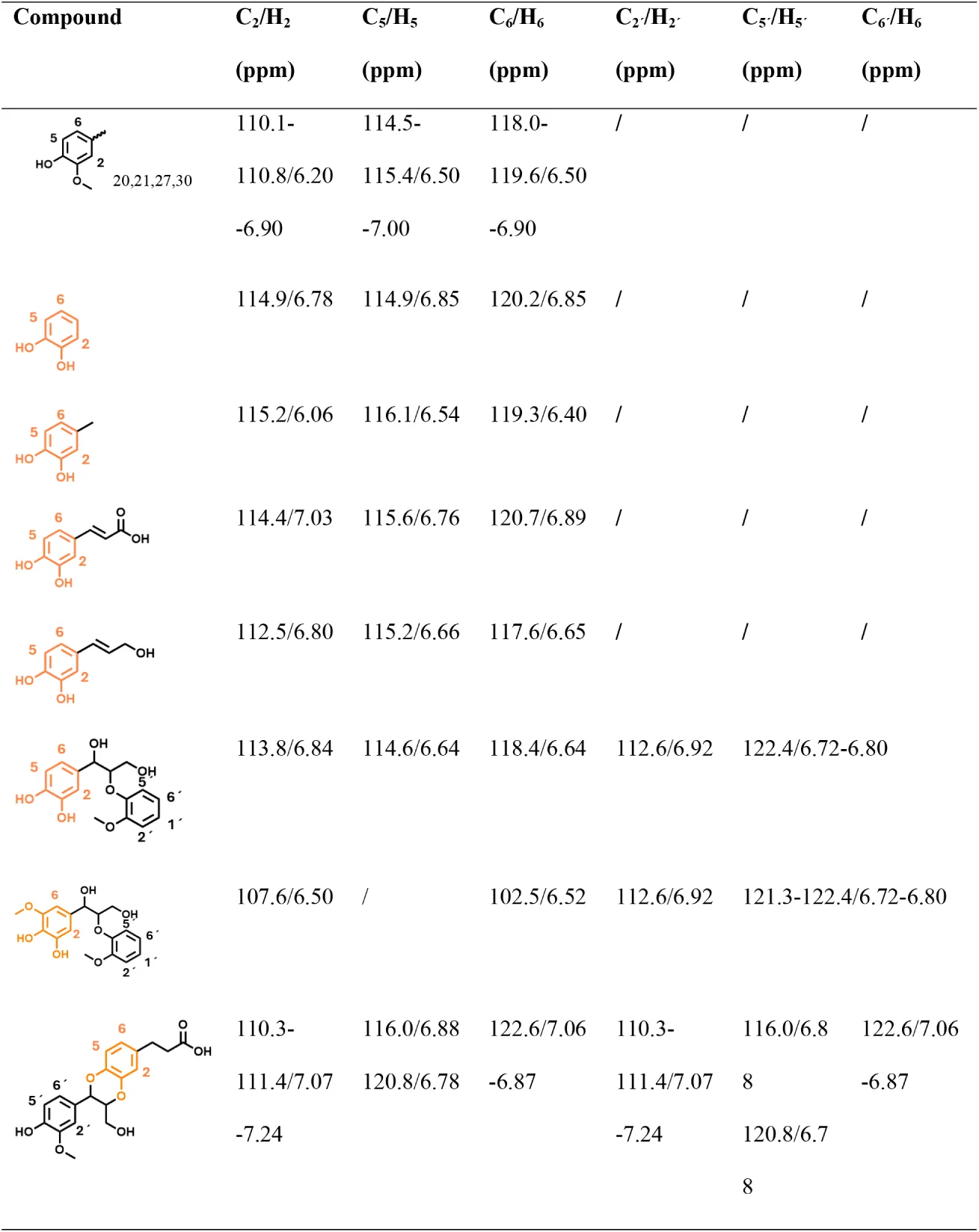
Chemical Shifts of Aromatic Nuclei in ^1^H–^13^C HSQC NMR Spectra^a^

a Spectra are reported in Supporting Information (Figures S8–S12).

**Figure 2:**
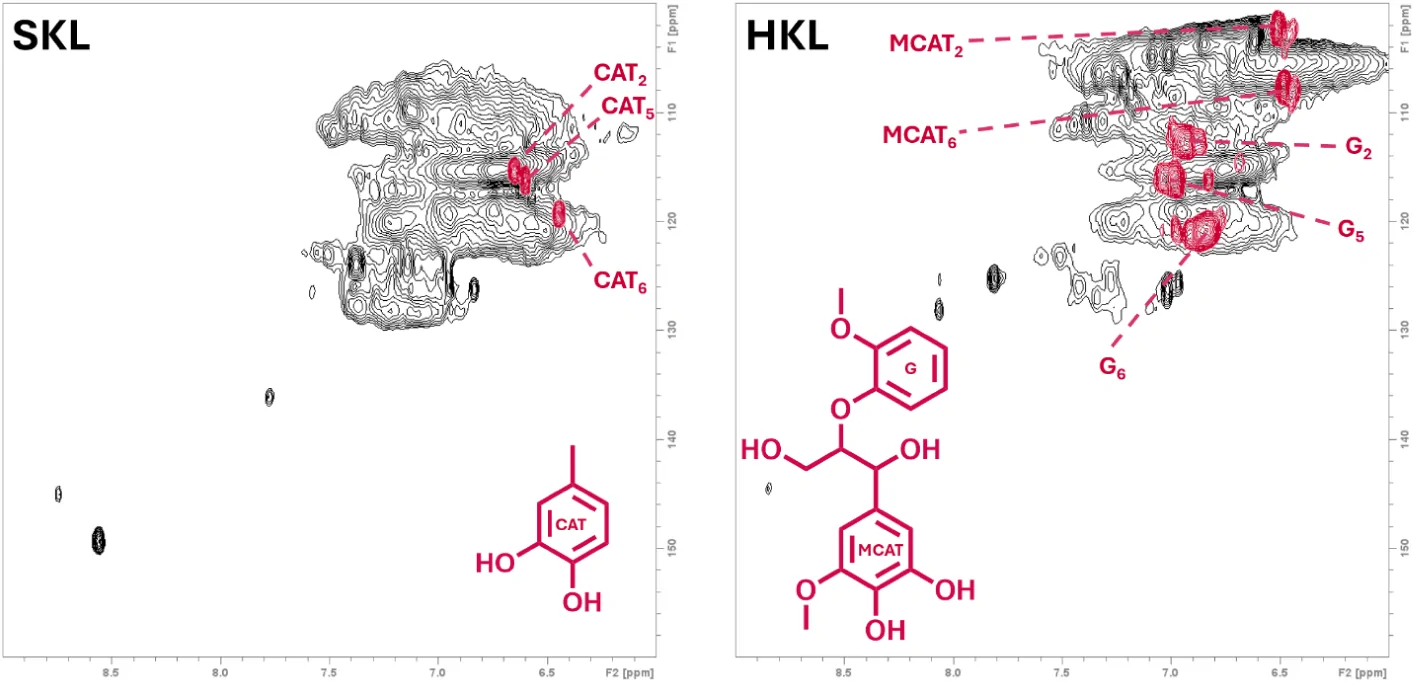
Overlapped aromatic areas (6.0 < δ_H_ < 9.0 and 100.0 < δ_C_ < 160.0 ppm) of the ^1^H–^13^C HSQC spectra of softwood kraft lignin (SKL) with *p*-methyl catechol (left) and hardwood kraft lignin (HKL) with model **4** (right).

These results indicate that unequivocal identification of catechyl units based solely on aromatic ^1^H–^13^C HSQC correlations may be challenging, not only in structurally complex lignins but also in relatively simpler samples such as softwood kraft lignin. Although structural assignments are frequently supported by the analysis of the oxygenated aliphatic region, chemical modifications affecting ether linkages, including alkylation, acylation, and degradation during lignin isolation or processing, may substantially alter the corresponding signals. Consequently, conventional ^1^H–^13^C HSQC assignment criteria may not reliably distinguish catechyl- and guaiacyl-aromatic correlations, potentially leading to overlooked catechyl-containing structures and an overestimation of the guaiacyl content. Consistent with this interpretation, catechyl signals are rarely discussed in published ^1^H–^13^C HSQC spectra of softwood kraft lignins, despite independent evidence for their presence.^33^

### On the Determination of Catechols in Lignins via the Chelometric/UV–Vis Method

Because neither ^31^P nor ^1^H–^13^C HSQC NMR provided unambiguous discrimination between guaiacyl, syringyl, and catechol functionalities, we developed a complementary analytical approach combining ^31^P NMR and UV–vis spectroscopy.

UV–vis spectroscopy has been widely applied for the study of phenolic hydroxyl groups via differential spectroscopy since the early days of lignin analytics, as described by Goldschmid in 1954.^34^ Specifically, by profiting from the differences in absorbance of equimolar lignin solutions under acidic and alkaline conditions, it is possible to determine the content of certain hydroxylated moieties exhibiting different absorbance under their protonated (phenolic) and unprotonated (phenolate) forms.^35,36^

Catechol groups in lignin selectively form iron­(III)–catecholate complexes under alkaline buffered conditions.^25,37^ These complexes are characterized by an intense gray-blue color, with a distinctive absorption maximum in the 560–590 nm region. No other lignin-related species are expected to be affecting this spectral region, as it appears evident from the lack of absorbance maxima in the UV–vis spectra of lignins. On this basis, catechol functionalities were first quantified by differential UV–vis spectroscopy by Falkehag et al.,^25^ following the mathematical approach originally described by Aulin–Erdtman.^38^ Similarly, here we quantified the content of catechols by differential spectroscopy, profiting from the formation of the iron­(III) lignin-catecholate complexes. Specifically, we recorded the UV–vis spectra of lignin samples in the presence and absence of iron­(III), with trace amounts of pyridine to enhance the absorption intensity of the maximum at 560–590 nm through an auxochromic effect. Although the stronger base 2,4,6-trimethylpyridine (p*K*_b_ = 6.6) was recommended in literature,^39^ we here used pyridine (p*K*_b_ = 8.8) because we observed that it offers a better auxochromic effect, allowing to reduce the lignin amount for the analyses from the original 70–75 mg to 35 mg.

The formation of a maximum in absorbance in the difference curve is evident from Figure 3a, where the absorptivity curves recorded in the presence and absence of iron­(III) for *Picea excelsa* milled-wood lignin and differential absorption curve, are reported.

**Figure 3:**
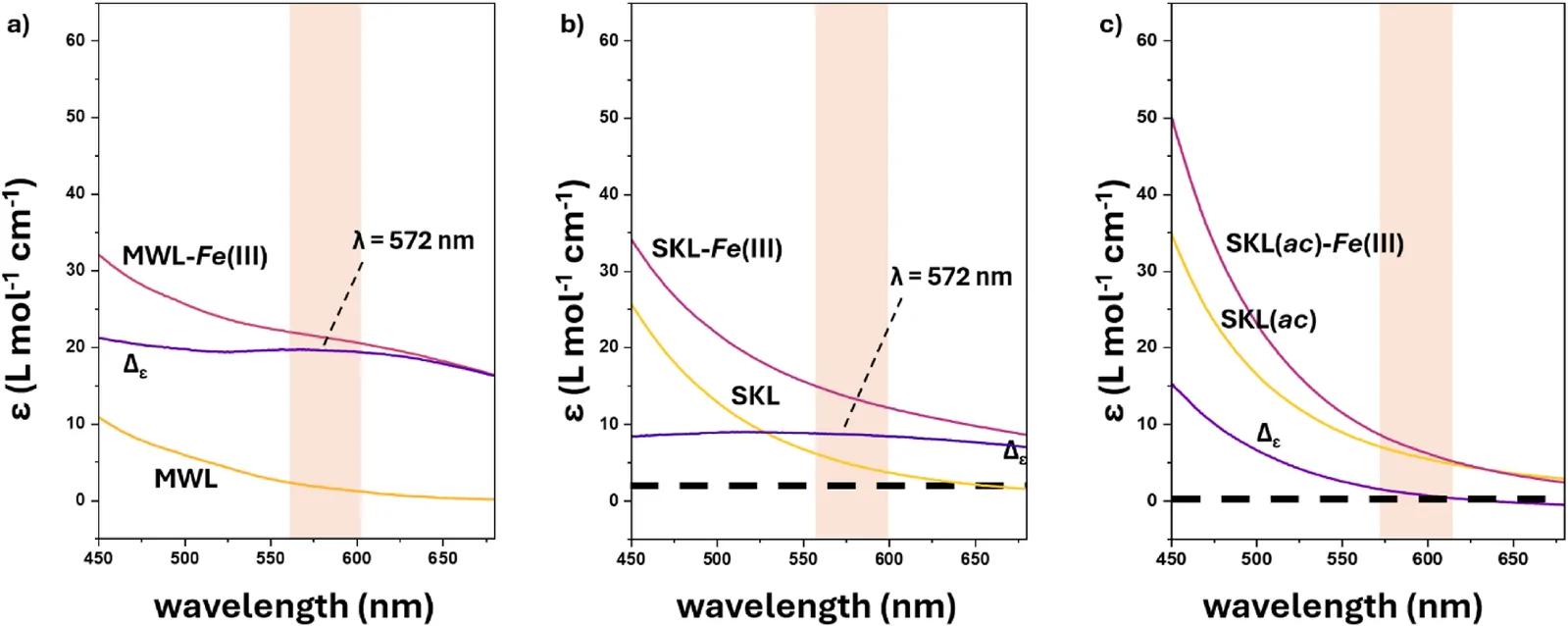
Molar absorptivity (ε) vs wavelength of equimolar solutions for (a) *Picea excelsa* milled wood lignin in the presence (MWL-Fe­(III), magenta curve) and absence (MWL, yellow curve) of Fe­(III), (b) softwood kraft lignin (SKL) in the presence (SKL-Fe­(III), magenta curve) and absence (SKL, yellow curve) of Fe­(III), and (c) acetylated softwood kraft lignin in the presence (SKL­(ac)-Fe, magenta curve) and absence (SKL­(ac), in yellow) of Fe­(III). In the difference curves (Δ_ε_, in purple), the maxima appearing for MWL and SKL spectra, respectively, at 570 nm and 572 nm, are attributed to the formation of an iron­(III)-catecholate complex.

To validate the selectivity for the absorption of iron­(III)–catecholate complexes in the 560–590 nm region^25^ and to exclude potential interference arising from possible iron­(III)-carboxylate species absorbing between 280 and 445 nm,^40–42^ the differential UV–vis curves for softwood kraft lignin before and after quantitative acetylation were compared. Both phenolic and aliphatic hydroxyl groups were fully acetylated in the esterified sample, with preservation of carboxylate groups, as verified by ^31^P NMR, which showed the disappearance of signals in the related spectrum in the 138–149 ppm region. The differential curves obtained for the pristine and acetylated softwood kraft lignin via our method are depicted in Figure 3b and c. In pristine softwood kraft lignin, the differential curve revealed the absorption maximum at 572 nm, consistent with the formation of iron­(III)–catecholate complexes. In contrast, although a hypsochromic shift was observed in acetylated softwood kraft lignin, probably resulting from chelation via residual free carboxylic groups, no distinct absorption maximum within the 560–590 nm range appeared. These results confirmed that the absorption band at approximately 570 nm originates predominantly from catechol functionalities under the experimental conditions employed. Under the described conditions for the analytical determination of catechols in lignins, the contribution of carboxylic acid chelation is expected to be marginal compared with that arising from catechols. This conclusion is supported by the comparison of the characteristic thermodynamic stability constants (β) for iron­(III) with catechols and carboxylate species. Indeed, for complexes with a 1:1 Fe­(III) molar ratio, log β values are around 20 under alkaline conditions for catechols, whereas those reported for carboxylate complexes range from 2.5 to 4.5 under acidic conditions. These conditions differ substantially from the alkaline-buffered conditions used for the estimation of catechols, under which the formation of Fe­(III)–carboxylate complexes is therefore not expected.^43,44^ To further assess these conclusions experimentally, UV–vis spectra of equimolar solutions of protocatechuic acid, containing both catechol hydroxyls and a carboxylic group, and veratric acid, bearing two methoxy groups in lieu of the two catechol hydroxyls of protocatechuic acid with preserved carboxyl group, were recorded under the same conditions used for the formation of lignin/iron­(III) complexes, both in the presence and absence of iron­(III). The corresponding spectra are reported in Figure S13 in Supporting Information. In the presence of iron­(III), protocatechuic acid exhibited a distinct absorption band attributed to the catechol-iron­(III) complex, whereas veratric acid does not exhibit any appreciable absorption in the 550–700 nm region under the same conditions. Since veratric acid retains the carboxylic group while lacking the catechol moiety, the absence of a detectable band in this spectral region indicated that carboxylate complexation, if occurring, does not make an appreciable contribution to the absorption band assigned to catechol-Fe­(III) complexes around 570 nm.

### On the Estimation of the Molar Absorptivity for Catechols in Lignins, Range of Linearity, LOD, and LOQ of the Chelometric/UV–Vis Method

The primary limitation of the chelometric/UV–vis methodology presented here lies in the challenge of rigorously determining the absorptivity of the iron­(III)-lignin catecholate complex, owing to their various types of surrounding chemical environments. To address this limitation, an average absorptivity value was originally determined using standard solutions of various catechol-containing monomeric compounds (*o*-catechol, pyrogallol-1-methyl ether, *p*-methyl catechol, and 3,5-dimethyl catechol), yielding a value of 1100 L mol^–1^•cm^–1^.^25^ In the present work, we recalculated the average absorptivity using different catechol models (*o*-catechol, *p*-methyl catechol, protocatechuic aldehyde, and protocatechuic acid). The choice to include protocatechuic acid among the models was motivated by the need to account for the possible, albeit minimal, combined contribution of catechol and carboxylate chelation of iron­(III). A detailed explanation on the calculation of the molar absorptivity is presented in Supporting Information. An average of ε_572_ 1050 ± 240 L mol^–1^•cm^–1^ was obtained, consistent with results from similar models.^39^ Given the substantial variability among individual model compounds, the value should be regarded as an “operational average” rather than a “sample-independent” molar absorptivity. Consequently, the resulting catechol contents are best interpreted as estimates suitable primarily for comparative purposes. Although this approximation provides quantitative estimates with an uncertainty based on the molar absorptivity variation of approximately ± 23%, to our knowledge, no currently available method allows detection of catechol structures in lignin without degradation. In this context, the chelometric/UV–vis method presented here offers a simple approach to estimate the content of 3,4-dihydroxyphenyl structures in lignin samples. Its application is particularly advantageous when comparing lignins derived from the same type of biomass, for which the method provides a consistent basis for assessing differences in catechol content.

The linearity range of the chelometric/UV–vis method was estimated using *p*-methyl catechol as the reference model for terminal catechol units in lignin (details in Supporting Information). The linearity range was estimated over a concentration range from 0.015 mM to 0.400 mM (R^2^ = 0.9969). Within the same concentration range, the iron­(III) catecholate complexes of *o*-catechol, protocatechuic acid, and protocatechuic aldehyde showed comparable linearity, with R^2^ > 0.9969, thereby supporting the validity of the estimated range.

The limits of quantification (LOQ) and detection (LOD) were experimentally determined using *p*-methyl catechol as the reference compound (details are provided in Supporting Information). The LOQ was calculated to be 7.29 × 10^–3^ mM, whereas the LOD was determined to be 2.40 × 10^–3^ mM.

The chelometric/UV–vis method was further validated by spiking experiments, which revealed that the method quantified the catechol content within 92–96% of the theoretical values. Specifically, three different solutions of a softwood kraft lignin, with a catechol content estimate by the chelometric/UV-vis method of 0.23 mmol g^–1^, were prepared and spiked with increasing amounts of a 9.67 mM solution of *p*-methyl catechol. The catechol concentrations in the resulting solutions were then determined using the chelometric/UV–vis method, and the results are reported in Table S1 in Supporting Information.

Overall, the present chelometric/UV–vis method appears particularly more convenient than traditional quantitative ^13^C NMR spectroscopy, as the practical sample preparation and utilization time for the instrument time totals less than half an hour per sample. Additionally, it not only reduces the costs of the overall analyses but has also the advantages of (a) utilizing broadly available UV–vis spectrophotometers instead of expensive instruments like NMR spectrometer, (b) requiring smaller amounts of analyte (35 mg or less versus typical 100–200 mg in quantitative ^13^C NMR^17,45,46^ ), and (c) overcoming the issue in peak overlapping with typical syringyl units.

### Combined Chelometric/UV–Vis with ^31^P NMR Spectroscopy

The combination of the present chelometric/UV–vis method with ^31^P NMR spectroscopy allows one to (a) adjust the content of guaiacyl taking into account the content of 3,4-dihydroxy-phenyl units and (b) estimate the content of total free monohydroxylated phenolic units.

Once the catechol content has been estimated via the chelometric/UV–vis method, an approximate correction for the apparent guaiacyl hydroxyl content for softwood lignins was obtained assuming that catechol functionalities appear predominantly as 3,4-dihydroxyphenyl structures. These patterns, exhibiting two vicinal hydroxyl groups, are bidentate phenolic ligands. Internal proto-catechol units (4-etherified species such as 4-alkoxy-3-hydroxyphenyl or 4,5-dialkoxy-3-hydroxyphenyl rings) exhibit only one free hydroxyl group, in the *o*-position to the etherified phenolic hydroxylated moiety. Proto-catechol species are monodentate phenolic ligand centers and therefore they cannot form the same type of complexes generated by 3,4-dihydroxyphenyl units with iron­(III), owing to different denticity and coordination mode. Under these assumptions, the content of catechols estimated by the chelometric/UV–vis method can be reasonably considered to represent terminal 3,4-dihydroxyphenyl units, allowing the apparent guaiacyl hydroxyl content (G_31P_ NMR) to be adjusted by subtracting twice the catechol contribution (*C*) according to eq 1:

5 $${G_{adjusted}}^{softwood}\left(\mathrm{mmol}\cdot g^{-1}\right)=G_{31PNMR}-2\cdot C$$

The factor of 2 arises because each catechol unit of 3,4-dihydroxyphenyl type contains two hydroxyl groups, each of them reacting with TMDP under typical phosphitylation conditions, thus contributing twice to the content of guaiacyl hydroxyl groups. This relationship is illustrated in Figure 4, which compares the stoichiometry of TMDP derivatization of typical phenolic lignin units (G, S, or H) with that of catechyl units.

**Figure 4:**
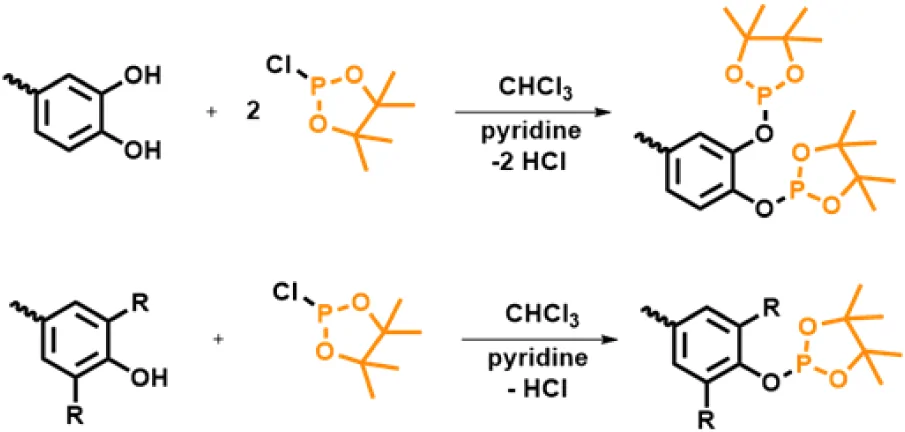
Comparison of the different stoichiometries of TMDP phosphitylation of catechyl rings (*up*), and typical terminal lignin phenolic units (*down*, where R = −H or −OCH_3_).

Because 4-etherified catechols phosphitylated with TMDP are expected to resonate in the guaiacyl region, they may bias our approximation and underestimate the guaiacyl content. Additionally, given that steric hindrance (e.g., in biphenyl-type motifs) limits TMDP access to phenolic hydroxyls , 4-etherified catechols may also phosphitylate incompletely.^47^ Further work is needed to quantify their accessibility and phosphitylation rates and to determine their effect on the adjusted guaiacyl content.

In the case of hardwood lignins, the interpretation of catechol contributions is inherently more complex than in softwood ones because both 3,4-dihydroxyphenyl and 3,4-dihydroxy-5-methoxyphenyl structures may be present, particularly in kraft lignins where demethylation reactions can occur on both guaiacyl and syringyl terminal units. As demonstrated by model compound **4** (Table 1), 3,4-dihydroxy-5-methoxyphenyl structures contribute simultaneously to the spectral regions conventionally assigned to guaiacyl and syringyl phenolic hydroxyl groups in ^31^P NMR spectrum. Consequently, the straightforward correction proposed for softwood lignins cannot be directly extended to hardwood samples. At present, no analytical technique enables the quantitative differentiation of catechyl and 5-methoxy-catechyl units in lignins with sufficient reliability. Therefore, a rigorous correction of the apparent guaiacyl hydroxyl content is currently not feasible. To provide an approximate basis for comparison among hardwood lignins, we introduced an illustrative adjustment assuming equal contributions of catechyl and 5-methoxy-catechyl structures. Under this assumption, only half of the catechyl group content (C), estimated as per the chelometric/UV–vis method, contributes to the apparent guaiacyl groups (*G_31PNMR_*) so the adjusted guaiacyl content (*G_adjusted_^hardwood^*) can be determined as per eq 2:

6 $${G_{adjusted}}^{hardwood}\left(\mathrm{mmol}\cdot g^{-1}\right)=G_{31PNMR}-C$$

No attempts to correct the syringyl content in a similar manner were made, owing to the complex nature of the cluster of peaks pertaining to condensed phenolic units, where syringyl phenolics overlap with both diaryl ethers and biphenyls, as previously demonstrated by Balakshin et al.^47^

Finally, because grass lignins also contain a substantial amount of syringyl structures that may undergo demethylation, the same approximation adapted for hardwood lignins is expected to provide a reasonable estimate. Consequently, the adjustment of the apparent guaiacyl content (*G_adjusted_^grass^*) may be calculated analogously as to eq 2 with eq 3:

7 $${G_{adjusted}}^{grass}\left(\mathrm{mmol}\cdot g^{-1}\right)=G_{31PNMR}-C$$

A more reliable estimation of the distribution of 3,4-dihydroxyphenyl and 3,4-dihydroxy-5-methoxyphenyl units in hardwood and grass lignin can be obtained if the syringyl-to-guaiacyl ratio (S/G) of the sample is available. This ratio is frequently determined using either spectroscopic methods, such as from the ^1^H–^13^C HSQC spectra, or degradative techniques, such as nitrobenzene oxidation or thioacidolysis. Consequently, eqs 2 and 3 can be reformulated to incorporate the effective guaiacyl ratio (*r_G_*), calculated from the S/G ratio (details are presented in Supporting Information), resulting in a more reliable estimate of the adjusted guaiacyl content, as expressed in eq 4:

8 $${G_{adjusted}}^{hardwood\;or\;grass}\left(\mathrm{mmol}\cdot g^{-1}\right)=G_{31PNMR}-2\cdot \left(r_{G}\right)\cdot C$$

Here again, the factor of 2 is included to account for the two ^31^P resonances measured for the signal corresponding to each catechol unit. eq 4 is considered the more reliable approach for hardwood and grass lignins, whenever an experimentally determined S/G ratio is available. Nevertheless, the approximations introduced for hardwood and grass lignins should not be interpreted as absolute corrections to the guaiacyl hydroxyl content. Rather, they provide a practical framework for the comparative evaluation of catechol content in lignins deriving from the same feedstocks subjected to different processing conditions.

An additional advantage of the chelometric/UV–vis method is that, by accounting for catechol functionalities, it also enables the estimation of the content of monohydroxyl phenols (*M*_OH_), including eventual proto-catechol units. Specifically, the contribution of dihydroxylated phenols can be subtracted from the total phenolic hydroxyl content determined by ^31^P NMR (*P*_OH,*31P NMR*_). The corrected value is obtained by subtracting twice the catechol content estimated by the chelometric/UV–vis method. In the present case, the catechol content can be reasonably assumed to derive from both 3,4-dihydroxyphenyl and 3,4-dihydroxy-5-methoxyphenyl units, as described by eq 5:

9 $$M_{OH}\left(\mathrm{mmol}\cdot g^{-1}\right)=P_{OH,31PNMR}-2\cdot C$$

The factor of 2 accounts for the fact that both 3,4-dihydroxyphenyl and 3,4-dihydroxy-5-methoxyphenyl units react with TMDP in the same manner, leading to two phosphitylated phenolic hydroxyl groups per aromatic ring (Figure 4). Since model compound studies (Table 1) demonstrated that both 3,4-dihydroxyphenyl and 3,4-dihydroxy-5-methoxyphenyl units give signals in the phenolic region of the ^31^P NMR spectrum of phosphitylated lignins, subtracting twice the catechol content estimated via the chelometric/UV–vis method accounts for the two phenolic hydroxyl groups associated with both catechol units, thereby allowing to determine the content of monohydroxyl phenols in the analyte.

### Application of the Chelometric/UV–VVis-^31^P NMR Approach to Real Lignin Samples

The combined chelometric/UV–vis method with traditional ^31^P NMR spectroscopy was applied at least in triplicate to nine different lignin samples, including technical- and analytical-grade preparations. Catechol concentrations in the analyzed solutions of lignins analyzed ranged from 0.0250 to 0.0807 mM, comprised within the linearity range of the method. The catechol contents in the samples, determined using the average molar extinction calculated from catechol models and the lignins concentrations, are listed in Table S2 in Supporting Information. The latter was used to calculate the adjusted content of the guaiacyl group. Results are reported as well in Table S2 in Supporting Information and depicted in Figure 5a. The monohydroxyl phenol content was also calculated, and the results are depicted in Figure 5b and the numerical data summarized in Table S3 in Supporting Information.

**Figure 5:**
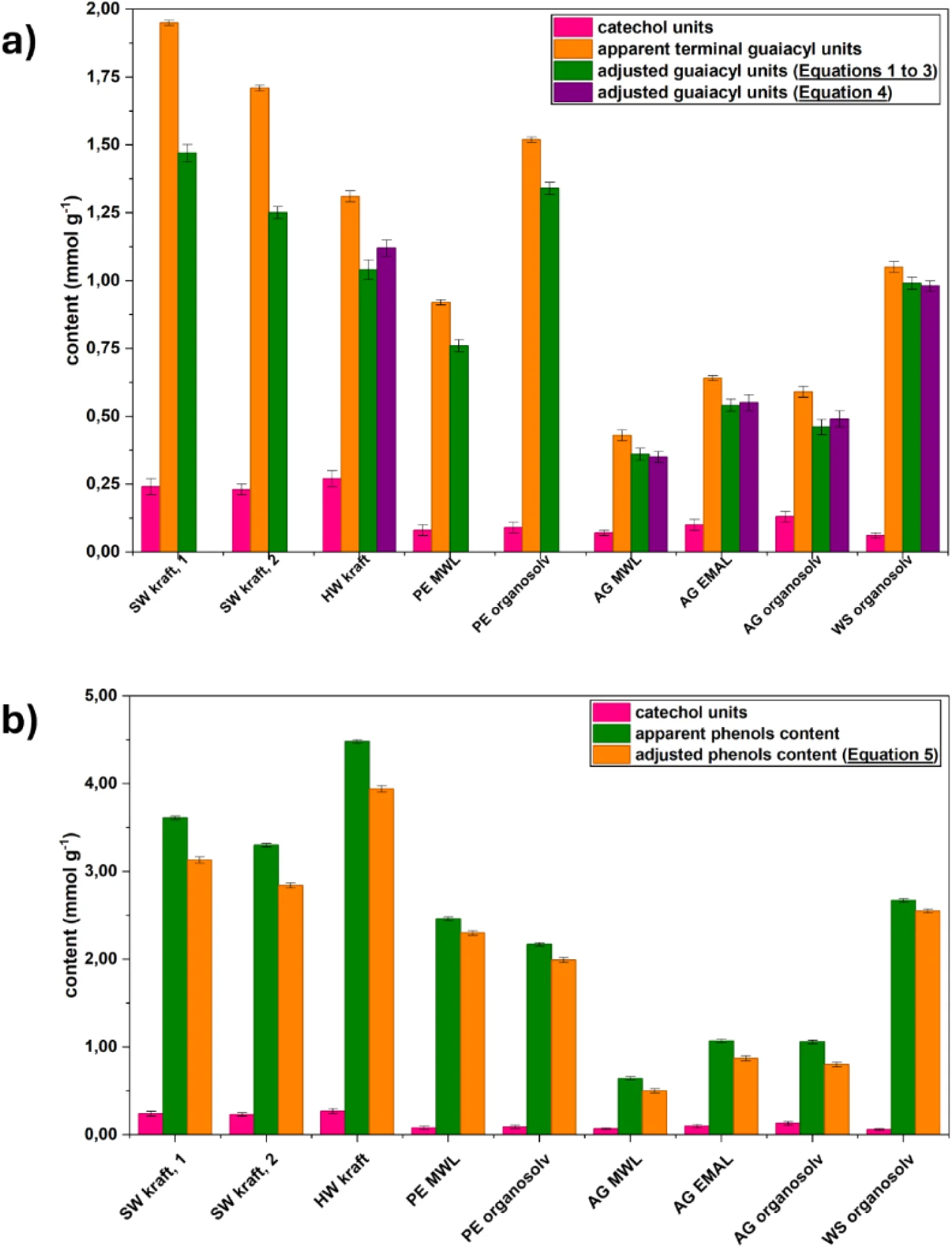
Comparison of the catechol content, estimated via the chelometric/UV–vis method, with (a) guaiacyl content, determined by ^31^P NMR, and adjusted guaiacyl hydroxyl content for different lignins, and (b) total phenolic content, determined by ^31^P NMR, and adjusted phenolic hydroxyl content to account only for monohydroxyl phenols. Details on the calculation of the error in the analyses are described in section entitled Error propagation in the measurements in Supporting Information.

The chelometric/UV–vis method revealed the presence of catechol groups in all samples, with variations in line with wood species and their isolation methods. The apparent guaiacyl groups were affected between 6 and 27% by 3,4-dihydroxyphenyl units.

Softwood kraft lignin samples from different producers, SW kraft 1 and 2, revealed similar catechol contents, 0.24 ± 0.03 and 0.23 ± 0.02 mmol g^–1^, corresponding respectively to the 24 ± 3% and 27 ± 2% of the apparent guaiacyl hydroxyl groups determined by ^31^P NMR. These results, highlighting a significant overestimation, probably arise from demethylation reactions occurring during kraft pulping.^10,11^ Catechols accounted for 13 ± 1% and 14 ± 1% of total phenolic units, leading to adjusted-phenol contents representative only of the monohydroxyl phenols of 3.13 ± 0.4 and 2.84 ± 0.03 mmol g^–1^. Two analytical-grade softwood lignins were isolated from *Picea excelsa*, milled wood lignin (PE MWL) and 1,4-dioxane acidolysis lignin (PE organosolv). PE MWL displayed a catechol content of 0.08 ± 0.01 mmol g^–1^, corresponding to 17 ± 2% of the apparent guaiacyl content. The origin of these structures cannot be unambiguously established from the present data. Earlier findings on *Picea excelsa* claimed the possible incorporation of catechol units during the biosynthesis of its lignin, with a previously estimated content of 0.05 mmol g^–1^.^39^ However, catechol structures may also arise from mechanochemical degradation during the milling required for the isolation of milled wood lignin, potentially involving partial demethylation and the consequent appearence of terminal catechol structures. Independent structural evidence is required to establish the origin of these catechol units. A comparable content of catechol units was also detected in PE organosolv lignin, 0.09 ± 0.01 mmol g–1, representing 12 ± 1% of the apparent guaiacyl content. This finding corroborates results from PE MWL, which suggest possible incorporation of catechyl units during PE lignin biosynthesis; however, in the present case, the acidolysis conditions used for isolation may likewise induce partial demethylation, as previously observed when cooking lignin in acidic 1,4-dioxane.^48^ Estimated catechol content affected the total phenolic contents in PE MWL for the 6.5 ± 0.8%, while in the case of PE organosolv of the 8.3 ± 0.9% of total phenolic hydroxyls. The adjusted monophenolic hydroxyl contents were calculated as being respectively of 2.30 ± 0.02 and 1.99 ± 0.02 mmol g^–1^.

For the analyzed hardwood and grass lignins, eqs 2, 3, and 4 were used to estimate the contribution of catechol units to the apparent guaiacyl content. It was consistently observed that as the S/G approximate to 1, the results obtained from eq 4 approximate those from eqs 2 and 3, validating the possibility to assume, under certain circumstances, that equimolar amounts of terminal 3,4-dihydroxyphenyl and 3,4-dihydroxy-5-methoxyphenyl units appear in the samples. In the case of hardwood kraft lignin, the chelometric/UV–vis method revealed a content of 0.27 ± 0.03 mmol g^–1^ of terminal catechol units. Assuming an equimolar amount of 3,4-dihydroxyphenyl and 3,4-dihydroxy-5-methoxyphenyl units in the sample, using eq 2, a 21 ± 2% overestimation of the guaiacyl content was estimated, resulting in an adjusted guaiacyl content of 1.04 ± 0.04 mmol g^–1^. Using the S/G ratio (1.86), as determined from the ^1^H–^13^C HSQC spectrum, and eq 4, the adjusted guaiacyl content was estimated as 1.12 ± 0.03 mmol g^–1^, corresponding to a 15 ± 3% overestimation. The observed discrepancy between the two methods used to calculate the adjusted guaiacyl content derived primarily from the effective ratio in syringyl and guaiacyl units in HKL, containing approximately 35% of aromatic rings of guaiacyl type. The adjusted phenol content, accounting only for monohydroxyl phenols, was calculated as 3.94 ± 0.04 mmol g^–1^. Thus, terminal catechol units contributed 12.1 ± 0.1% to the total phenolic content. Analytical-grade lignins from *Alnus glutinosa* were isolated via the Björkman method^24^ (AG MWL), enzymatic mild acidolysis^49,50^ (AG EMAL), and 1,4-dioxane acidolysis^18^ (AG organosolv). The content of terminal catechol units increased from 0.07 ± 0.01 mmol g^–1^ for AG MWL to 0.13 ± 0.02 mmol g^–1^ for AG organosolv. This increase was attributed to the different extraction conditions employed. Both mild acidolysis^49^ and the conventional organosolv process in 1,4-dioxane/water^51^ are expected to induce cleavage of aryl ether linkages to varying extents, thereby liberating previously etherified residues while preserving the original phenolic units. These results suggest that catechol units originally present in AG MWL survive the acidolysis conditions used for AG EMAL and AG organosolv, while the observed increase in their content may result from the liberation of additional catechol units through aryl ether cleavage.

Such hydrolytic cleavage may also involve benzodioxane structures. According to the mechanistic calculations,^52^ these structures are characterized by a lower dissociation enthalpy for α-O-3′ cleavage than that associated with the typical arylglycerol-β-aryl ether (β-O-4) linkage, suggesting that their acid-catalyzed cleavage may be favored under the conditions employed. For AG MWL and AG EMAL, no relevant differences in the apparent guaiacyl content were observed when comparing the use of eq 2 with that of eq 4, which accounts for the S/G ratio. Indeed, the respective S/G ratios (0.79 and 1.21) indicated that approximately 56 and 45% of the total aromatic rings were of guaiacyl type, approaching the assumption of equimolar contributions from 3,4-dihydroxyphenyl and 3,4-dihydroxy-5-methoxyphenyl terminal units used in eq 2. For AG MWL, the apparent guaiacyl content was 0.36 ± 0.02 mmol g^–1^ when equimolar contributions of the two terminal units were assumed, compared to 0.35 ± 0.02 mmol g^–1^ when the S/G ratio was considered. These values corresponded to guaiacyl overestimations of 16 ± 2 and 18 ± 3%, respectively. Similarly, for AG EMAL, the apparent guaiacyl contents were 0.54 ± 0.03 and 0.55 ± 0.03 mmol g^–1^, corresponding to overestimations of 16 ± 3 and 14 ± 2%, respectively. The higher S/G ratio of AG organosolv (1.65) resulted in a greater difference between the two approaches. The estimated guaiacyl overestimation was 17 ± 3% when the S/G ratio was considered, compared with 22 ± 3% under the assumption of equimolar contributions. Overall, terminal catechol units accounted for 19 ± 4% (AG EMAL) to 25 ± 4% (AG organosolv) of the total phenolic content for AG lignins.

Lastly, the application of the chelometric/UV–vis method to WS organosolv lignin revealed a content of 0.06 ± 0.01 mmol g^–1^ of terminal catechol units, accounting for 4.5 ± 0.7% of total phenolic hydroxyls. As observed in the cases of AG MWL and AG EMAL, no relevant difference in the apparent guaiacyl content was observed between eq 3 (0.99 ± 0.02 mmol g^–1^) and eq 4 (0.98 ± 0.02 mmol g^–1^), consistent with the S/G ratio of the sample, which indicates that approximately 59% of methoxylated rings were of the guaiacyl type.

## Conclusions

In this study, we demonstrate that catechol groups represent a previously overlooked source of ambiguity in conventional quantitative ^31^P NMR analyses of lignins. Using ad-hoc synthesized catechol-containing lignin model compounds, we show that TMDP-phosphitylated catechol hydroxyl groups substantially overlap with signals conventionally assigned to guaiacyl and syringyl phenolic hydroxyls. ^1^H–^13^C HSQC analysis provided only limited discrimination, owing to extensive signal overlap in the aromatic region. To address these limitations, we describe a complementary approach combining ^31^P NMR spectroscopy with a selective chelometric/UV–vis method based on differential spectroscopy. Although this approach provides an operational, semiquantitative estimate rather than an absolute quantification of terminal catechol units, it provides a practical means of (i) estimating the contribution of terminal 3,4-dihydroxyphenyl structures to the apparent guaiacyl hydroxyl content, thereby enabling calculation of an “adjusted guaiacyl content” under the structural assumptions described, and (ii) estimating the content of monohydroxyl phenolic moieties in lignin samples. Application of the combined methodology to a range of technical- and analytical-grade lignins revealed detectable catechol contents in all samples examined. The estimated catechol content accounted for approximately 6–27% of the apparent guaiacyl hydroxyl content and 5–24% of the total phenolic hydroxyl content.

The proposed approach appears to be particularly useful for the comparative analysis of closely related lignin samples, such as those derived from the same biomass and subjected to different processing conditions. Under routine analytical conditions, the chelometric/UV–vis method circumvents some of the limitations associated with conventional ^13^C NMR spectroscopy including extensive overlap between catechol and syringyl resonances, high sample amount requirements, and the long acquisition times of high-field instruments, which may not be readily available. Overall, while a definitive method for the absolute quantification of catechol structures in lignins remains to be established, our findings demonstrate that catechol groups can affect the interpretation of quantitative ^31^P NMR data for phenolic hydroxyl groups. This effect should therefore be considered when assessing the phenolic hydroxyl distribution of technical and chemically modified lignins.

## Supplementary Material



## Notes

Determination of average molar absorptivity and linear range of quantification; estimation of LOQ and LOD; error propagation in the measurements; calculation of the guaiacyl ratio from the S/G ratio; ^31^P NMR spectra of model compounds (Figs. S3–S7); ^1^H–^13^C HSQC NMR of model compounds (Figg. S8–S12); UV–vis spectra of protocatechuic and veratric acid with iron­(III) (Figure S13); UV–vis spectra of investigated lignins in the presence and absence of iron­(III) chloride (Figure S14); ^31^P NMR spectra of investigated lignins (Figs. S15–S23); ^1^H–^13^C HSQC NMR of investigated lignins (Figs. S24–S32); spiking test results (Table S1); and catechol content of investigated lignins, adjusted guaiacyl group content, and adjusted monohydroxyl phenolic content (Tables S2 and S3) (PDF)

## References

[1] Adler E. (1977). Lignin Chemistry—Past,
Present and Future. Wood Sci. Technol.

[2] Li Y., Shuai L., Kim H., Motagamwala A. H., Mobley J. K., Yue F., Tobimatsu Y., Havkin-Frenkel D., Chen F., Dixon R. A., Luterbacher J. S., Dumesic J. A., Ralph J. (2018). An “Ideal Lignin” Facilitates
Full Biomass Utilization. Sci. Adv.

[3] Ralph J., Lapierre C., Boerjan W. (2019). Lignin Structure
and Its Engineering. Curr. Opin. Biotechnol.

[4] Chen F., Tobimatsu Y., Havkin-Frenkel D., Dixon R. A., Ralph J. (2012). A Polymer
of Caffeyl Alcohol in Plant Seeds. Proc. Natl.
Acad. Sci. U. S. A.

[5] Ralph J., Lapierre C., Lu F., Marita J. M., Pilate G., Van Doorsselaere J., Boerjan W., Jouanin L. (2001). NMR Evidence for Benzodioxane
Structures Resulting from Incorporation of 5-Hydroxyconiferyl Alcohol
into Lignins of O-Methyltransferase-Deficient Poplars. J. Agric. Food Chem.

[6] Del
Río J. C., Martínez T., Gutiérrez A. (2007). Presence of
5-Hydroxyguaiacyl Units as Native Lignin Constituents in Plants as
Seen by Py-GC/MS. J. Anal. Appl. Pyrolysis.

[7] Gierer J. (1980). Chemical Aspects
of Kraft Pulping. Wood Sci.Technol.

[8] Gierer J. (1985). Chemistry
of Delignification. Wood Sci. Technol.

[9] Gierer J. (1986). Chemistry
of Delignification. Wood Sci.Technol.

[10] Gratzl, J. S. ; Chen, C.-L. Chemistry of Pulping: Lignin Reactions. In Lignin: historical, Biological, and Materials Perspectives; American Chemical Society, 1999; Vol. 742, pp. 392–421. DOI: 10.1021/bk-2000-0742.ch020

[11] Pajer N., Crestini C., Argyropoulos D. S. (2026). Spiers
Memorial Lecture: Organic,
Physical & Polymer Aspects Pivotal in Lignin Valorization. Faraday Discuss.

[12] Ishihara T., Miyazaki M. (1974). Demethylation of Lignin and Lignin Models by Fungal
Laccase. Mokuzai Gakkaishi.

[13] Ishihara, T. The Role of Laccase in Lignin Biodegradation. In Lignin Biodegradation: microbiology, Chemistry, and Potential Applications; CRC Press, 1980

[14] Harlington A. C., Das T., Shearwin K. E., Bell S. G., Whelan F. (2025). Structural Insights
into S-Lignin O-Demethylation via a Rare Class of Heme Peroxygenase
Enzymes. Nat. Commun.

[15] Zhao Y., Dong R., Wang G., Li W., Sui W., Jia H., Si C. (2025). Lignin-Based Metal
Chelate Fertilizers: Preparation,
Properties and Applications. Int. J. Biol. Macromol.

[16] Liu Q., Kawai T., Inukai Y., Aoki D., Feng Z., Xiao Y., Fukushima K., Lin X., Shi W., Busch W., Matsushita Y., Li B. (2023). A Lignin-Derived Material
Improves Plant Nutrient Bioavailability and Growth through Its Metal
Chelating Capacity. Nat. Commun.

[17] Robert, D. Carbon-13 Nuclear Magnetic Resonance Spectrometry. In Methods in Lignin Chemistry, Lin, S. Y. ; Dence, C. W. Eds.; Springer: Berlin, Heidelberg, 1992; pp. 250–273. DOI: 10.1007/978-3-642-74065-7_18.

[18] Pajer N., Crestini C. (2025). Quantification of Carbonyl Groups
in Lignin: ^31^P NMR after Reduction. ACS Sustainable Chem.
Eng.

[19] Kringstad K. P., Mörck R. (1983). ^13^C-NMR Spectra of Kraft
Lignins. Holzforschung.

[20] Argyropoulos D. S., Pajer N., Crestini C. (2021). Quantitative
31P NMR Analysis of
Lignins and Tannins. J. Visual. Exper.

[21] Pajer N., Danelon U., Crestini C. (2025). NMR Spectroscopy:
An Invaluable Tool
to Identify Lignin Structural Modifications Induced by Oxidative Enzymes. Methods Enzymol.

[22] Quideau S., Ralph J. (1992). Facile Large-Scale
Synthesis of Coniferyl, Sinapyl, and p-Coumaryl
Alcohol. J. Agric. Food Chem.

[23] Nousiainen, P. A. ; Mikkilä, J. ; Kontro, J. ; Tiimonen, H. ; Häggmann, H. ; Karhunen, P. ; Sipilä, J. Lignin Catechol Structures And Their Structural Elucidation In COMT Deficient Birches.

[24] Bjorkman A. (1956). Studies on
Finely Divided Wood. Part 1. Extraction of Lignin with Neutral Solvents. Svensk Pappersit.

[25] Falkehag, S. I. ; Marton, J. ; Adler, E. Chromophores in Kraft Lignin. In Lignin Structure and Reactions; Advances in Chemistry; American Chemical Society, 1966; Vol. 59, pp. 75–89. DOI: 10.1021/ba-1966-0059.ch007

[26] Adler E., Brunow G., Lundquist K. (1987). Investigation
of the Acid-Catalysed
Alkylation of Lignins by Means of NMR Spectroscopic Methods. Holzforschung.

[27] Granata A., Argyropoulos D. S. (1995). 2-Chloro
4,4,5,5-Tetramethyl-1,3,2-Dioxaphospholane,
a Reagent for the Accurate Determination of the Uncondensed and Condensed
Phenolic Moieties in Lignins. J. Agric. Food
Chem.

[28] Argyropoulos D. S. (1995). ^31^P NMR in Wood Chemistry: A Review of Recent Progress. Res. Chem. Intermed.

[29] Wroblewski A. E., Lensink C., Markuszewski R., Verkade J. G. (1988). Phosphorus-31 NMR
Spectroscopic Analysis of Coal Pyrolysis Condensates and Extracts
for Heteroatom Functionalities Possessing Labile Hydrogen. Energy Fuels.

[30] Meng X., Parikh A., Seemala B., Kumar R., Pu Y., Wyman C. E., Cai C. M., Ragauskas A. J. (2019). Characterization
of Fractional Cuts of Co-Solvent Enhanced Lignocellulosic Fractionation
Lignin Isolated by Sequential Precipitation. Bioresour. Technol.

[31] Uddin N., Li X., Ullah M. W., Sethupathy S., Chen F., Yang B., Zhu D. (2024). Unlocking
the Potential of Catechyl Lignin: Molecular Regulation
of Biosynthesis, Structural Organization, And Valorization. ACS Sustainable Chem. Eng.

[32] Wang S., Shen Q., Su S., Lin J., Song G. (2022). The Temptation
from Homogeneous Linear Catechyl Lignin. Trends
Chem.

[33] Choi C.-Y., Lossada F., Walter K., Fleck-Kunde T., Behrens S., Meinelt T., Falkenhagen J., Hiller M., Oschkinat H., Dallmann A., Taden A., Börner H. G. (2024). Organic Transformation of Lignin into Mussel-Inspired
Glues: Next-Generation 2K Adhesive for Setting Corals under Saltwater. Green Chem.

[34] Goldschmid O. (1954). Determination
of Phenolic Hydroxyl Content of Lignin Preparations by Ultraviolet
Spectrophotometry. Anal. Chem.

[35] Lai, Y.-Z. Determination of Phenolic Hydroxyl Groups. In Methods in Lignin Chemistry, Lin, S. Y. ; Dence, C. W. Eds.; Springer: Berlin, Heidelberg, 1992; pp. 423–434. DOI: 10.1007/978-3-642-74065-7_28

[36] Lin, S. Y. Ultraviolet Spectrophotometry. In Methods in Lignin Chemistry, Lin, S. Y. ; Dence, C. W. Eds.; Springer: Berlin, Heidelberg, 1992; pp. 217–232. DOI: 10.1007/978-3-642-74065-7_15

[37] Marton, J. ; Marton, T. ; Falkehag, S. I. ; Adler, E. Alkali-Catalyzed Reactions of Formaldehyde with Lignins. In Lignin Structure and Reactions; American Chemical Society, 1966; Vol. 59, pp. 125–144. DOI: 10.1021/ba-1966-0059.ch010

[38] Aulin-Erdtman G., Hegbom L. (1958). Spectrographic Contributions to Lignin
Chemistry. VIII.
Studies on Brauńs “Native Lignins” from Coniferous
Wood. Sven. Papperstidn.

[39] Imsgard F., Ingemar S., Knut F. (1971). On Possible Chromophoric
Structures
in Spruce Wood. Tappi.

[40] Ishibashi M., Shigematsu T., Yamamoto Y., Tabushi M., Kitayama M. (1957). Ultraviolet
Spectrophotometric Determination of Iron (III) as Acetato-Complex. Bull. Inst. Chem. Res..

[41] Vukosav P., Mlakar M., Tomišić V. (2012). Revision of
Iron­(III)–Citrate
Speciation in Aqueous Solution. Voltammetric and Spectrophotometric
Studies. Anal. Chim. Acta.

[42] Pozdnyakov I. P., Kolomeets A. V., Plyusnin V. F., Melnikov A. A., Kompanets V. O., Chekalin S. V., Tkachenko N., Lemmetyinen H. (2012). Photophysics
of Fe­(III)–Tartrate and Fe­(III)–Citrate Complexes in
Aqueous Solutions. Chem. Phys. Lett.

[43] Nurchi V. M., Pivetta T., Lachowicz J. I., Crisponi G. (2009). Effect of Substituents
on Complex Stability Aimed at Designing New Iron­(III) and Aluminum­(III)
Chelators. J. Inorg. Biochem.

[44] Ciavatta L., De Tommaso G., Iuliano M. (2001). On the Formation of Iron (III) Hydroxo
Acetate Complexes. Ann. Chim.

[45] Xia Z., Akim L. G., Argyropoulos D. S. (2001). Quantitative ^13^C NMR Analysis
of Lignins with Internal Standards. J. Agric.
Food Chem.

[46] Capanema E. A., Balakshin M. Y., Kadla J. F. (2004). A Comprehensive Approach for Quantitative
Lignin Characterization by NMR Spectroscopy. J. Agric. Food Chem.

[47] Balakshin M., Capanema E. (2015). On the Quantification
of Lignin Hydroxyl Groups With ^31^P and ^13^C NMR
Spectroscopy. J. Wood Chem. Technol.

[48] Argyropoulos D. S., Bolker H. I. (1987). Condensation of
Lignin in Dioxane-Water-HCl. J. Wood Chem. Technol.

[49] Wu S., Argyropoulos D. S. (2003). An Improved
Method for Isolating Lignin in High Yield
and Purity. J. Pulp Pap. Sci.

[50] Guerra A., Mendonça R., Ferraz A., Lu F., Ralph J. (2004). Structural
Characterization of Lignin during Pinus Taeda Wood Treatment with
Ceriporiopsis Subvermispora. Appl. Environ.
Microbiol.

[51] Pepper J. M., Baylis P. E. T., Adler E. (1959). The Isolation
and Properties of Lignins
Obtained by the Acidolysis of Spruce and Aspen Woods in Dioxane–Water
Medium. Can. J. Chem.

[52] Elder T., Berstis L., Beckham G. T., Crowley M. F. (2016). Coupling and Reactions
of 5-Hydroxyconiferyl Alcohol in Lignin Formation. J. Agric. Food Chem.

