# Supplementary material for: Reconsidering Catechol Contributions in Quantitative 31P NMR Analyses of Lignins
Source: ACS Omega. 2026 Oct 28;11(44):69324–35. doi: 10.1021/acsomega.6c09845 (PMC13756652; doi:10.1021/acsomega.6c09845)
Supplement: Supplementary file 1 [file ao6c09845_si_001.pdf]

Supporting Information

Reconsidering Catechol Contributions in Quantitative ^{31}P NMR Analyses of Lignins

Nicolò Pajer,[a]* Paula A. Nousiainen[a], and Monika Österberg[a]

[a] Department of Bioproducts and Biosystems, School of Chemical Engineering, Aalto University, Vuorimiehentie 1, 02150 Espoo, Finland

Methods

Determination of average molar absorptivity and linear range of quantification

Estimation of LOQ and LOD

Error propagation in the measurements

Calculation of guaiacyl ratio from S/G ratio

Figures and Tables

Figure S1. Plot of the differential absorbance at 572 nm between equimolar solutions of iron (III) *p*-methyl-catecholate.

Figure S2. Linear range of quantification at 572 nm.

Figure S3. ^{31}P NMR spectrum of *p*-methyl-catechol in pyridine/ $CDCl_3$.

Figure S4. ^{31}P NMR spectrum of Caffeoyl alcohol in pyridine/ $CDCl_3$.

Figure S5. ^{31}P NMR spectrum of Caffeic acid in pyridine/ $CDCl_3$.

Figure S6. ^{31}P NMR spectrum of Catechyl-(β-O-4´) in pyridine/ $CDCl_3$.

Figure S7. ^{31}P NMR spectrum of 5-methyl-catechyl-(β-O-4´) in pyridine/ $CDCl_3$.

Figure S8. *p*-methyl-catechol. ^{1}H-^{13}C HSQC spectrum in DMSO-d_6.

Figure S9. Caffeoyl alcohol. ^{1}H-^{13}C HSQC spectrum in DMSO-d_6.

Figure S10. Caffeic acid. ^{1}H-^{13}C HSQC spectrum in DMSO-d_6.

Figure S11. 5-methyl-catechyl-(β-O-4´). ^{1}H-^{13}C HSQC spectrum in DMSO-d_6.

Figure S12. Benzo-dioxane. ^{1}H-^{13}C HSQC spectrum in DMSO-d_6.

Figure S13. UV-vis spectra of protocatechuic and veratric acid with iron (III).

Figure S14. UV-vis spectra of investigated lignins in presence and absence of iron (III) chloride.

Figure S15. ^{31}P NMR spectrum of SW kraft 1 in pyridine/ $CDCl_3$.

Figure S16. ^{31}P NMR spectrum of SW kraft 2 in pyridine/ $CDCl_3$.

Figure S17. ^{31}P NMR spectrum of HW kraft in pyridine/ $CDCl_3$.

Figure S18. ^{31}P NMR spectrum of PE MWL in pyridine/ $CDCl_3$.

Figure S19. ^{31}P NMR spectrum of PE organosolv in pyridine/ $CDCl_3$.

Figure S20. ^{31}P NMR spectrum of AG MWL in pyridine/ $CDCl_3$.

Figure S21. ^{31}P NMR spectrum of AG EMAL in pyridine/ $CDCl_3$.

Figure S22. ^{31}P NMR spectrum of AG organosolv in pyridine/ $CDCl_3$.

Figure S23. ^{31}P NMR spectrum of WS organosolv in pyridine/ $CDCl_3$.

Figure S24. ^{1}H-^{13}C HSQC spectrum of SW kraft, 1 in DMSO-d_6.

Figure S25. ^{1}H-^{13}C HSQC spectrum of SW kraft 2 in DMSO-d_6.

Figure S26. ^{1}H-^{13}C HSQC spectrum of HW kraft in DMSO-d_6.

Figure S27. ^{1}H-^{13}C HSQC spectrum of PE MWL in DMSO-d_6.

Figure S28. ^{1}H-^{13}C HSQC spectrum of PE organosolv in DMSO-d_6.

Figure S29. ^{1}H-^{13}C HSQC spectrum of AG MWL in DMSO-d_6.

Figure S30. ^{1}H-^{13}C HSQC spectrum of AG EMAL in DMSO-d_6.

Figure S31. ^{1}H-^{13}C HSQC spectrum of AG organosolv in DMSO-d_6.

Figure S32. ^{1}H-^{13}C HSQC spectrum of WS organosolv in DMSO-d_6.

Table S1. Catechyl-adjusted guaiacyl contents for different technical and analytical grade lignins.

Table S2. Catechyl-adjusted content of phenolic hydroxyl (mono-hydroxyl phenols content) for different technical and analytical grade lignins.

Determination of average molar absorptivity and linear range of quantification

The average molar absorptivity coefficient (ε) (1050 L·mol^{-1}·cm^{-1}) used in the present work was estimated by calculating the average molar absorptivity coefficient of the iron (III) catecholate complexes formed with *o*-catechol, *p*-methyl-catechol, protocatechuic acid, and protocatechuic aldehyde.

Before estimating the molar absorptivity coefficients for the iron (III) catecholate complexes of the different models, the range of linearity between the concentration of the ligand and the absorbance of the solution was determined using *p*-methyl-catechol as representative of all catechol models. Specifically, solutions of p-methyl-catechol, both in presence and absence of iron (III), with concentrations ranging from 0.015 mM to 3.10 mM, were prepared according to the procedure described for the preparation of the lignin samples used in the analytical protocol. The spectra of the different solutions were recorded using 2-methoxy-ethanol/DMSO as reference. The differential absorbance at 572 nm (ΔA_{572}) was calculated as the difference between the absorbance of the iron (III) catecholate ($A_{model\text{-}Fe,\,572}$) and that of the corresponding blank solution ($A_{model,\,572}$):

$$\Delta A_{572} = A_{model-Fe,\,572} - A_{model,\,572}$$

The resulting ΔA_{572} values were plotted against the concentration of the solution in *p*-methyl-catechol as shown in Figure S13. Assuming the quantitative formation of the iron (III) catecholate complex under the experimental conditions described, a linear correlation between catecholate complex and absorbance was found at concentrations ranging from 0.015 mM to 0.40 mM (R^2 = 0.9969, Figure S14).

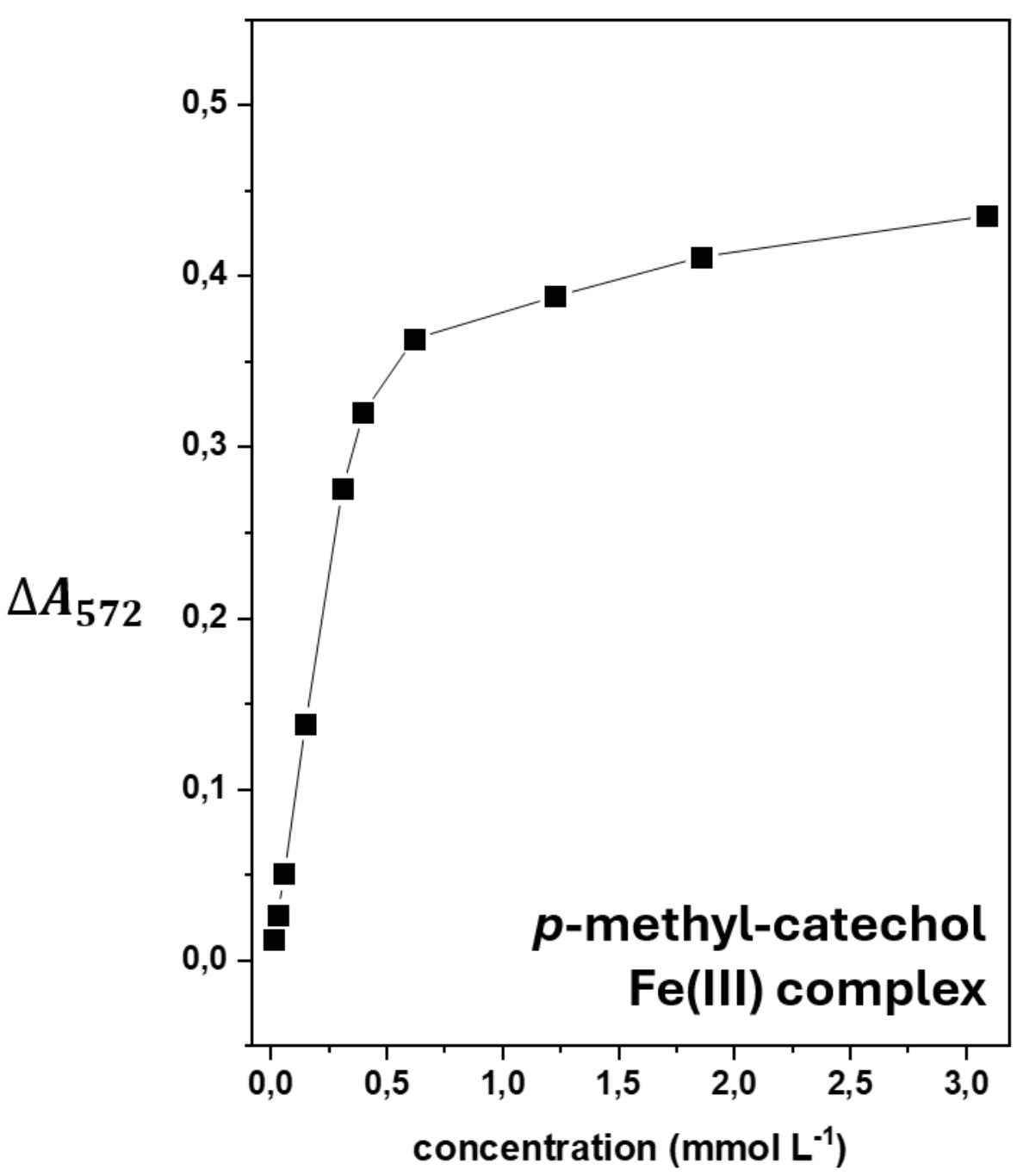

Figure S1. Plot of the differential absorbance at 572 nm between equimolar solutions of iron (III) *p*-methyl-catecholate.

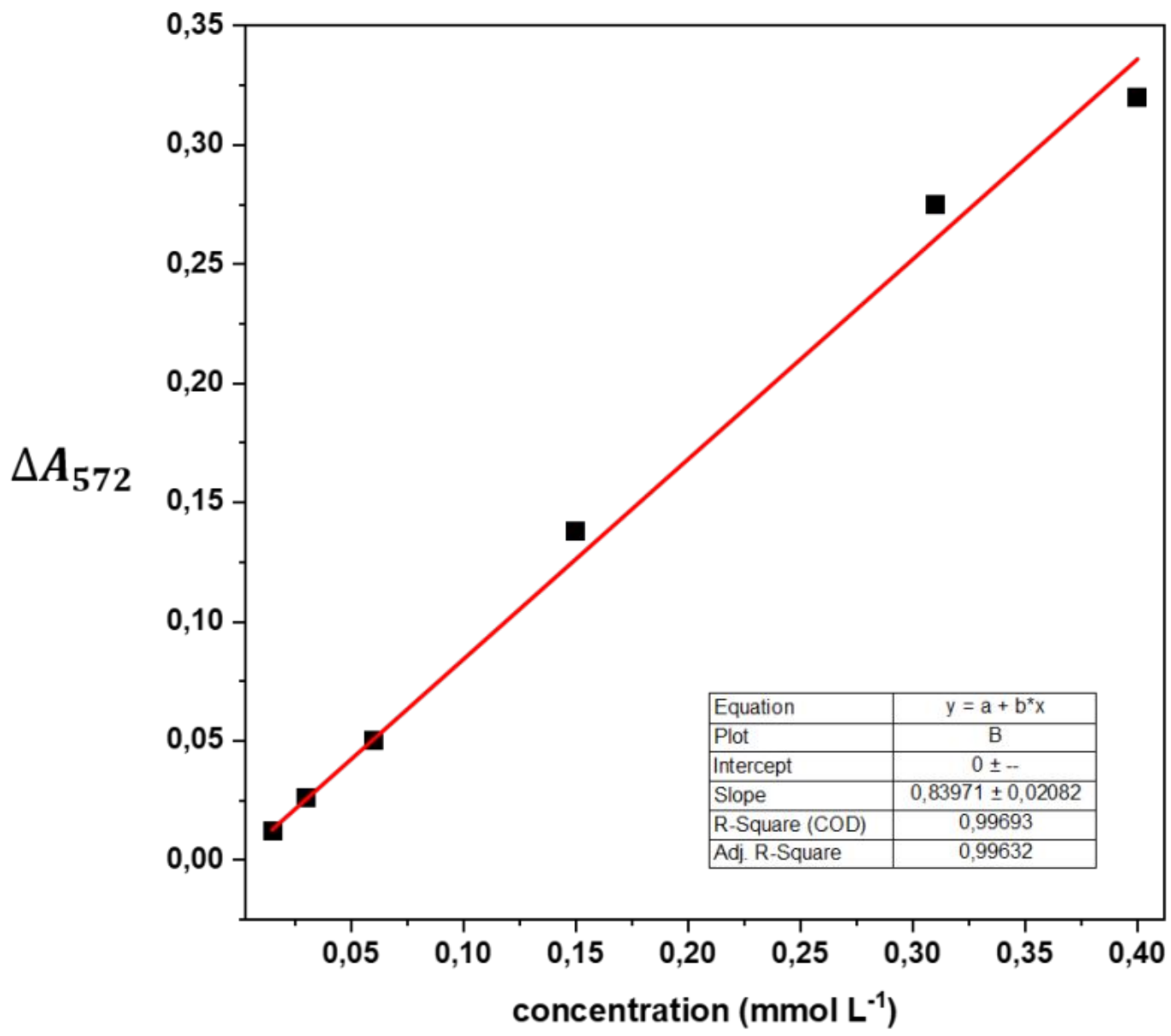

Figure S2. Linear range of quantification at 572 nm.

The differential absorbance of solutions of iron (III) catecholate complexes for the other models, o-catechol, protocatechuic aldehyde, and protocatechuic acid, was analysed at the same concentrations used to define the linearity range for the iron (III) catecholate complexes of *p*-methyl-catechol. Linear fitting demonstrated similarly good linearity ($R^2 > 0.9969$) of the differential absorbances as a function of concentration, as indicated by the R^2 values, to that observed for *p*-methyl-catechol concentration range:

	**R^2 (0.015 < C (mM) < 0.40)**
***o*-catechol**	0.9993
***p*-methyl-catechol (reference)**	0.9969
**protocatechuic acid**	0.9986
**protocatechuic aldehyde**	0.9995

The **molar absorptivities** for the different model compounds were calculated from the differential absorbance (ΔA_{572}) measured for iron (III)-catecholate solution for 0.0308 mM solutions. The molar absorptivity coefficient (ε_{572}) was calculated according to the Lambert-Beer law:

$$\Delta A_{572} = \varepsilon_{572} \cdot C \cdot l \rightarrow \varepsilon_{572} = \frac{\Delta A_{572}}{p \cdot C \cdot l}$$

Where p is the purity of the catechol compound. Results are summarised in the following table:

	**ε_{572} ($L \cdot mol^{-1} \cdot cm^{-1}$)**
***o*-catechol**	840
***p*-methyl-catechol**	920
**protocatechuic acid**	1110
**protocatechuic aldehyde**	1320
**average**	1050 ± 240

Estimation of LOQ and LOD

The limit of quantification in absorbance ($LOQ_{absorbance}$) was calculated based on the standard deviation (σ_{blank}) of the absorbance of ten measurements of the absorbance at 572 nm of different aliquots of a 0.0308 mM blank solution of p-methyl-catechol versus a reference solution composed of 2-methoxy-ethanol/dimethyl sulphoxide by applying the following equation:

$$LOQ_{absorbance} = 10 \cdot \sigma_{blank}$$

#	$A_{model,\ 572}$
**1**	0.007
**2**	0.007
**3**	0.007
**4**	0.007
**5**	0.007
**6**	0.007
**7**	0.006
**8**	0.006
**9**	0.006
**10**	0.005
**σ_{blank}**	0.000671

the standard deviation (σ_{blank}) was calculated as:

$$\sigma_{blank} = \sqrt{\frac{\sum(x_i - \bar{x})^2}{N-1}}$$

where x_i is the i-th value measured, x represents the average of the series, and N is the number of measurements (10).

Consequently:

$$LOQ_{absorbance} \quad 0.00671$$

The effective LOQ was determined from the Lambert-Beer equation, replacing the absorbance with $LOQ_{absorbance}$, thus:

$$LOQ = \frac{LOQ_{absorbance}}{\varepsilon \cdot l} = 0.00729\ mM$$

The value of ε used was the one calculated for *p*-methyl-catechol (920 $L \cdot mol^{-1} \cdot cm^{-1}$).
The limit of detectability (LOD) was determined from the LOQ according to the following equation:

$$LOD = \frac{LOQ}{10} \cdot 3.3 = 0.00240\ mM$$

Error propagation in the measurements

The apparent guaiacyl content, determined by ^{31}P NMR spectroscopy, and the catechol-unit content, determined by the chelometric/UV-vis method, were calculated as the average of three independent measurements. The associated absolute error (e) was estimated as half of the difference between the maximum (x_{max}) and minimum (x_{min}) values obtained for each sample, according to:

$$e = \frac{x_{max} - x_{min}}{2}$$

Thus:

$$(\bar{x} \pm e)$$

The corrected guaiacyl content was calculated from the difference between the apparent guaiacyl content and the catechol-unit content. As the apparent guaiacyl content and the catechol content were determined independently, using different analytical methods, the uncertainty associated with the corrected guaiacyl content was estimated using the root-sum-of-squares (RSS) method, based on the absolute errors associated with the apparent guaiacyl (e_G) and catechol (e_C) contents, assuming as independent the uncertainities:

$$RSS = \sqrt{{e_G}^2 + {e_C}^2}$$

When the estimated catechol-unit content was multiplied by a factor, such as in the case of Equations 2, 3, or 4, the corresponding absolute error (e_C) was multiplied by the same factor before propagation. The same approach was used for calculating the error bar in the corrected content of mono-hydroxy phenolics.

The uncertainty associated with the percentage of catechol groups, expressed relative to the apparent guaiacyl content, was calculated according to the standard rules for error propagation in ratios. The percentage was calculated as:

$$\%_{catechol\ per\ guaiacyl\ by\ 31P\ NMR} = \frac{\overline{catechol}_{chelometric/UV-vis}}{\overline{apparent\ guaiacyl}} \cdot 100$$

The relative errors associated with the catechol ($r_{e,C}$) and apparent guaiacyl ($r_{e,G}$) contents were calculated by dividing the corresponding absolute errors by their respective average values:

$$r_{e,C} = \frac{e_C}{\overline{catechol}_{chelometric/UV-vis}}$$

$$r_{e,G} = \frac{e_G}{\overline{apparent\ guaiacyl}}$$

The relative error associated with the percentage of catechol groups relative to apparent guaiacyl groups ($r_{e,\%}$) was then calculated as the sum of the two relative errors:

$$r_{e,\%} = \sqrt{{r_{e,C}}^2 + {r_{e,G}}^2}$$

Finally, the absolute error ($e_{\%}$) associated with the percentage was calculated by multiplying the percentage value by $r_{e,\%}$:

$$e_{\%} = \%_{catechol\,per\,guaiacyl\,by\,31P\,NMR} \cdot r_{e,\%}$$

The results were therefore reported as:

$$(\%_{catechol\,per\,guaiacyl\,by\,31P\,NMR} \pm e_{\%})\%$$

Calculation of guaiacyl ratio from S/G ratio

S/G is the ratio between syringyl (S) and guaiacyl (G) rings in a lignin sample. The relative proportion of syringyl units (r_S) among the S and G units can therefore be expressed as:

$$r_S = \frac{S}{S+G} = \frac{\left(\frac{S}{G}\right)}{\left(1+\frac{S}{G}\right)}$$

If the relative proportions of syringyl (r_S) and guaiacyl (r_G) units are normalised to their combined amount, then:

$$r_S + r_G = 1$$

And consequently:

$$r_G = 1 - r_S$$

The present normalisation refers specifically to the S+G fraction. In grass lignins, which contain also p-hydroxyphenyl units, $r_S + r_G = 1$ when the ratios are normalised to S + G, as in the case when the S/G ratio is used directly.

Selected ^{31}P NMR spectra for model compounds

^{31}P NMR analyses of lignin model compounds were run on samples prepared with 8-10 mg aliquots of model compounds according to the protocol previously reported.[1,2] Internal standard at δ_P=151.8 ppm. Spectra have been referenced to TMDP-derivatised residual water signal at 132.2 ppm.

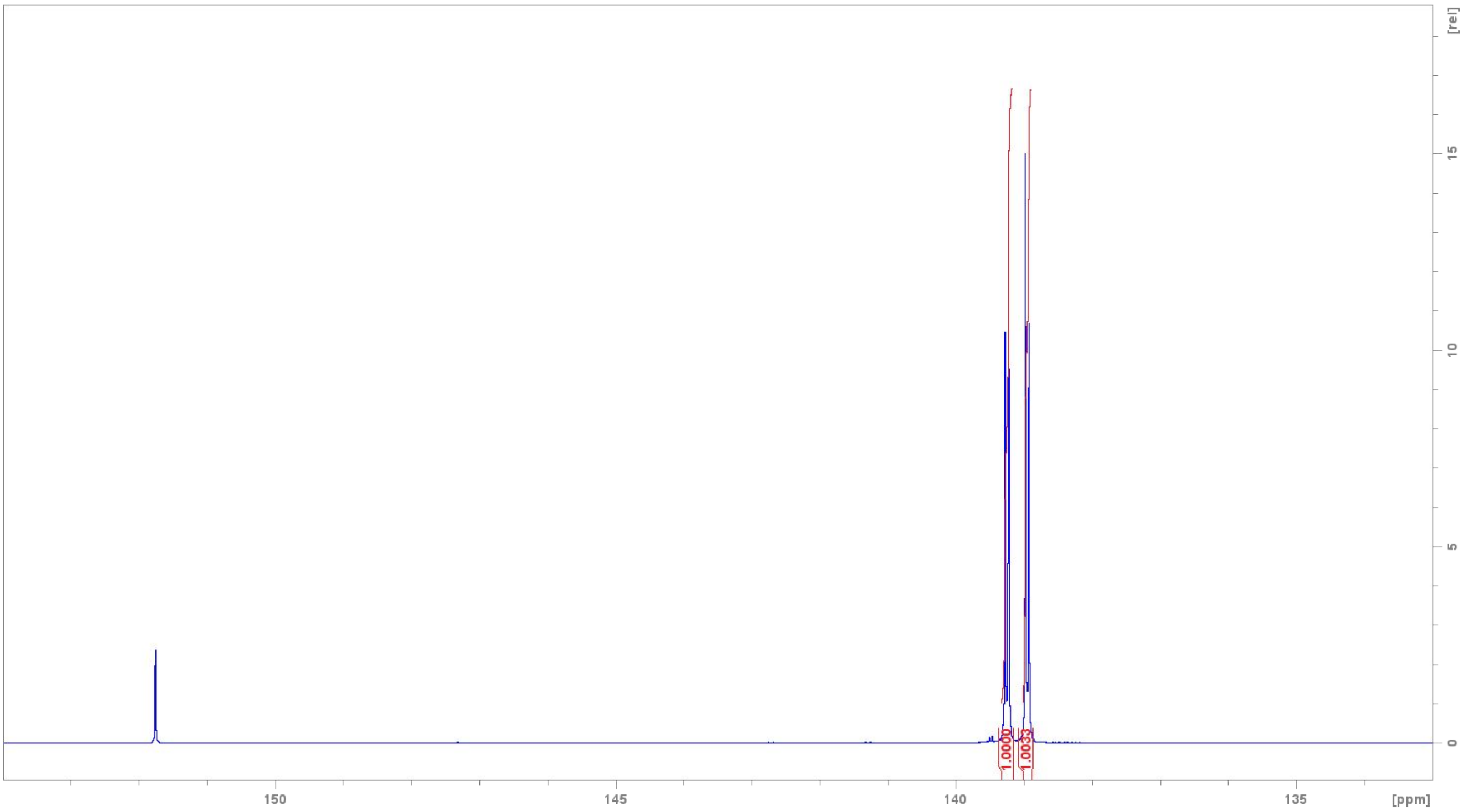

Figure S3. ^{31}P NMR spectrum of *p*-methyl-catechol in pyridine/$CDCl_3$.

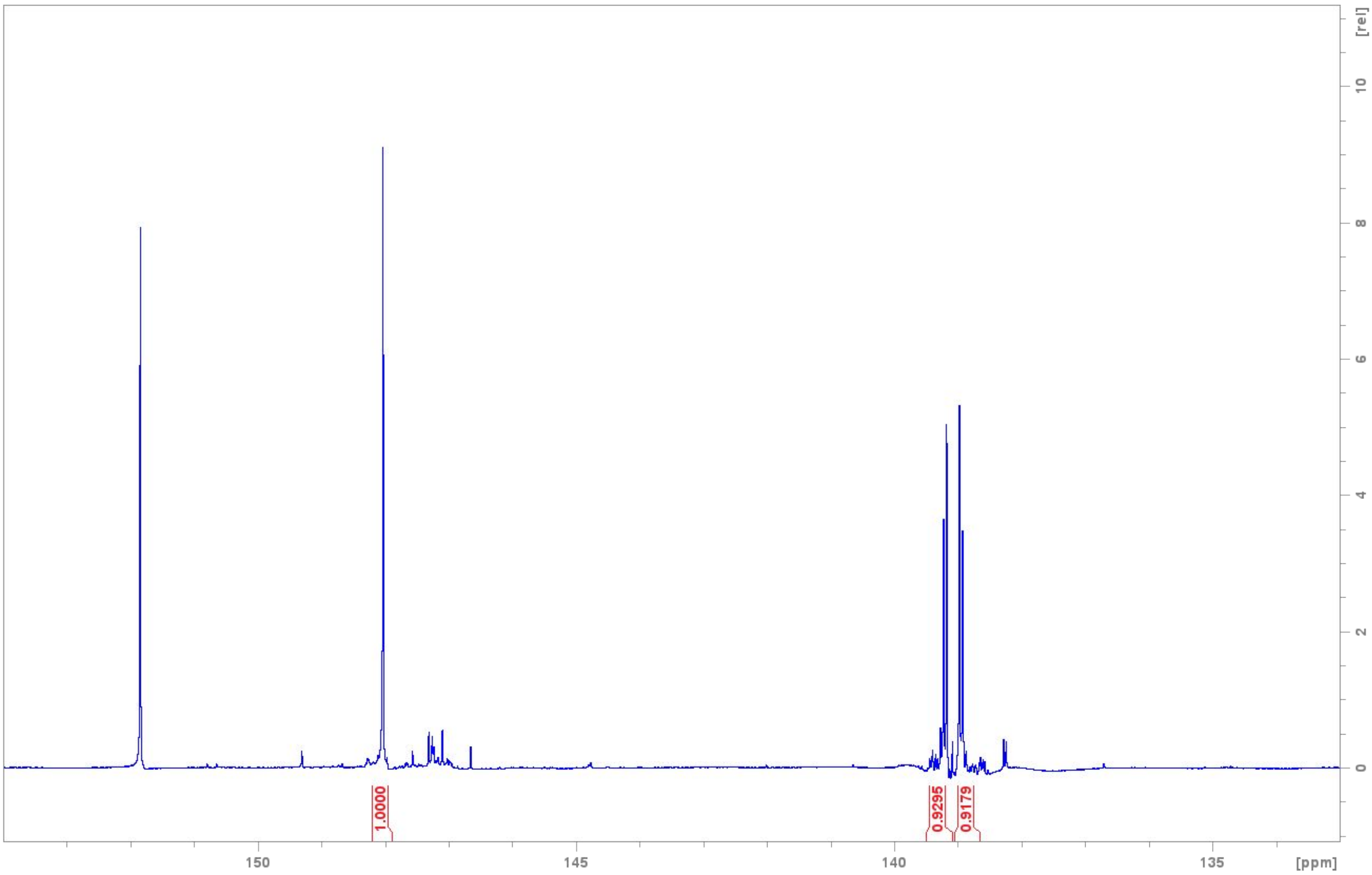

Figure S4. ${}^{31}P$ NMR spectrum of Caffeoyl alcohol.

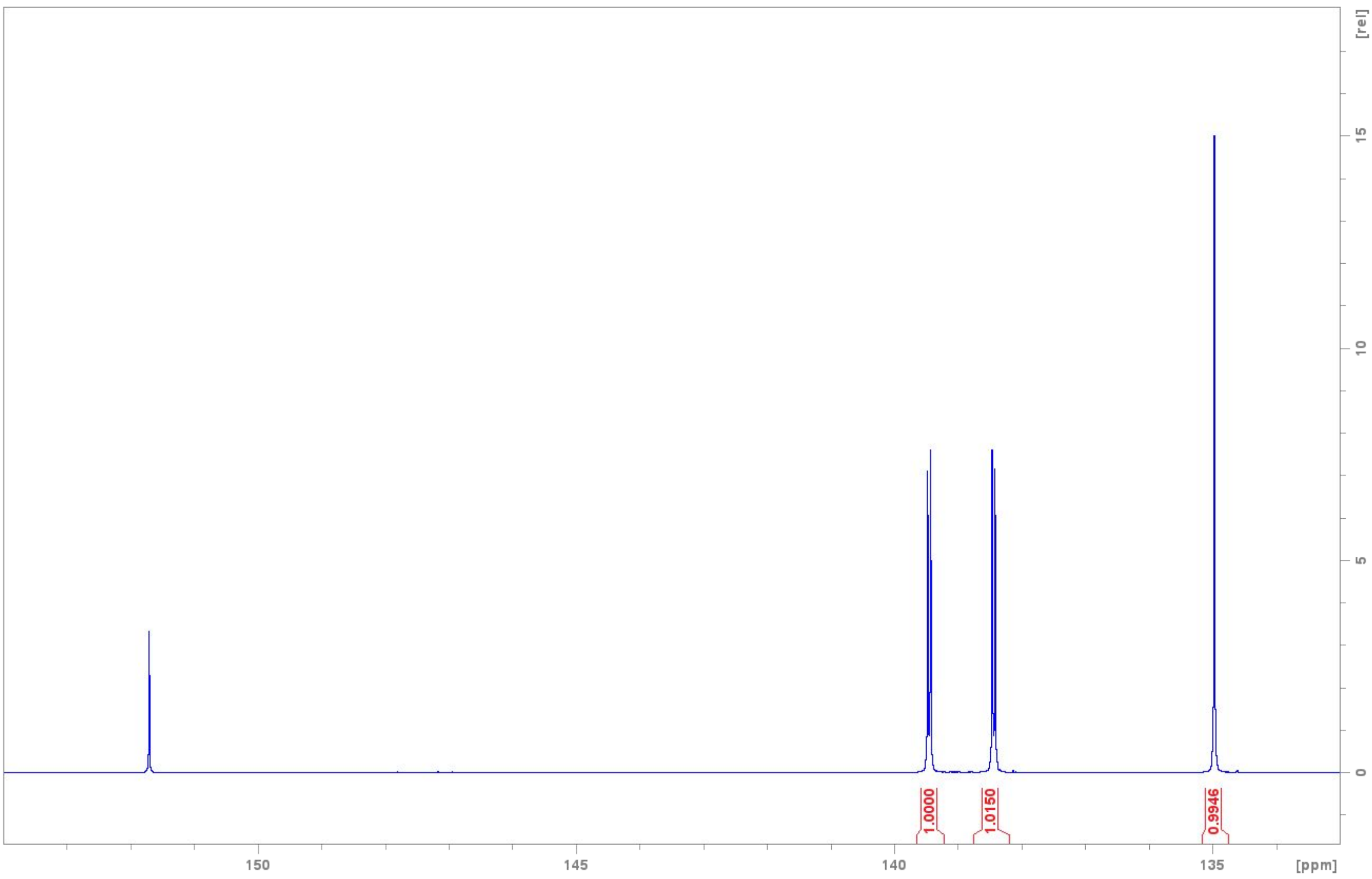

Figure S5. ${}^{31}P$ NMR spectrum of Caffeic acid.

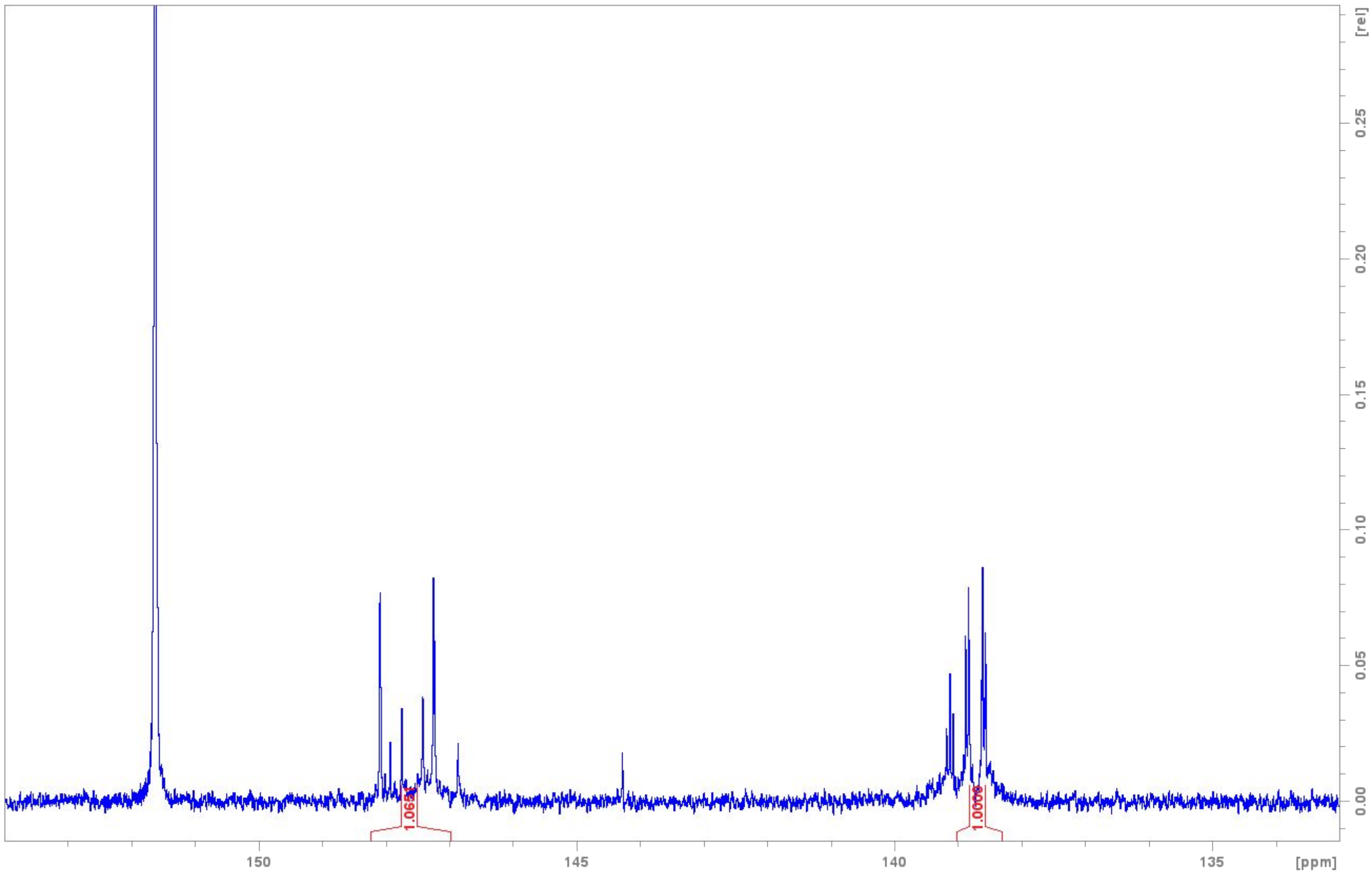

Figure S6. ^{31}P NMR spectrum of Catechyl-(β-O-4´).

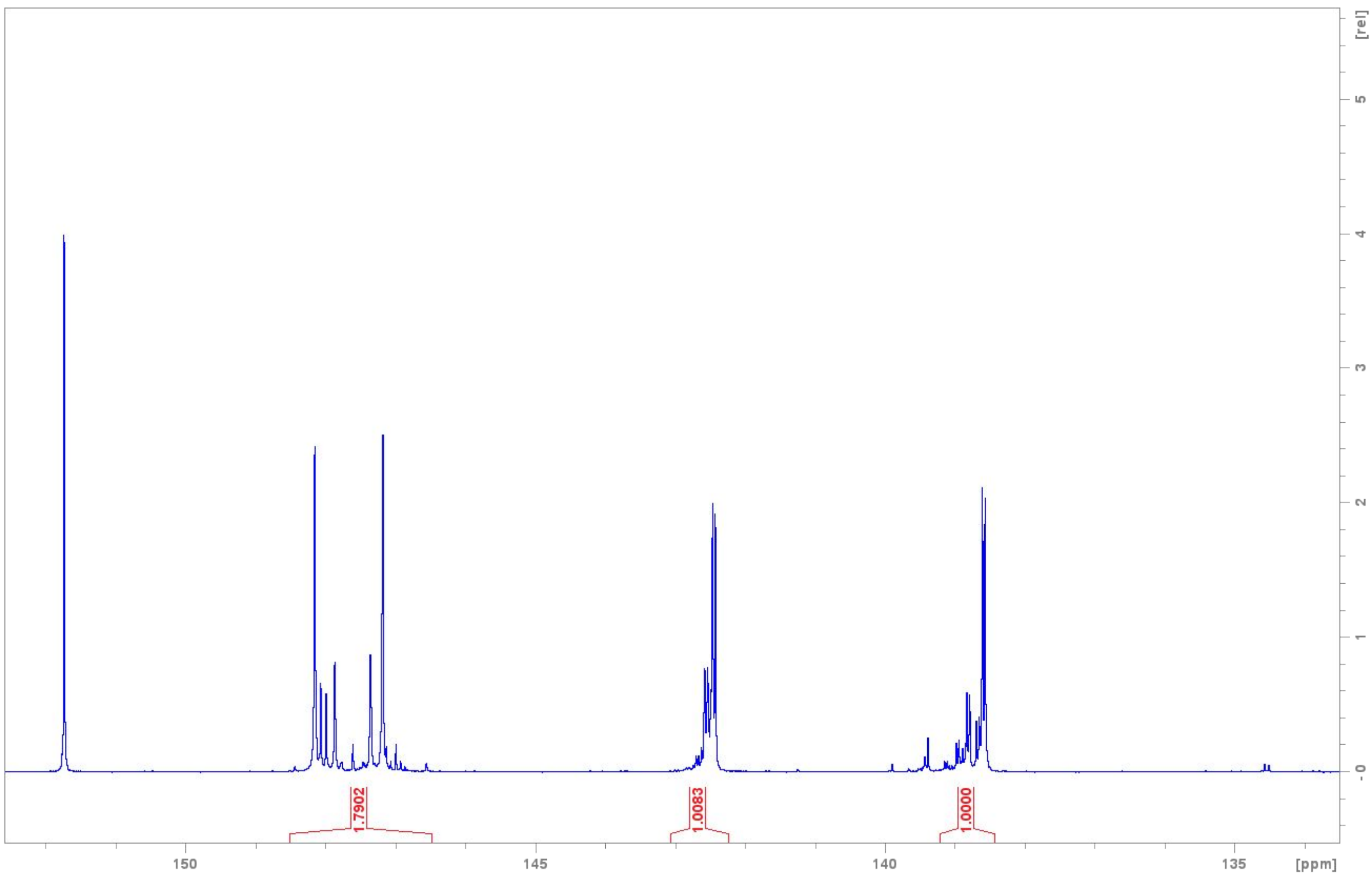

Figure S7. ^{31}P NMR spectrum of 5-methyl-catechyl-(β-O-4´).

Selected ^{1}H-^{13}C HSQC spectra

^{1}H-^{13}C HSQC analyses of lignin model compounds were run on samples prepared with 8-10 mg aliquots of model compounds dissolved in DMSO-d6 according to the protocol previously described. [1]

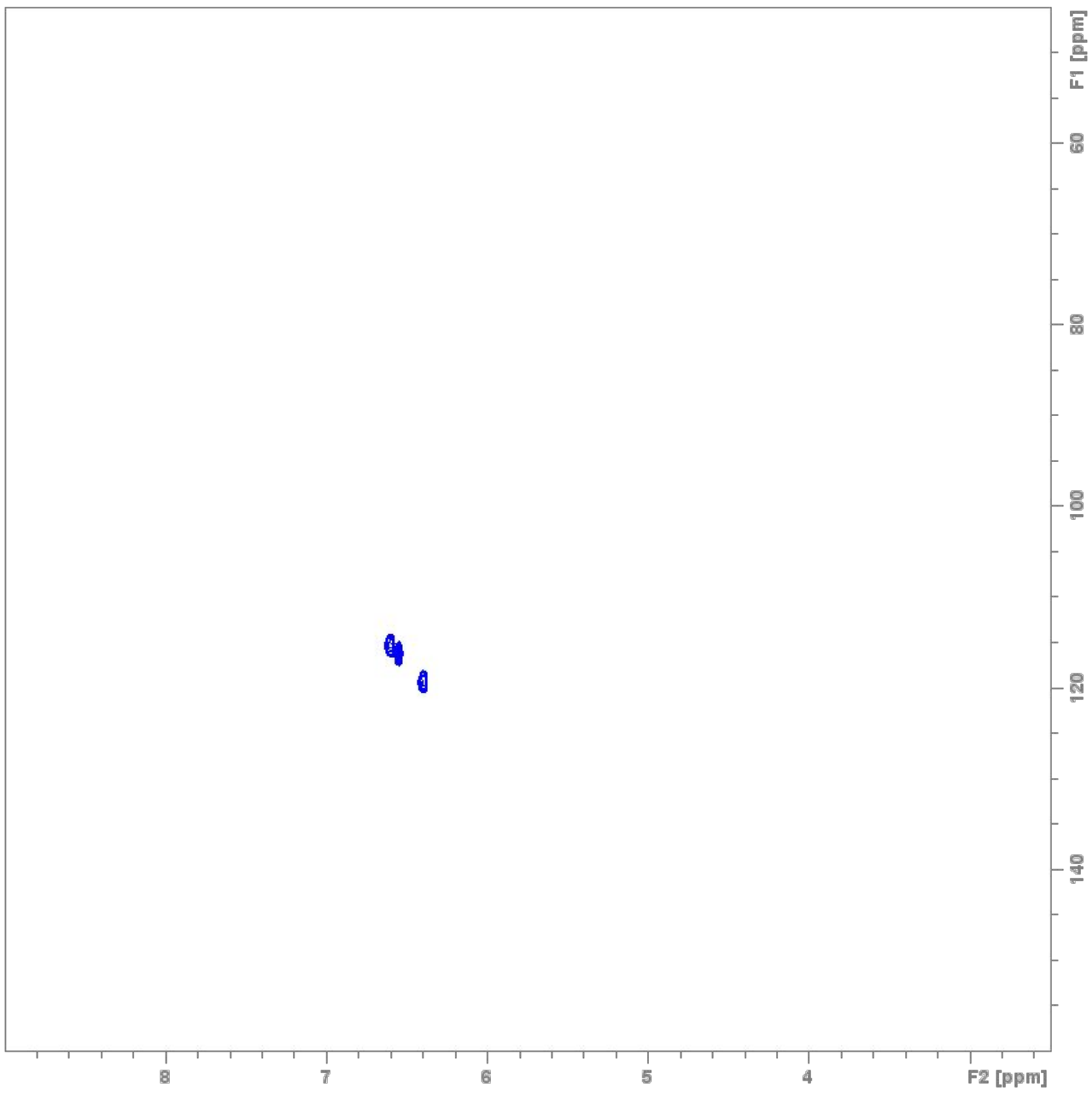

Figure S8. *p*-methyl-catechol. ^{1}H-^{13}C HSQC spectrum in DMSO-d_6.

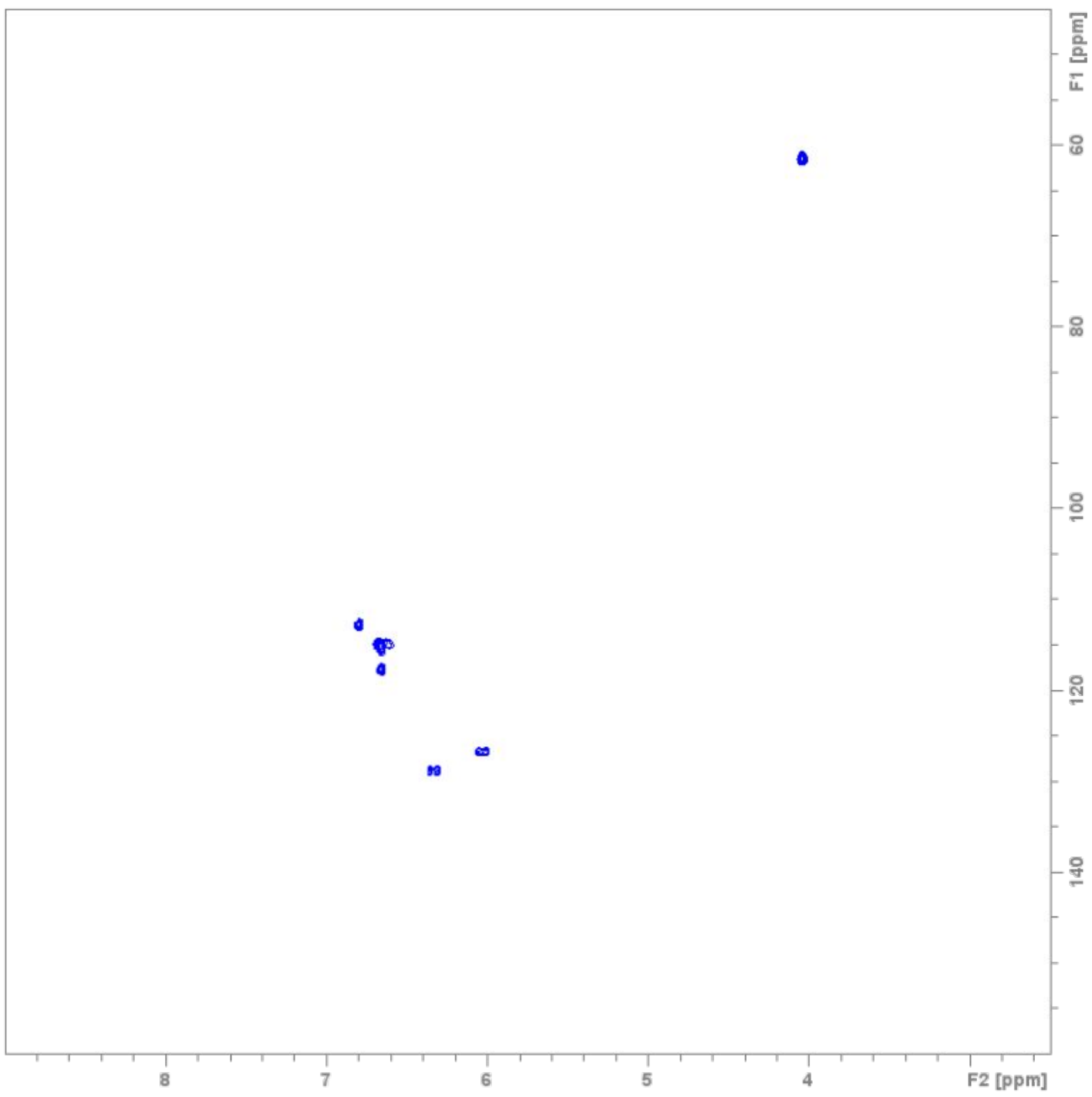

Figure S9. Caffeoyl alcohol. ^{1}H-^{13}C HSQC spectrum in DMSO-d_6.

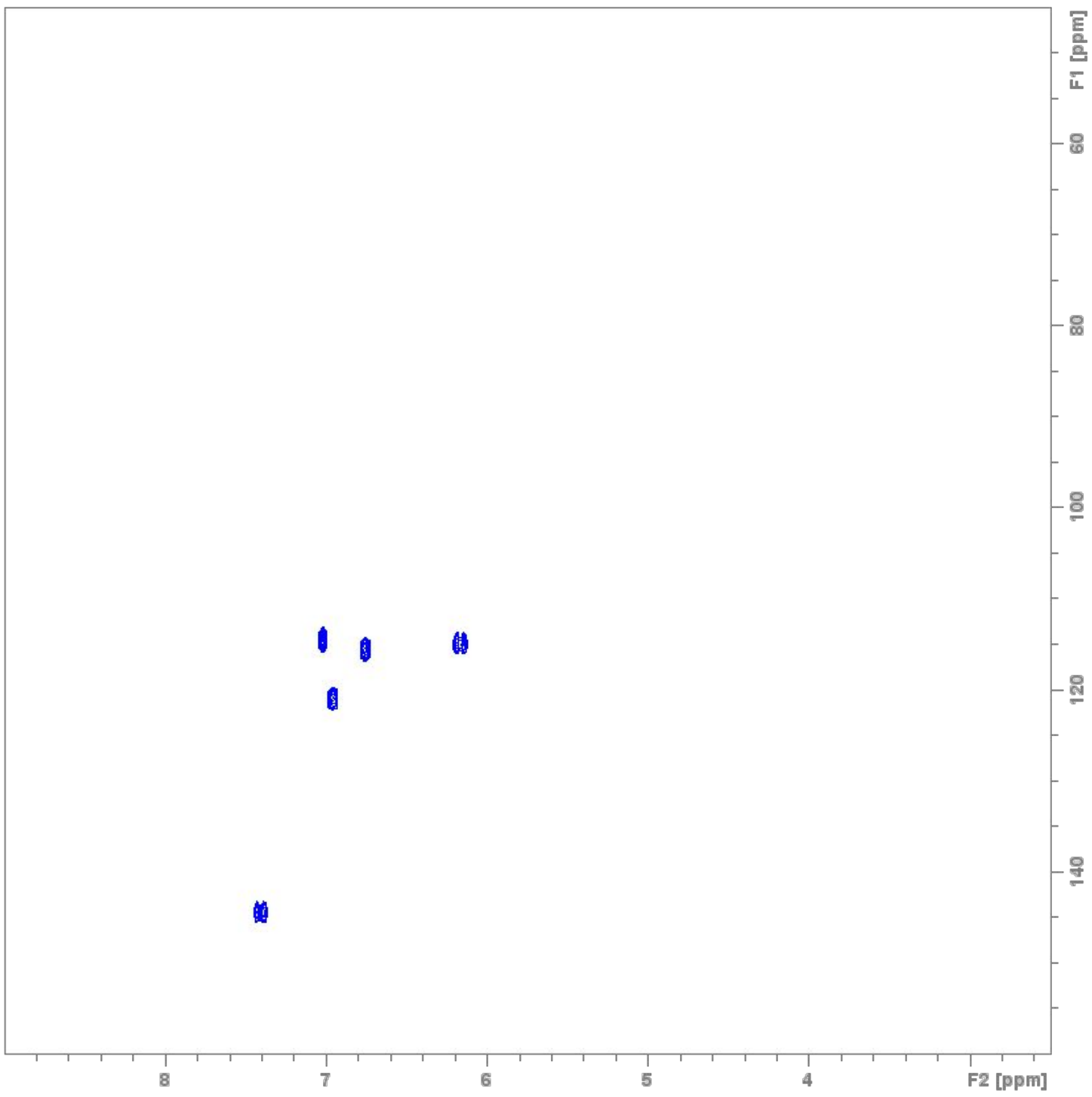

Figure S10. Caffeic acid. ^{1}H-^{13}C HSQC spectrum in DMSO-d_6.

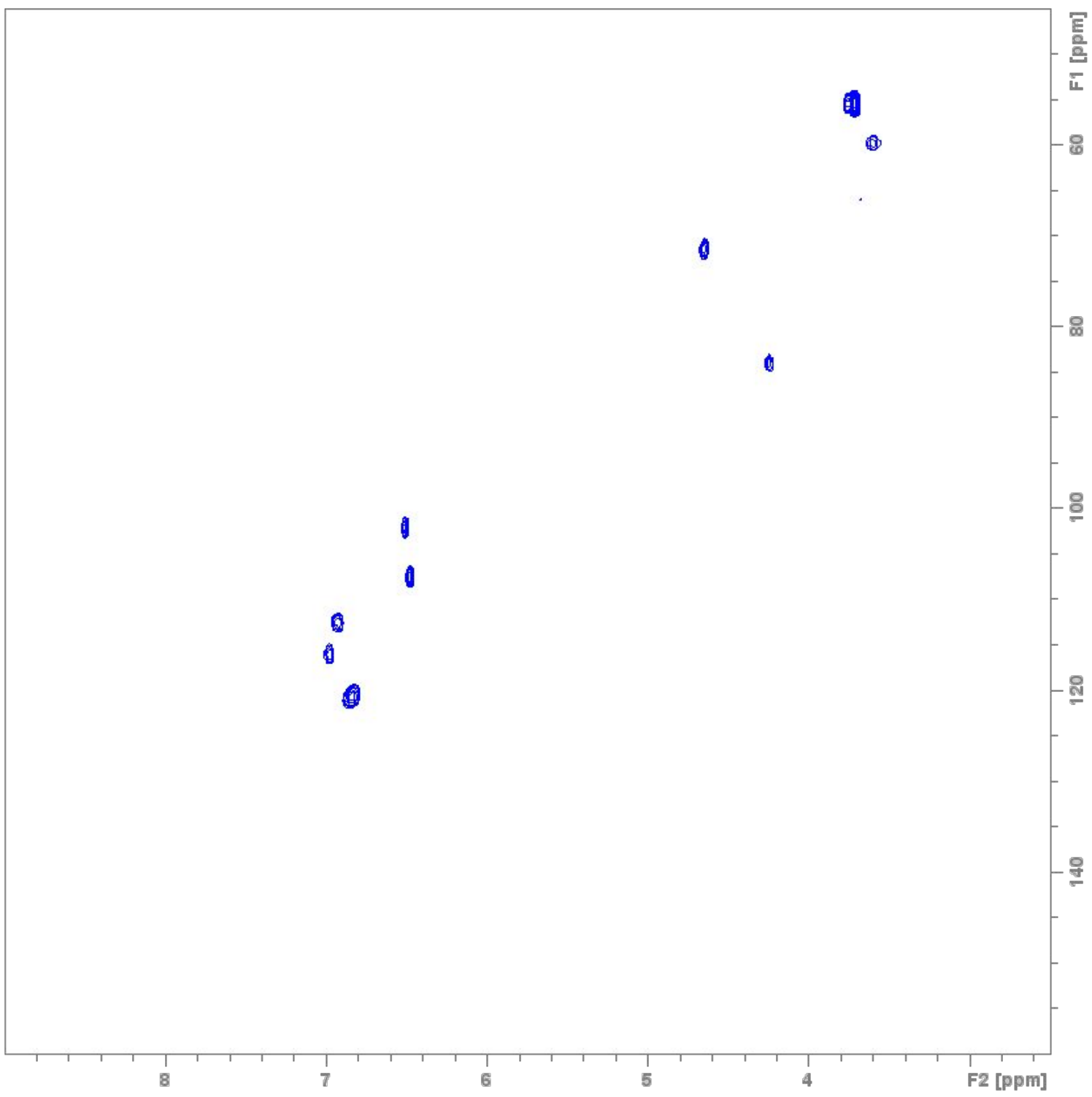

Figure S11. 5-methyl-catechyl-(β-O-4′). ^{1}H-^{13}C HSQC spectrum in DMSO-d_6.

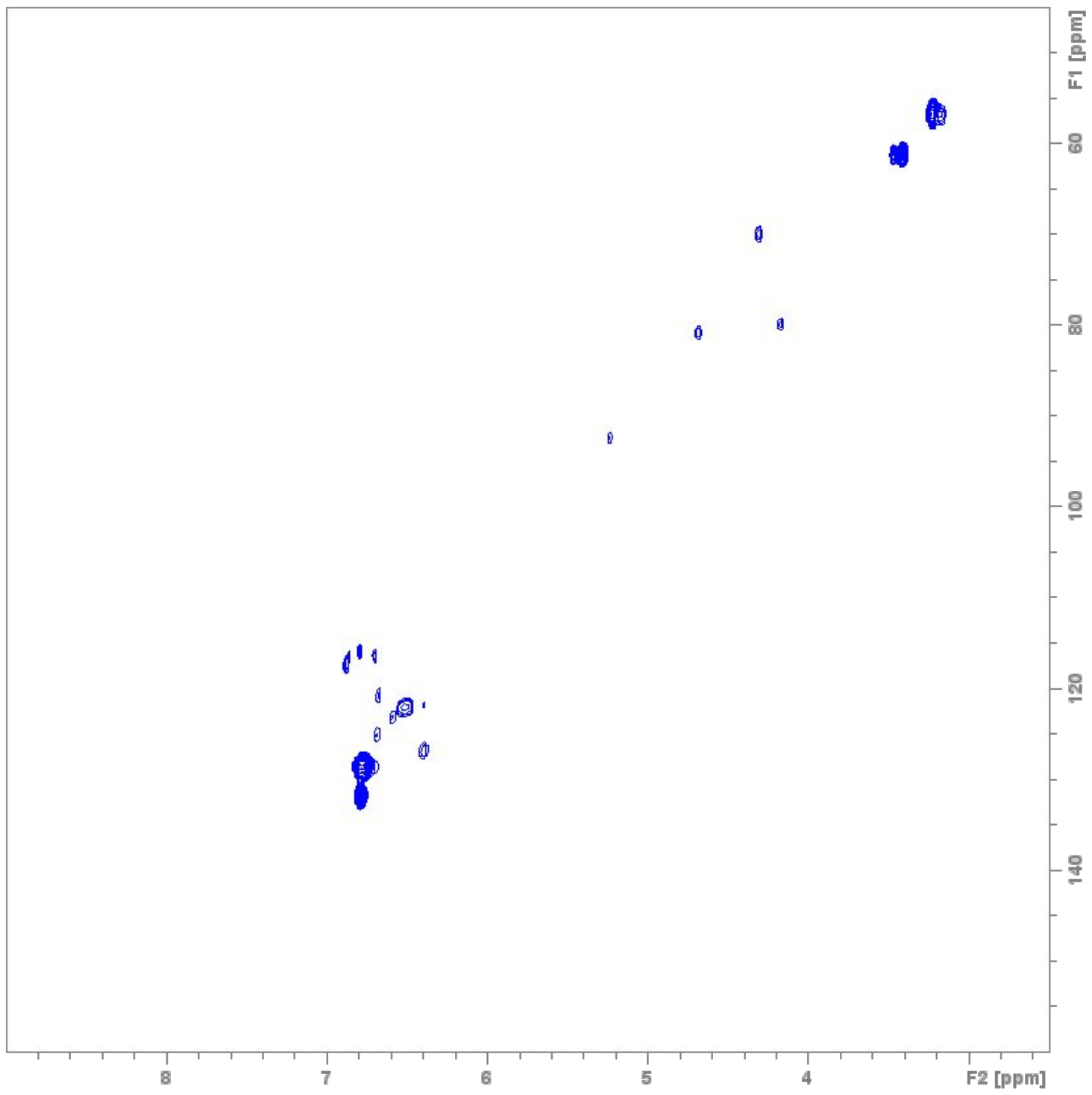

_**Figure S12**. Benzo-dioxane. ^{1}H-^{13}C HSQC spectrum in DMSO-d_6.

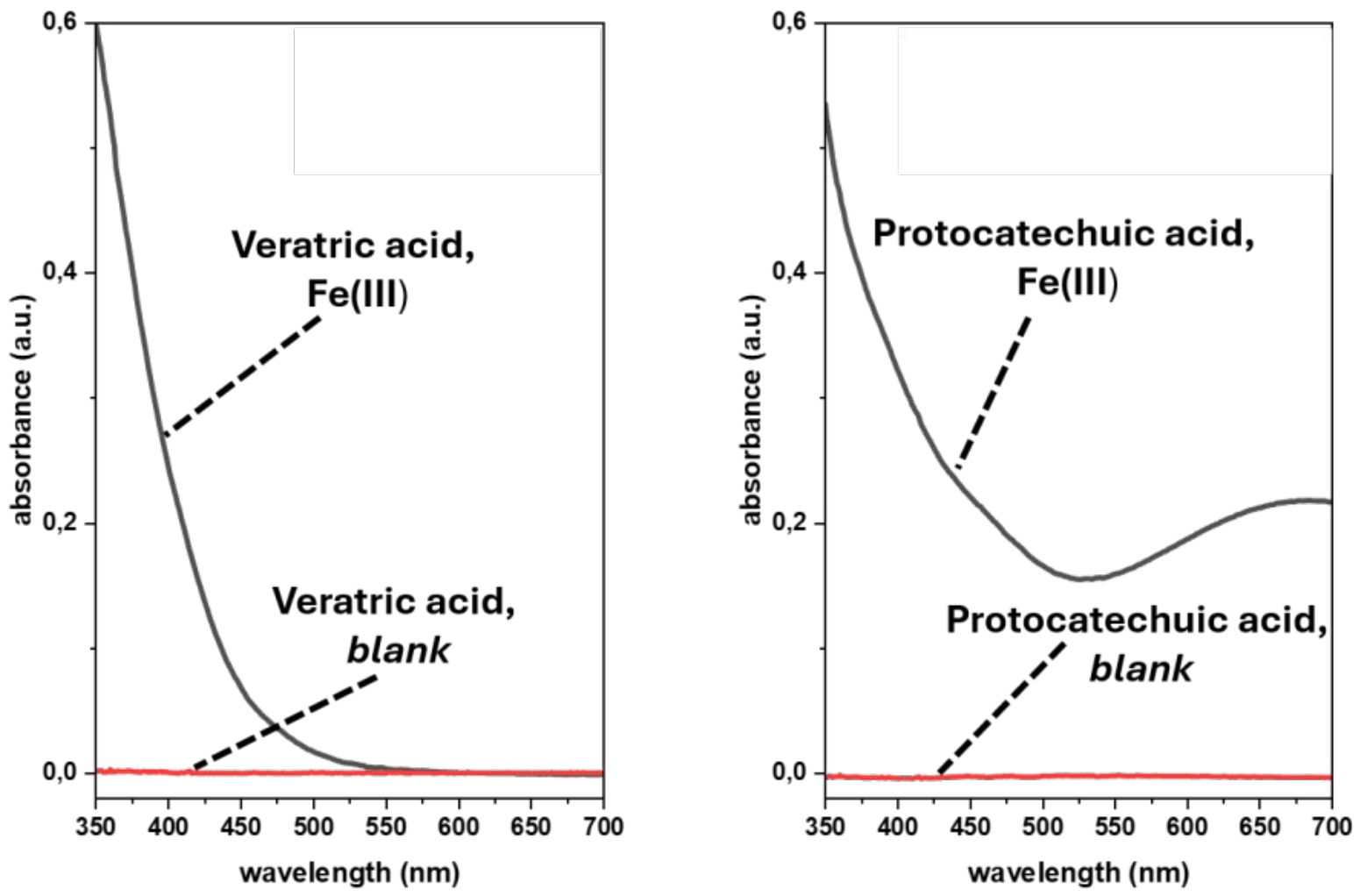

Figure S13. UV-vis spectra of veratric and protocatechuic acid in presence and absence of iron (III).

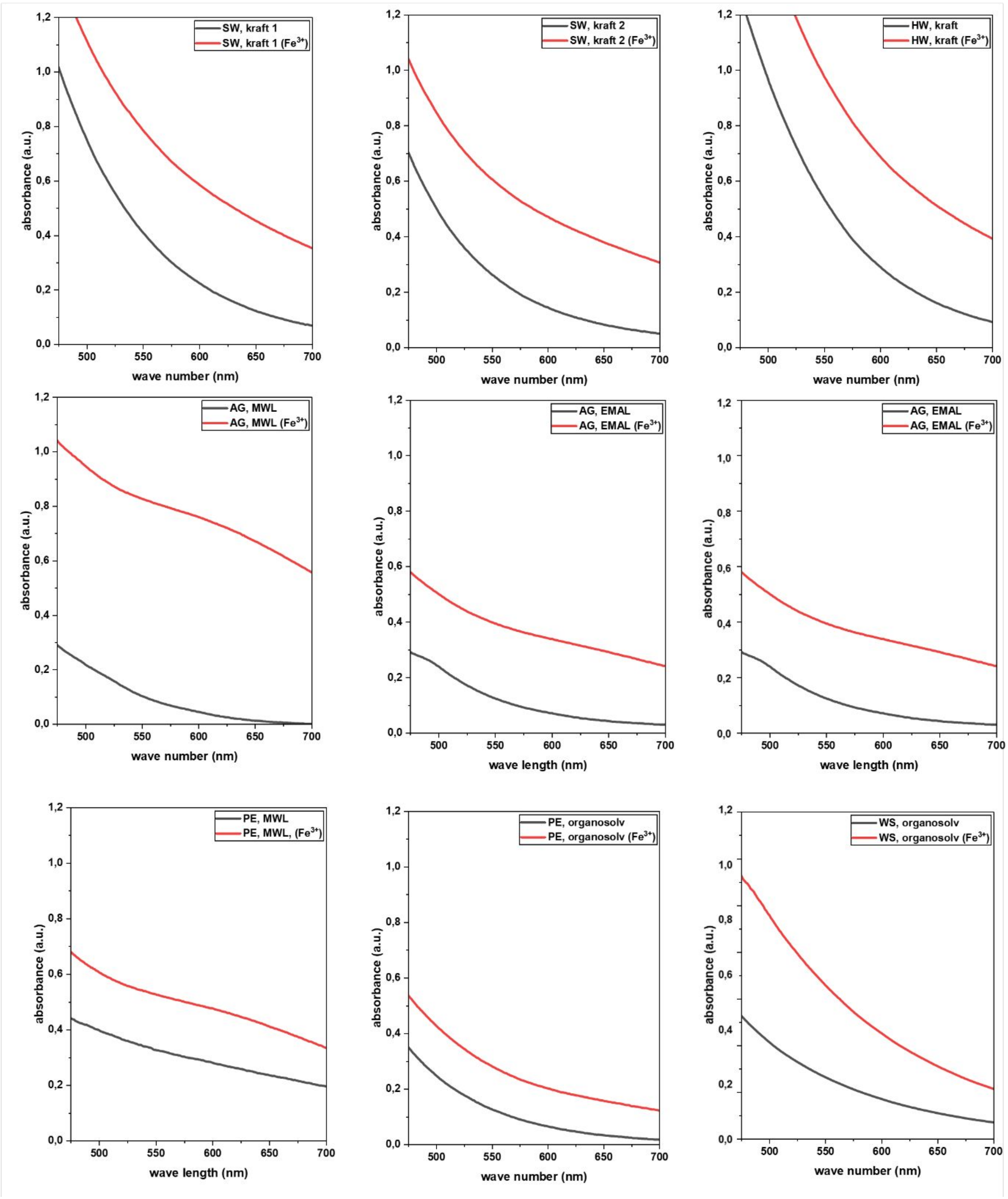

Figure S14. UV-vis spectra of investigated lignins in the presence and the absence of iron (III) chloride.

^{31}P NMR spectra of discussed lignins

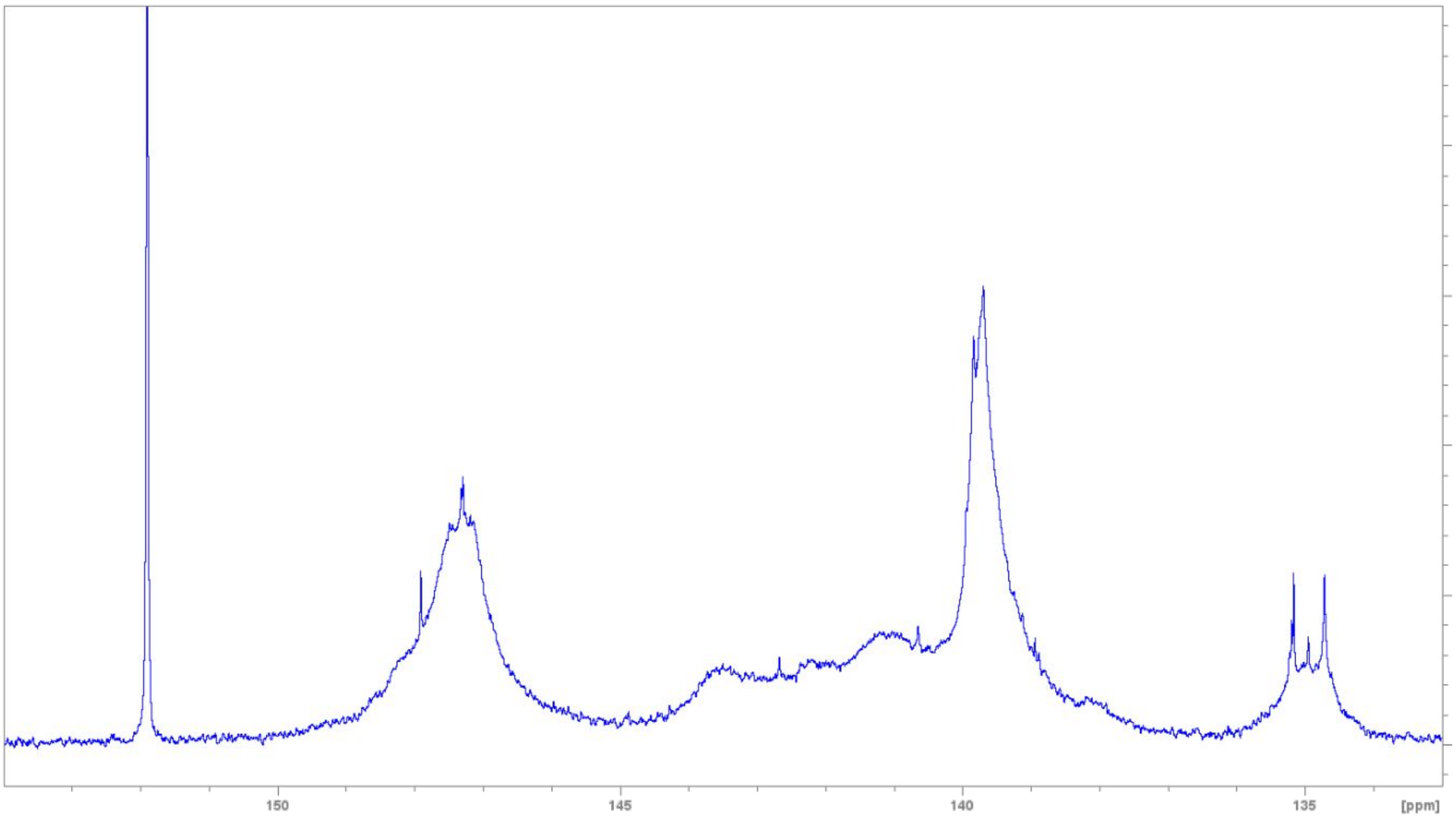

Figure S15. ^{31}P NMR spectrum of SW kraft, 1 in pyridine/$CDCl_3$.

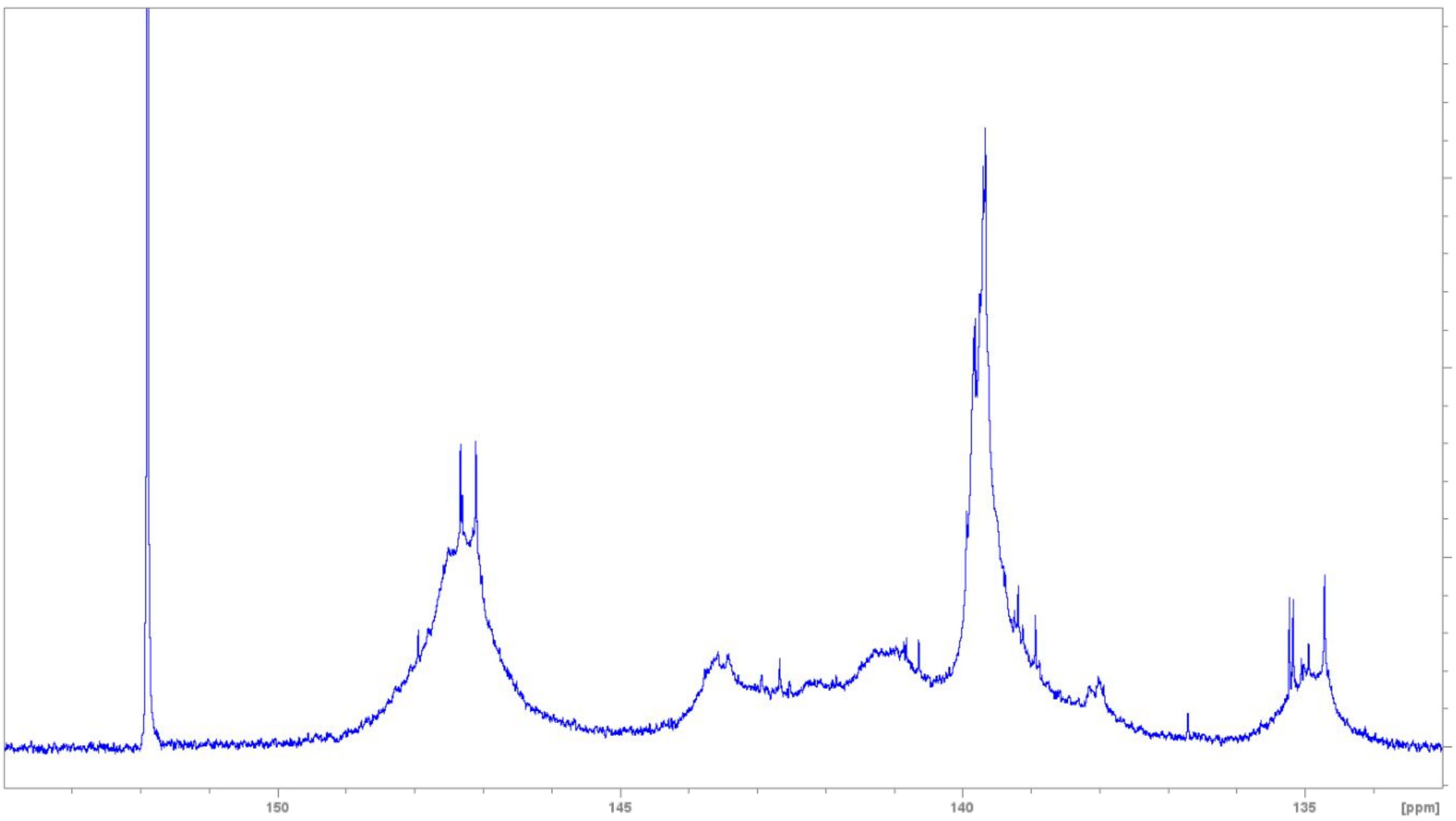

Figure S16. ^{31}P NMR spectrum of SW kraft, 2 in pyridine/$CDCl_3$.

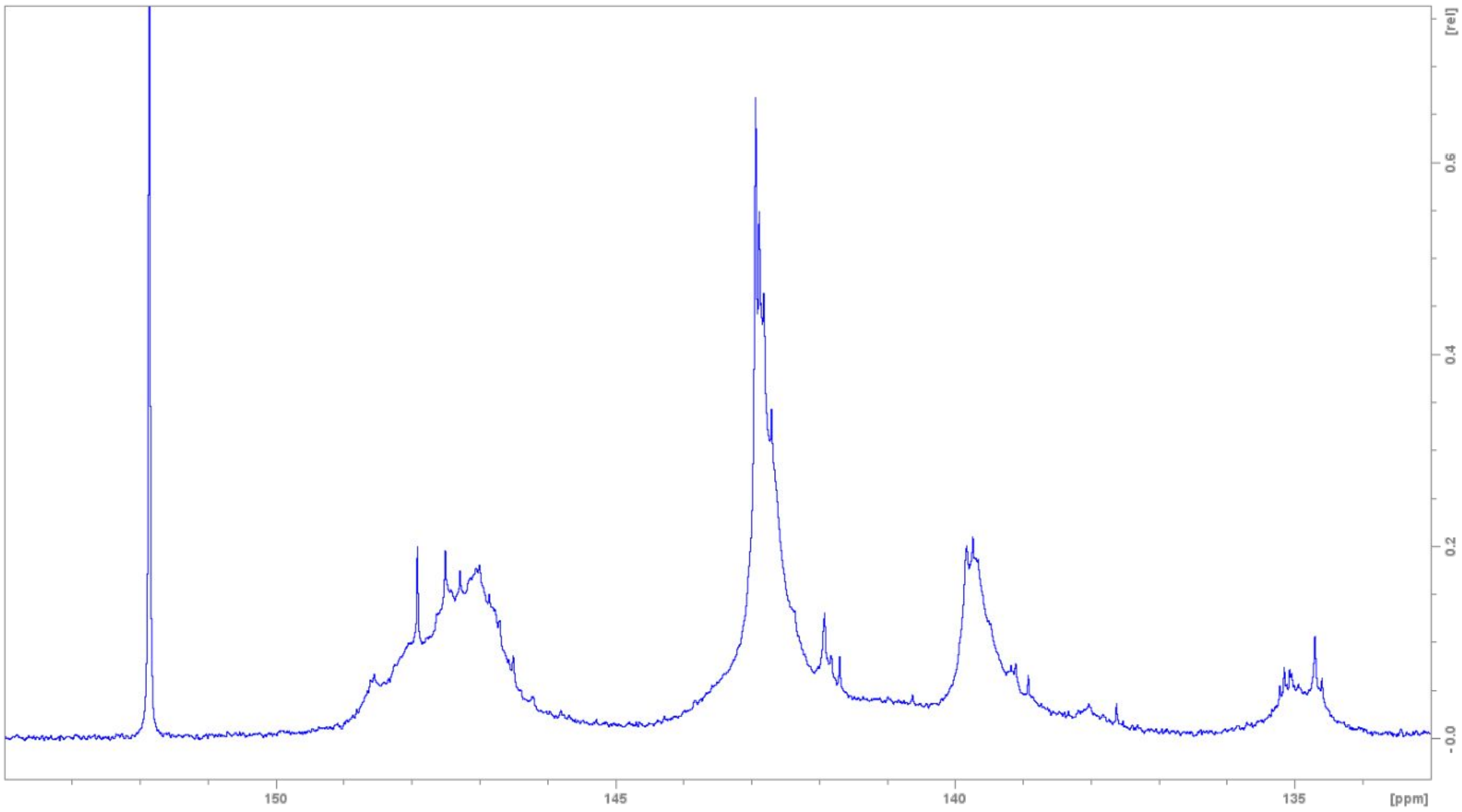

Figure S17. ^{31}P NMR spectrum of HW kraft in pyridine/$CDCl_3$.

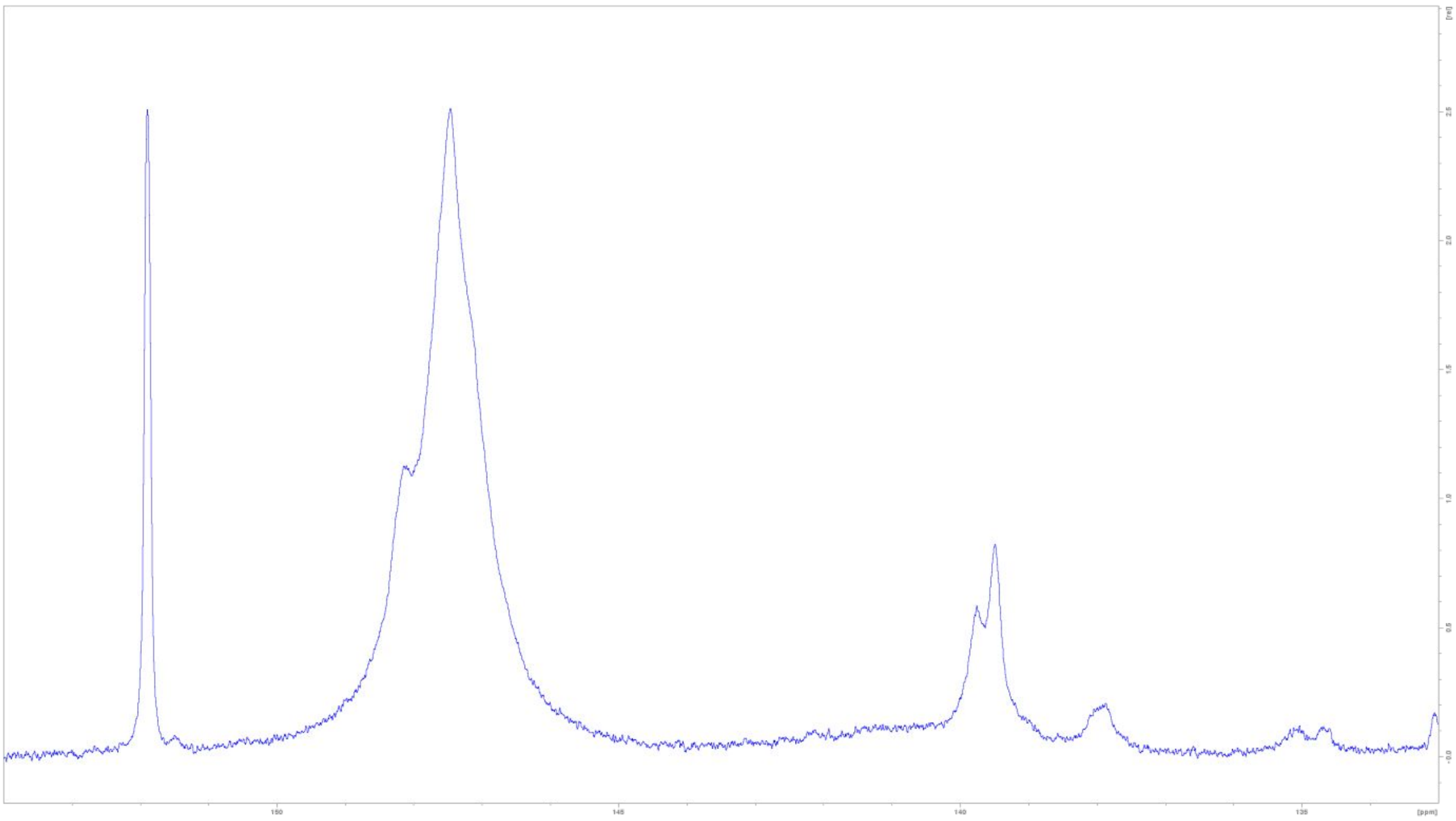

Figure S18. ^{31}P NMR spectrum of PE MWL in pyridine/$CDCl_3$.

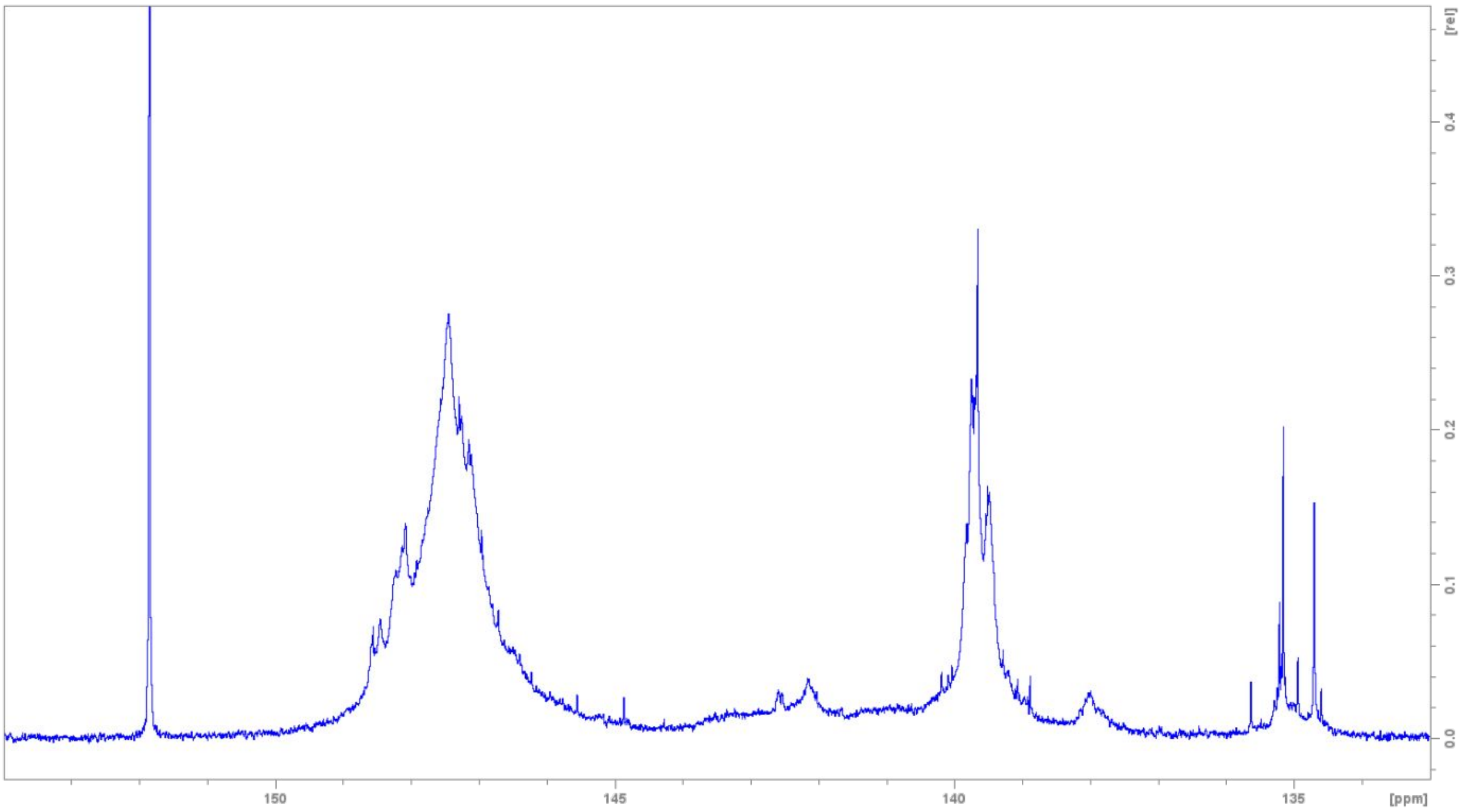

Figure S19. ^{31}P NMR spectrum of PE organosolv in pyridine/$CDCl_3$.

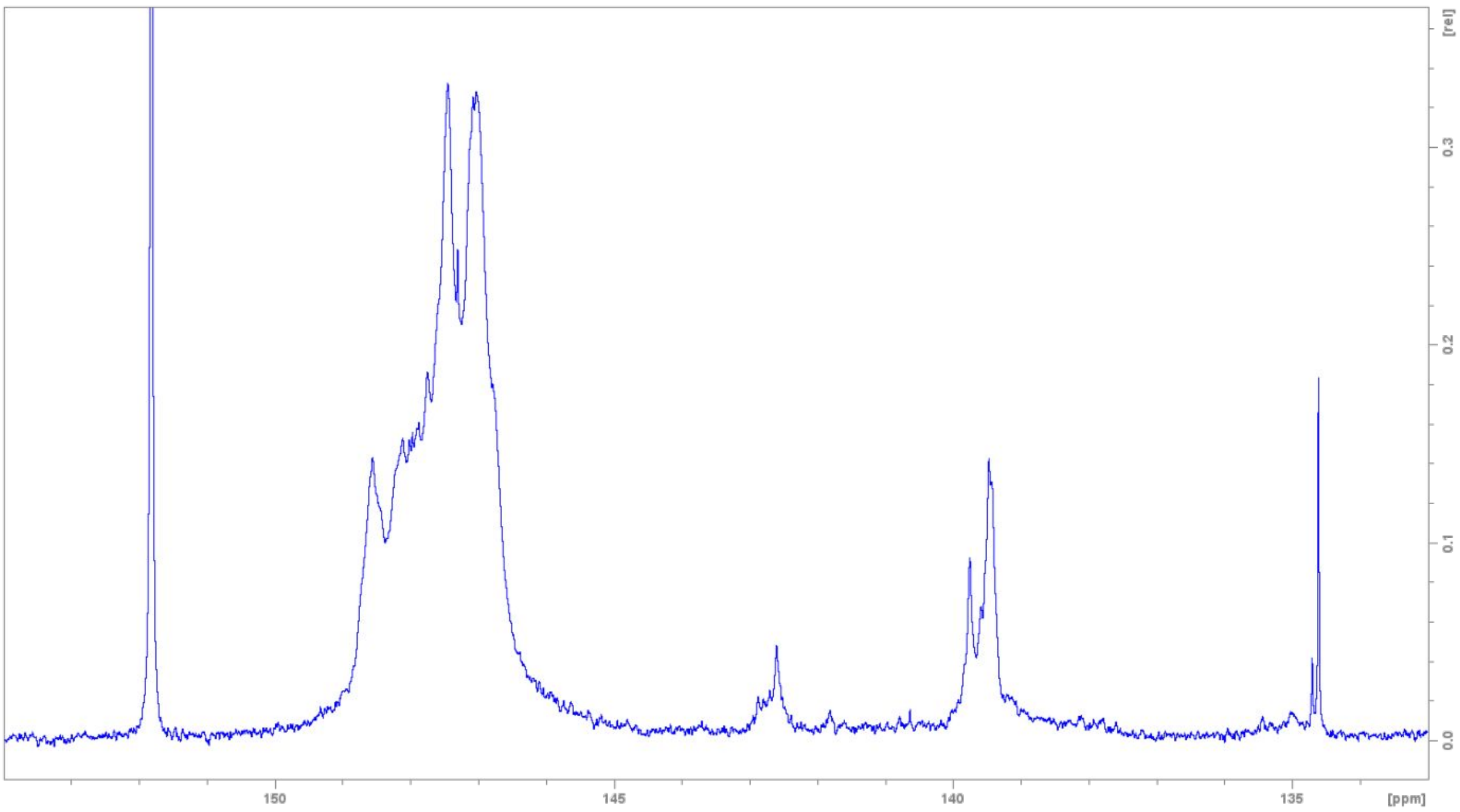

Figure S20. ^{31}P NMR spectrum of AG MWL in pyridine/$CDCl_3$.

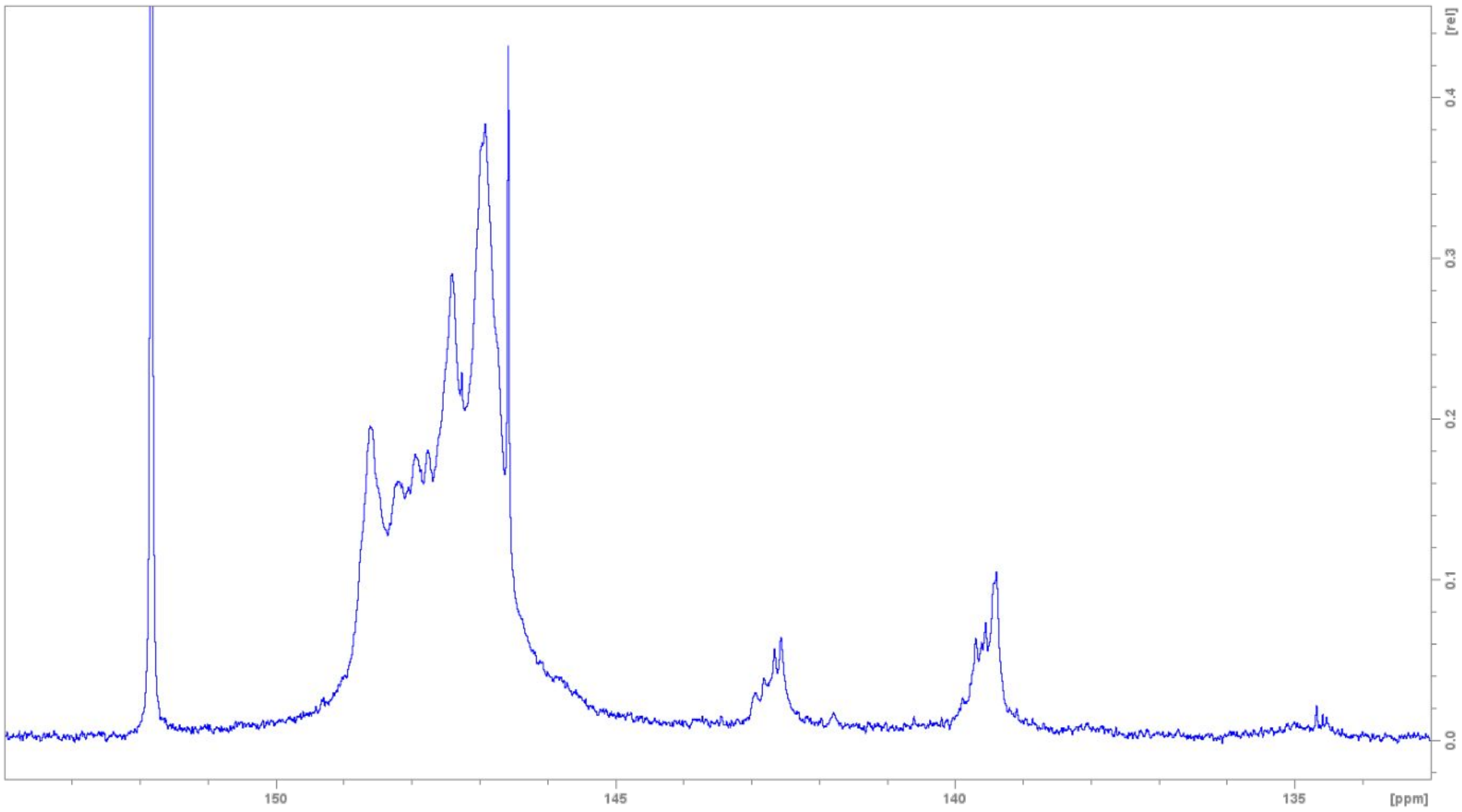

Figure S21. ^{31}P NMR spectrum of AG EMAL in pyridine/$CDCl_3$.

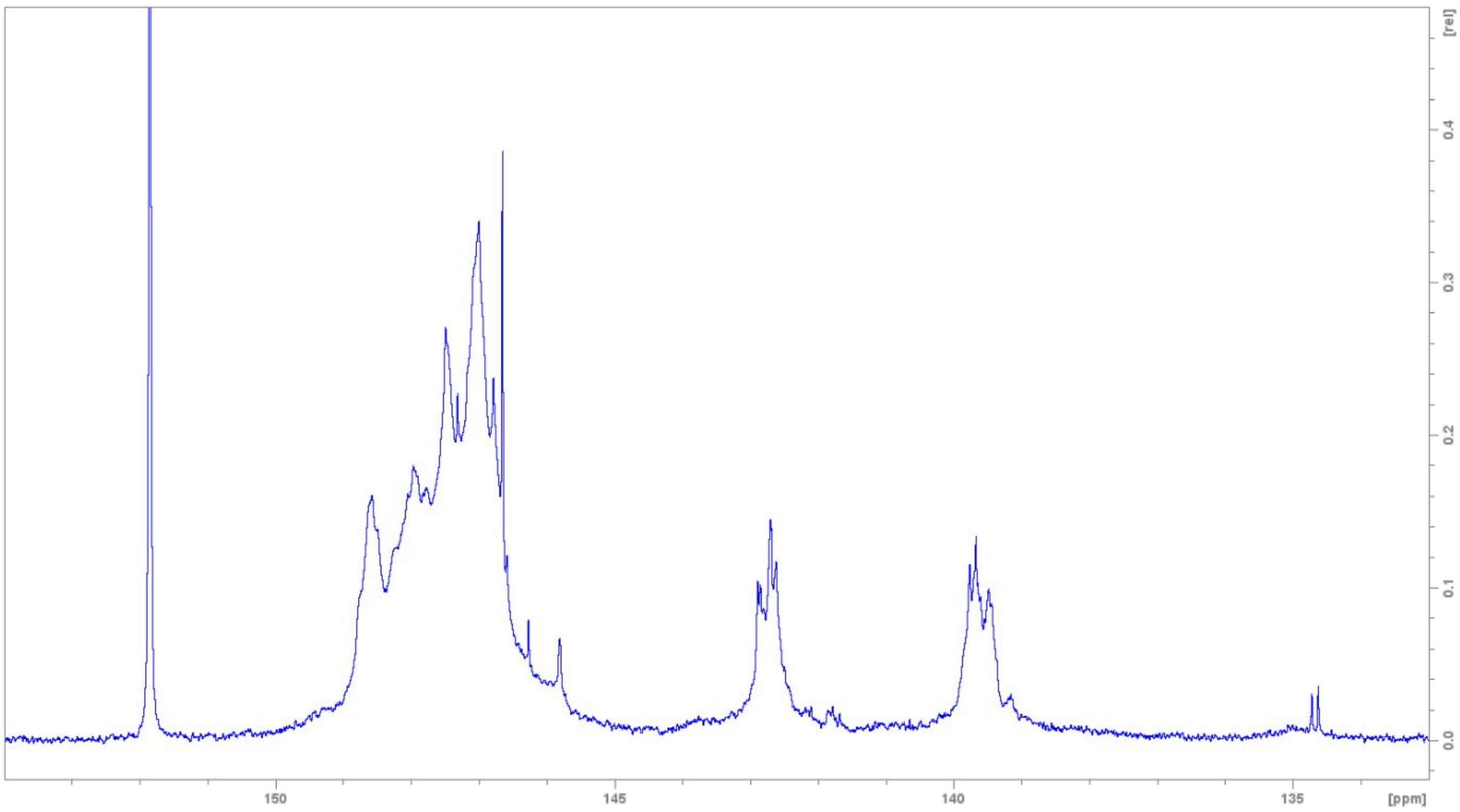

Figure S22. ^{31}P NMR spectrum of AG organosolv in pyridine/$CDCl_3$.

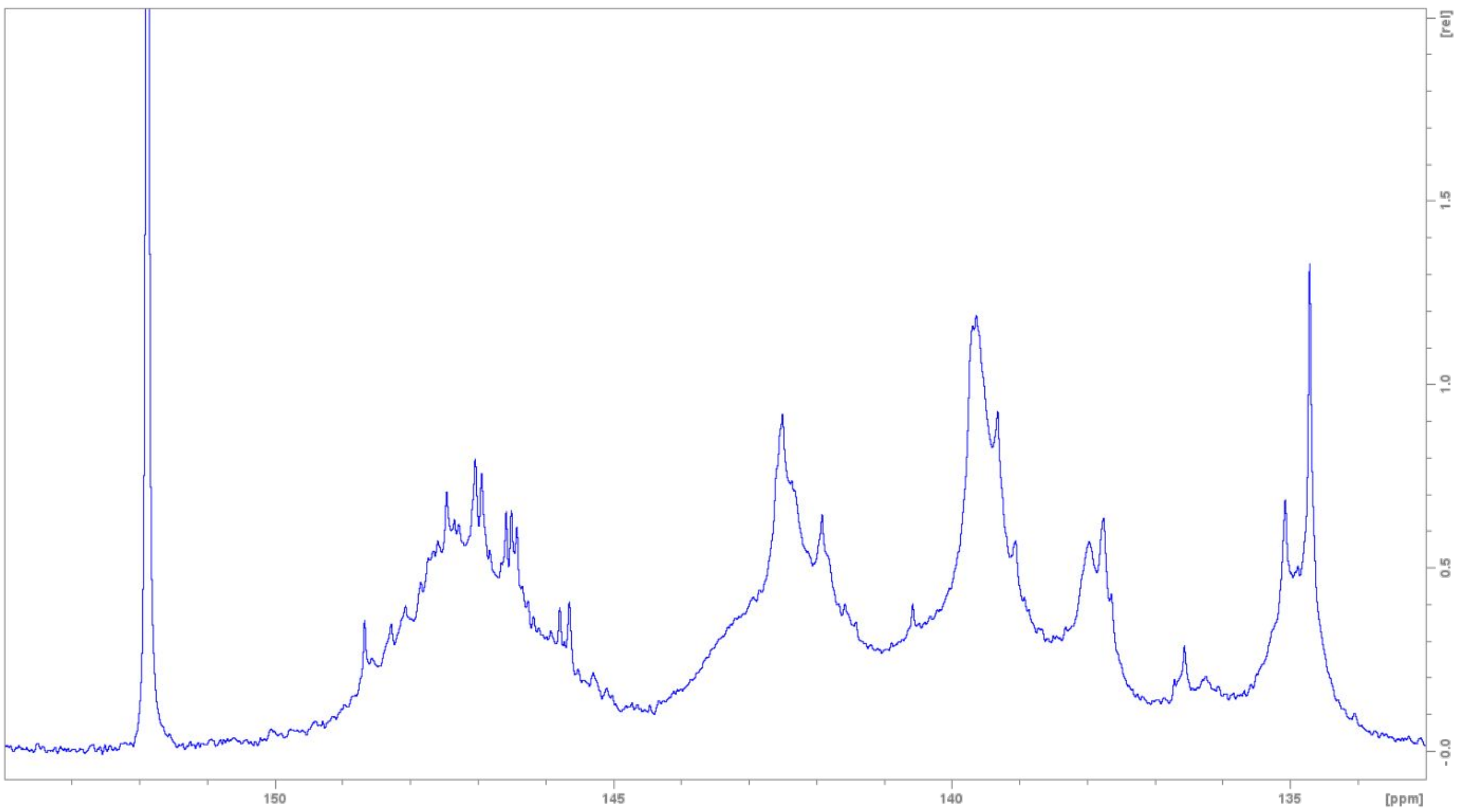

Figure S23. ^{31}P NMR spectrum of WS organosolv in pyridine/$CDCl_3$.

^{1}H-^{13}C HSQC spectra of discussed lignins

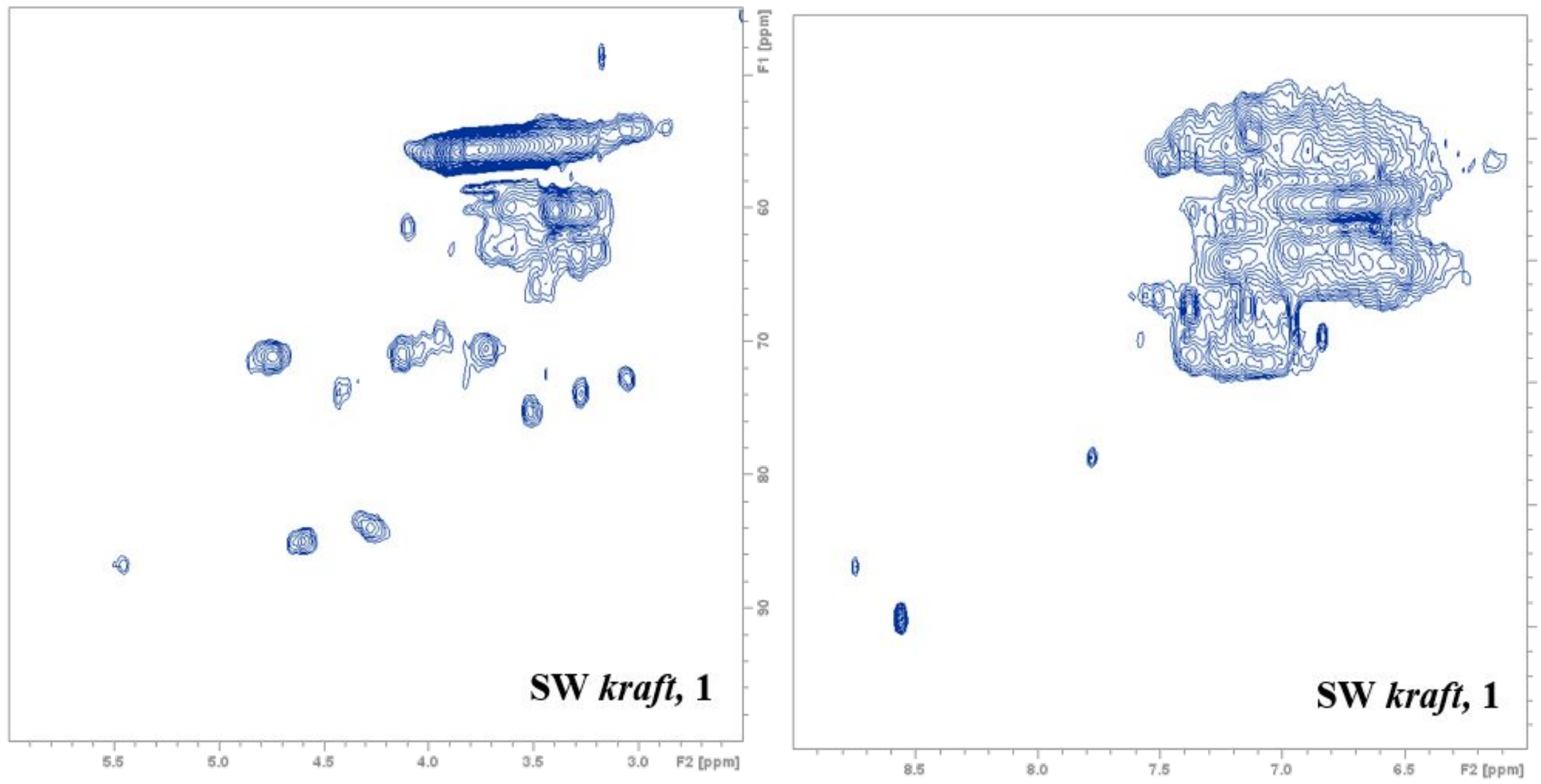

Figure S24. ^{1}H-^{13}C HSQC spectrum of SW kraft, 1 in DMSO-d_6.

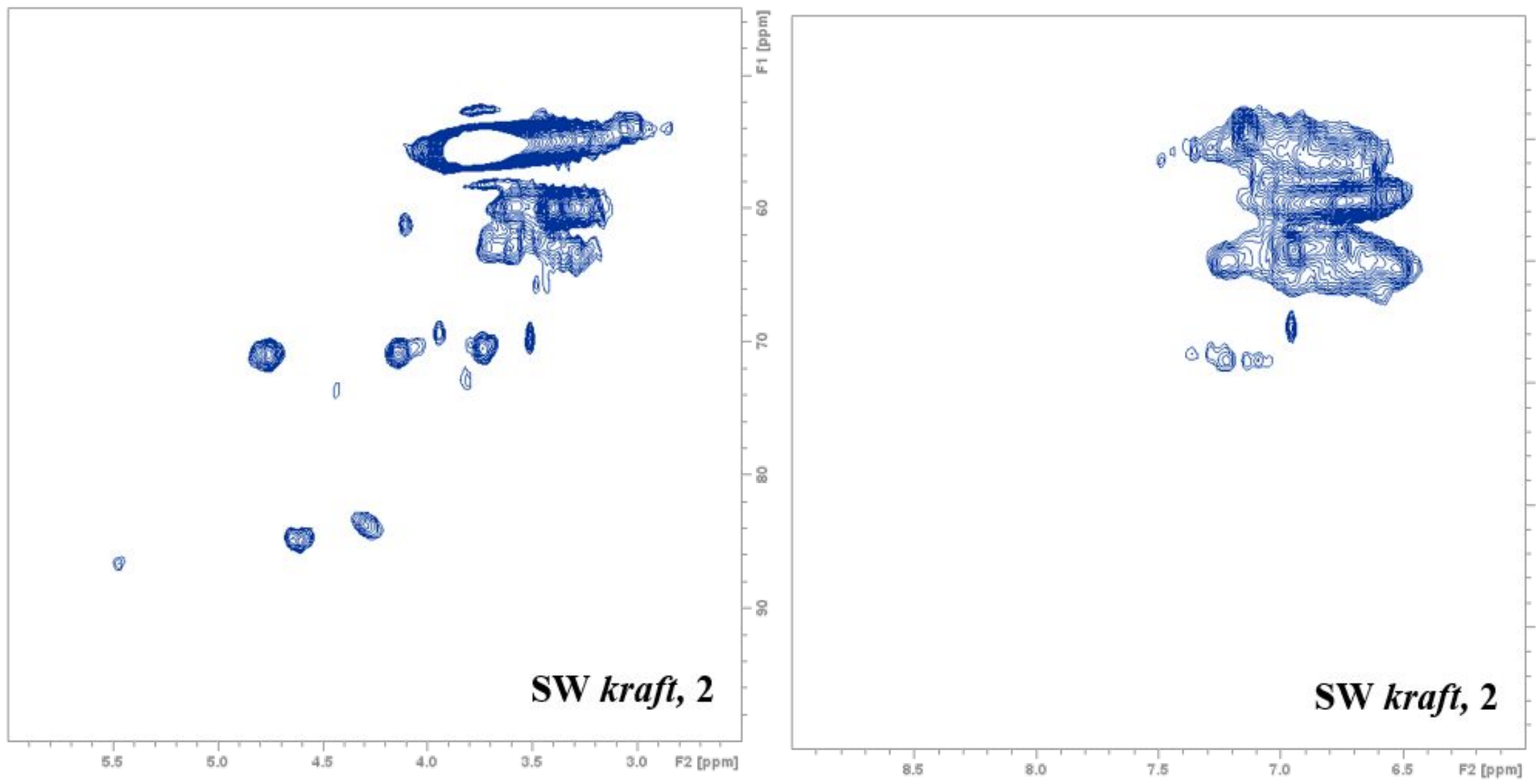

Figure S25. ^{1}H-^{13}C HSQC spectrum of SW kraft, 2 in DMSO-d_6.

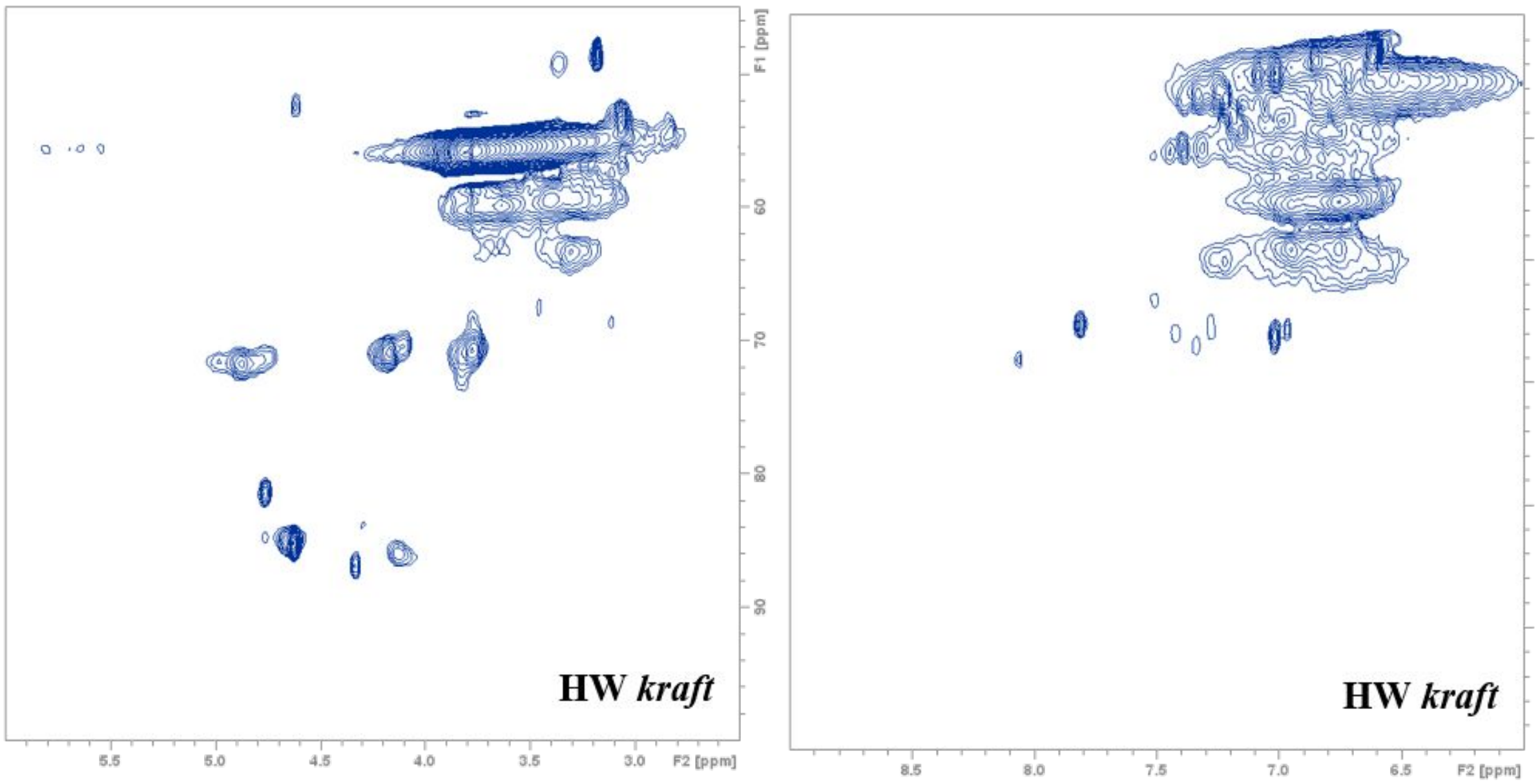

Figure S26. ^{1}H-^{13}C HSQC spectrum of HW kraft in DMSO-d_6.

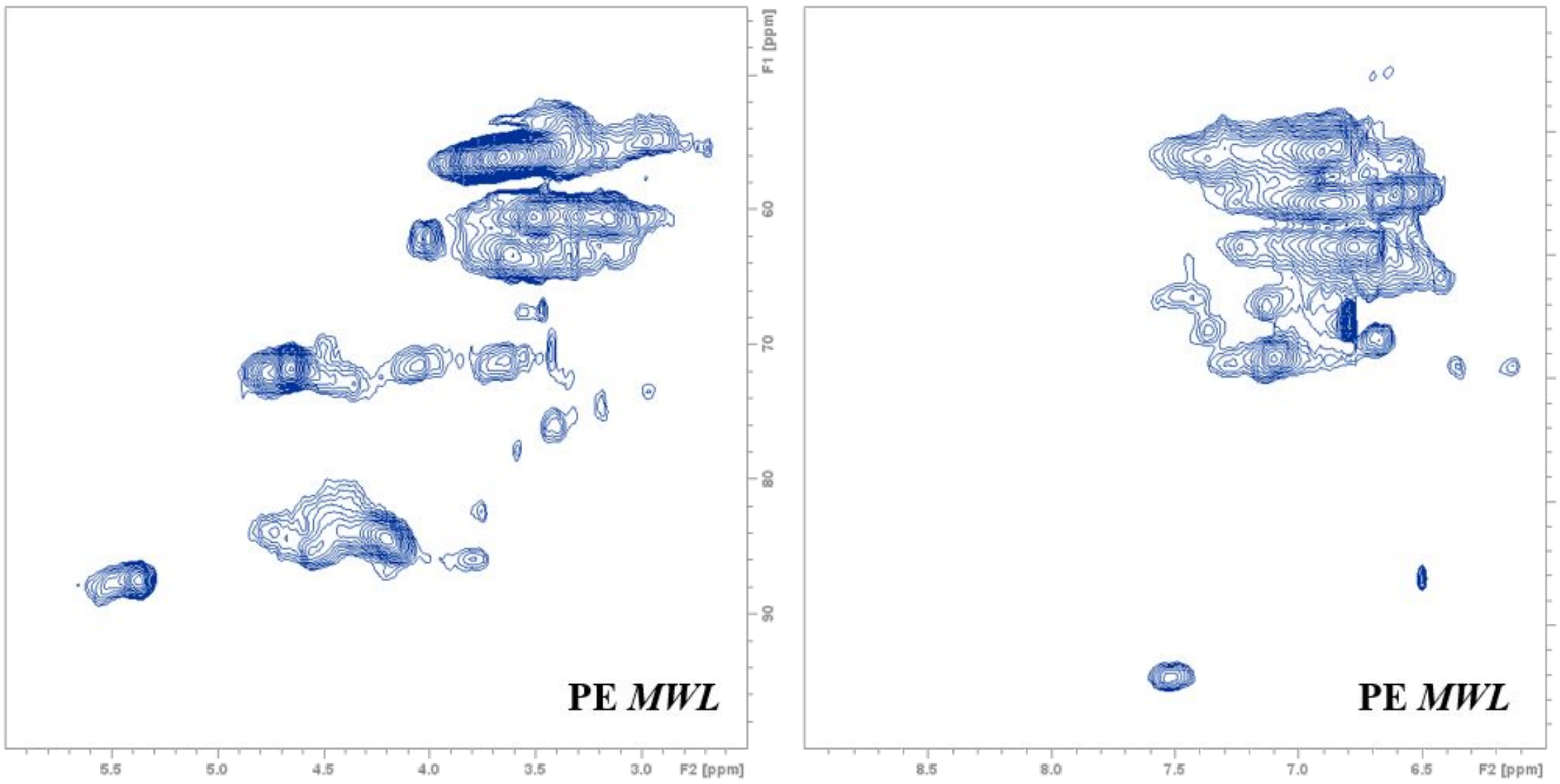

Figure S27. ^{1}H-^{13}C HSQC spectrum of PE MWL in DMSO-d_6.

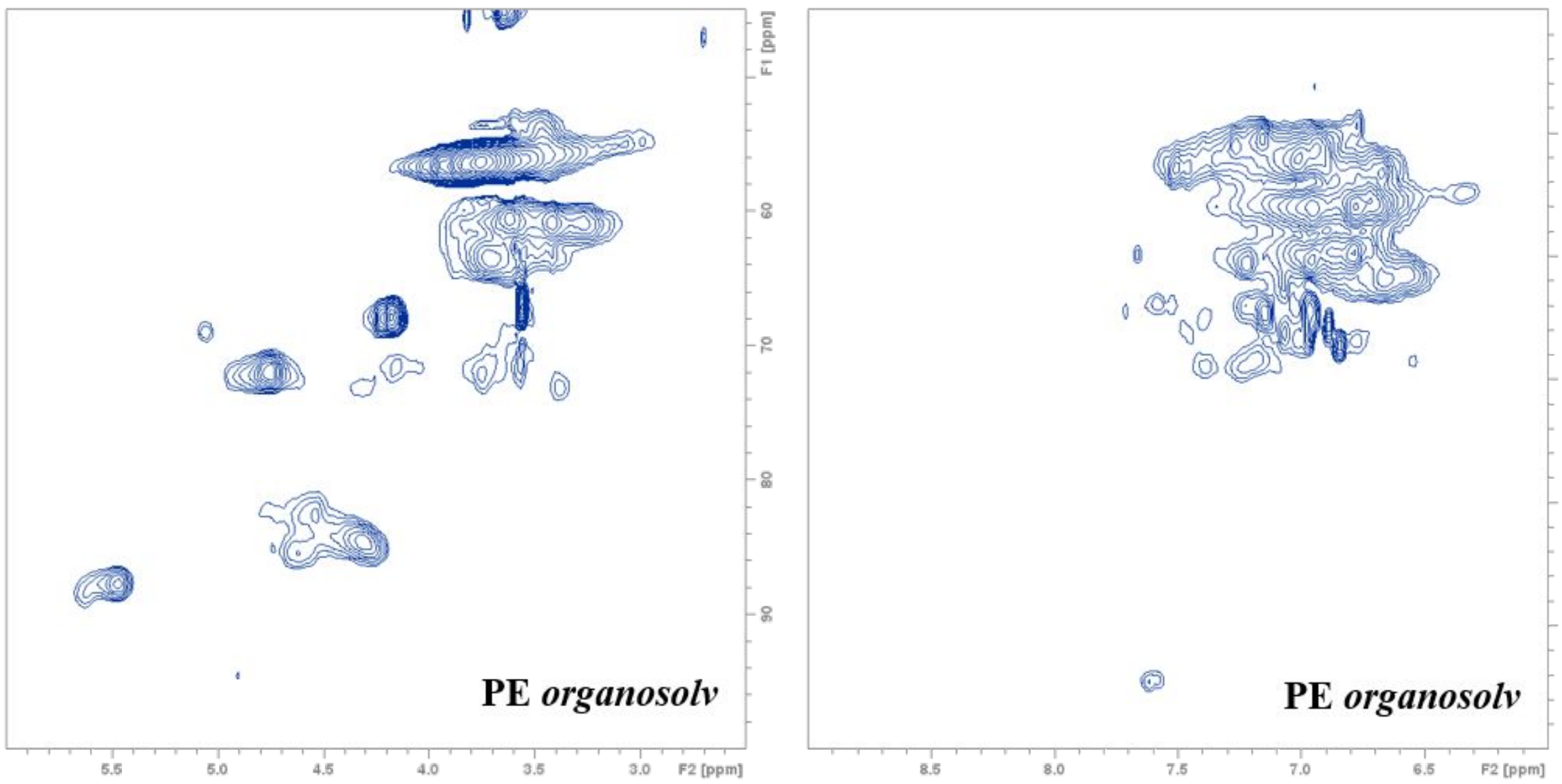

Figure S28. ^{1}H-^{13}C HSQC spectrum of PE organosolv in DMSO-d_6.

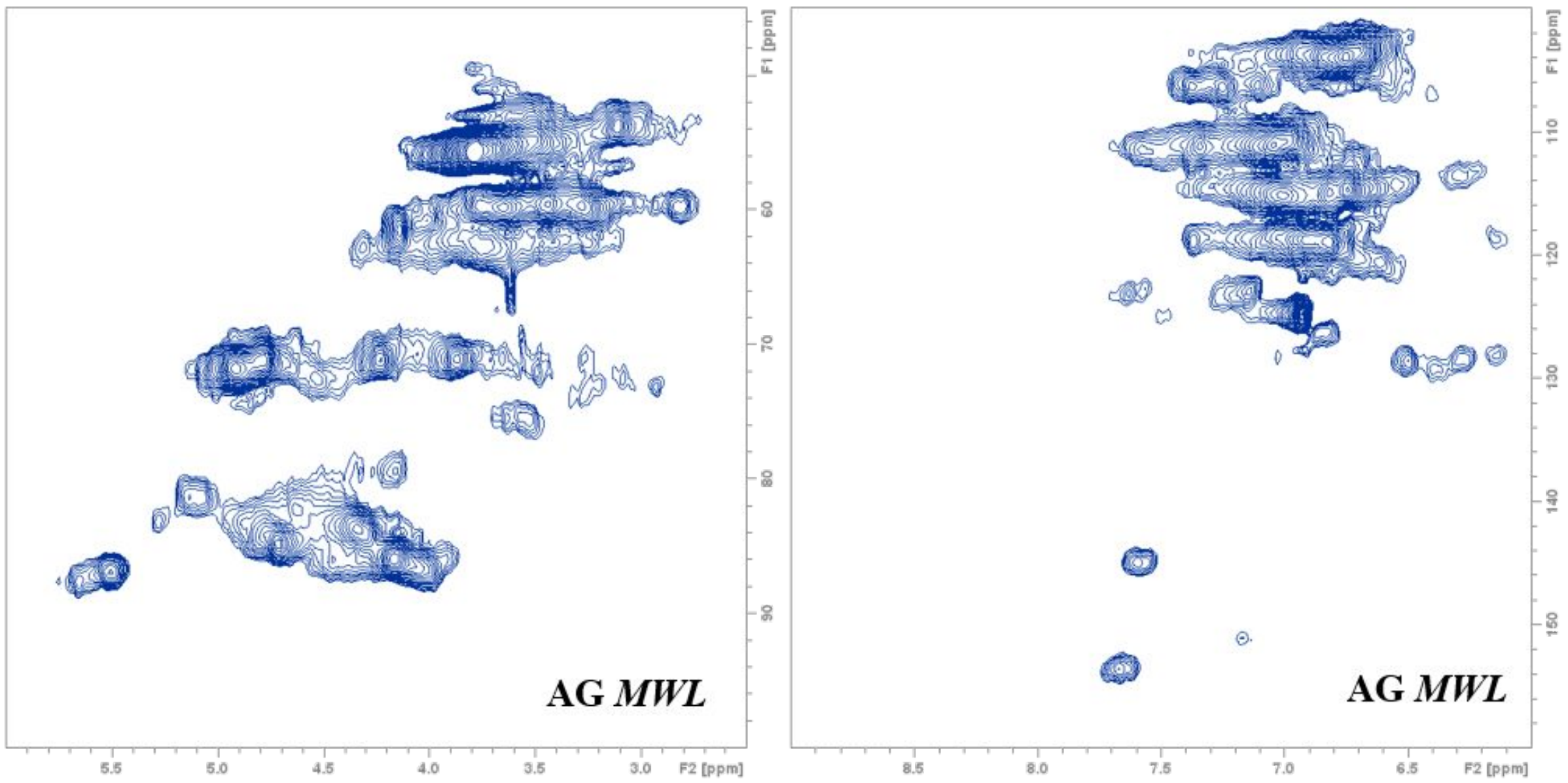

Figure S29. ^{1}H-^{13}C HSQC spectrum of AG MWL in DMSO-d_6.

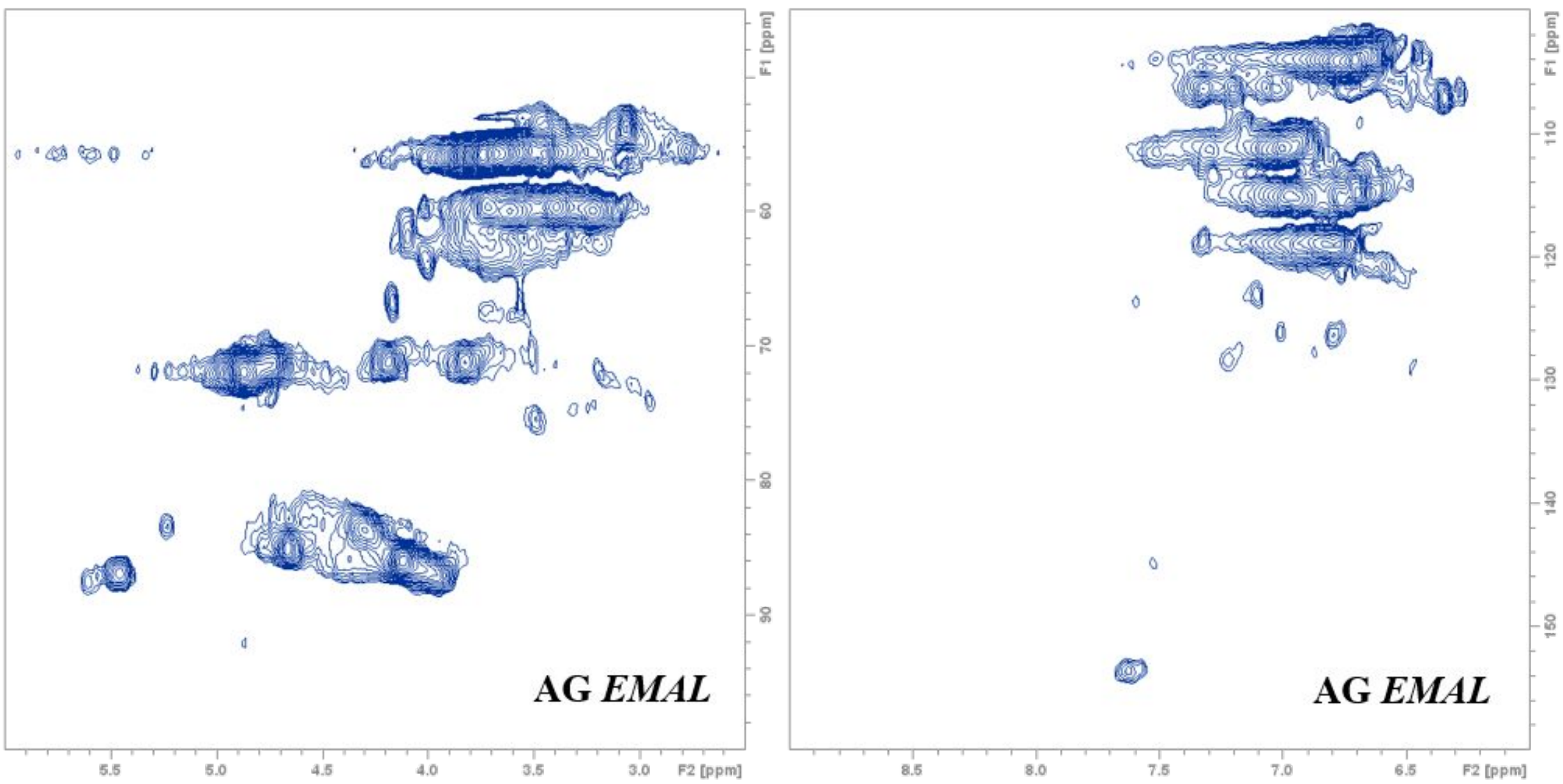

Figure S30. ^{1}H-^{13}C HSQC spectrum of AG EMAL in DMSO-d_6.

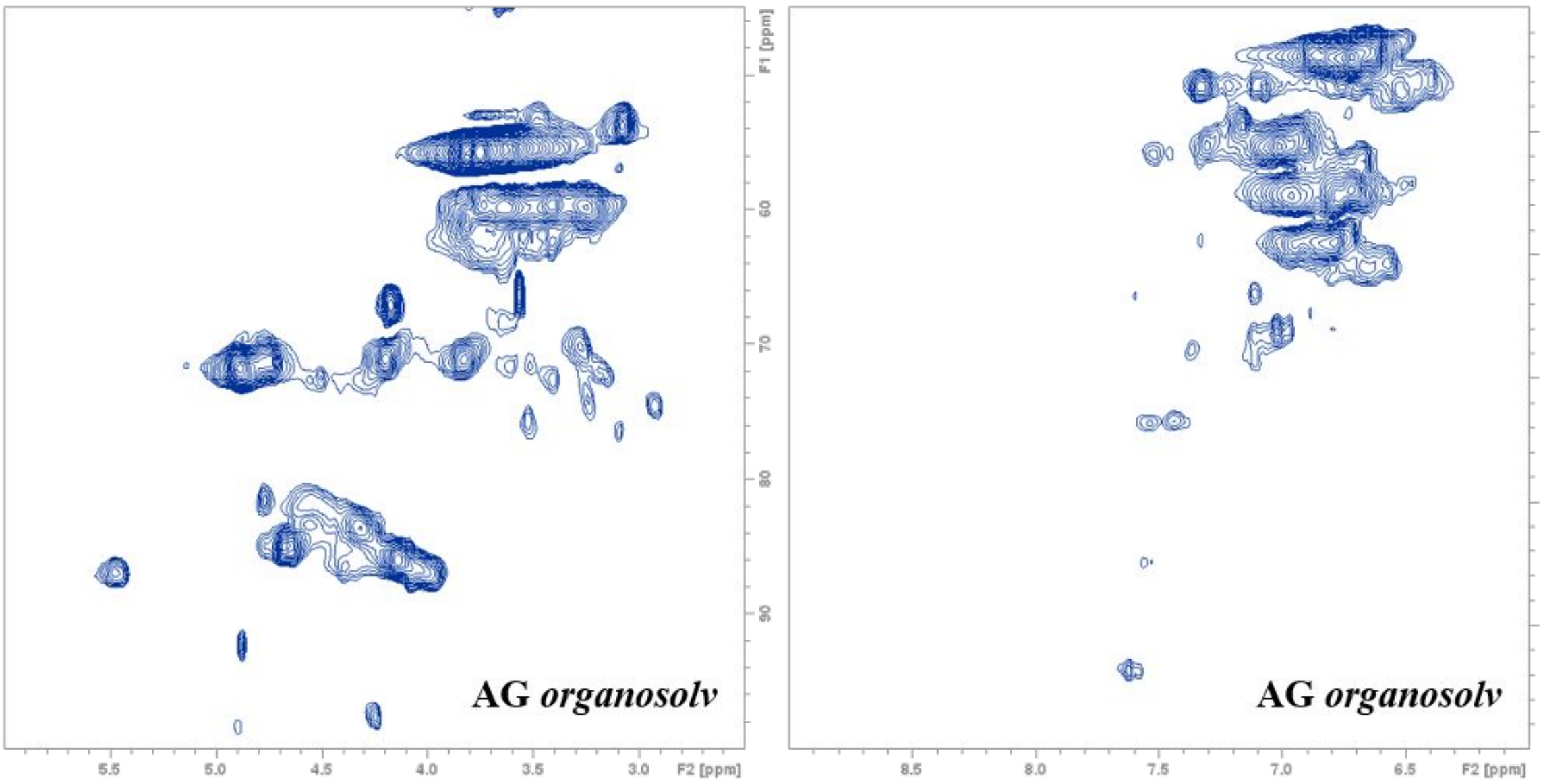

Figure S31. ^{1}H-^{13}C HSQC spectrum of AG organosolv in DMSO-d_6.

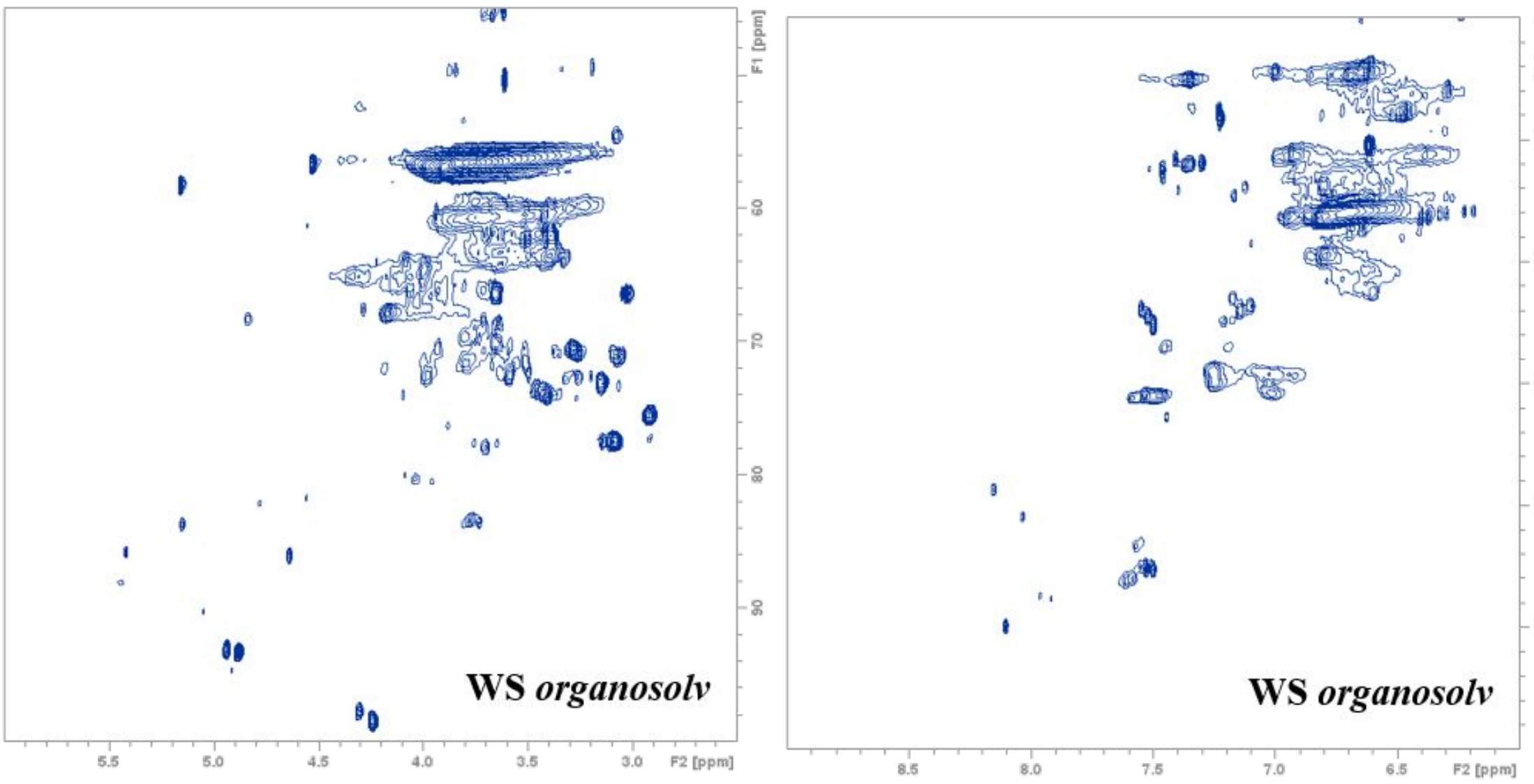

Figure S32. ^{1}H-^{13}C HSQC spectrum of WS organosolv in DMSO-d_6.

Table S1. Spiking tests using increasing amount of a 9.67 mM solution of *p*-methyl catechol on SKL solutions (SKL catechol content = 0.23 mmol g^{-1}, determined via chelometric/UV-vis method). Analyses were performed in triplicate.

**Lignin concentration** (mg mL^{-1})	**[catechol] (from SKL)** (mmol L^{-1})	**[catechol] (from spiked *p*-methyl-catechol)** (mmol L^{-1})	**Theoretical [catechol]** (mmol L^{-1})	**[catechol] via chelometric/UV-vis (mmol L^{-1})**
5.0	1.14	0.08	1.22	1.33±0.01
5.3	1.23	0.06	1.29	1.35±0.01
5.5	1.27	0.10	1.37	1.41±0.01

Table S2. Catechyl-adjusted guaiacyl contents for different technical and analytical grade lignins. Absorbance maxima were determined at 570-572 nm. Results reported are the result of at least three independent measurements. The maximum experimental error in our ^{31}P NMR analyses was quantified as being 0.01-0.02 mmol g^{-1} aligning with standard protocols[4,5].

	**S/G [a]**	**Catechols by UV-vis (mmol g^{-1})**	**Apparent guaiacyl by ^{31}P NMR (mmol g^{-1})[b]**	**Catechyl-adjusted guaiacyl content (mmol g^{-1})[c]**	**Catechyl-adjusted guaiacyl content based on S/G (mmol g^{-1})[d]**
**SW kraft, 1[e]**	-	0.24 ± 0.03	1.95 ± 0.02	1.47 ± 0.04	-
**SW kraft, 2[f]**	-	0.23 ± 0.02	1.71 ± 0.02	1.25 ± 0.03	-
**HW kraft**	1.83	0.27 ± 0.03	1.31 ± 0.02	1.04 ± 0.04	1.12 ± 0.03
**PE MWL**	-	0.08 ± 0.01	0.92 ± 0.02	0.76 ± 0.03	-
**PE organosolv**	-	0.09 ± 0.01	1.52 ± 0.02	1.34 ± 0.03	-
**AG MWL**	0.79	0.07 ± 0.01	0.43 ± 0.02	0.36 ± 0.02	0.35 ± 0.02
**AG EMAL**	1.21	0.10 ± 0.02	0.64 ± 0.02	0.54 ± 0.03	0.55 ± 0.03
**AG organosolv**	1.65	0.13 ± 0.02	0.59 ± 0.02	0.46 ± 0.03	0.49 ± 0.03
**WS organosolv**	0.69	0.06 ± 0.01	1.05 ± 0.02	0.99 ± 0.02	0.98 ± 0.02

[a] Determined from the ^{1}H-^{13}C HSQC spectrum of the sample from the areas of the peaks pertaining to guaiacyl (G_2), α-oxidised guaiacyl (G_2'), syringyl ($S_{2,6}$), and α-oxidised syringyl ($S_{2,6}'$), rings;
[b] Determined based on the integration of the traditionally assigned peak of guaiacyl units comprised between 140.2-139.0 ppm in TMDP-labelled lignins;.
[c] Calculated using Equations 1, 2, and 3 in the main text;
[d] Calculated using Equation 4 in the main text;
[e] Obtained from UPM (Finland);
[f] FP Innovations (Canada).

Table S3. Catechyl-adjusted content of phenolic hydroxyl (mono-hydroxyl phenols content) for different technical and analytical grade lignins. The maximum experimental error in our ^{31}P NMR analyses was quantified as being 0.01-0.02 mmol g⁻¹ aligning with standard protocols[4,5].

	**Catechols by UV-vis (mmol g⁻¹)**	**Phenols content by ^{31}P NMR (mmol g⁻¹)**	**Catechols-adjusted phenol content (mmol g⁻¹)[a]**
**SW kraft, 1[b]**	0.24 ± 0.03	3.61 ± 0.02	3.13 ± 0.04
**SW kraft, 2c**	0.23 ± 0.02	3.30 ± 0.02	2.84 ± 0.03
**HW kraft**	0.27 ± 0.03	4.48 ± 0.02	3.94 ± 0.04
**PE MWL**	0.08 ± 0.01	2.46 ± 0.02	2.30 ± 0.03
**PE organosolv**	0.09 ± 0.01	2.17 ± 0.02	1.99 ± 0.03
**AG MWL**	0.07 ± 0.01	0.64 ± 0.02	0.50 ± 0.02
**AG EMAL**	0.10 ± 0.02	1.07 ± 0.02	0.87 ± 0.03
**AG organosolv**	0.13 ± 0.02	1.06 ± 0.02	0.80 ± 0.03
**WS organosolv**	0.06 ± 0.01	2.67 ± 0.02	2.55 ± 0.02

[a] Calculated using Equation 5 in the main text;
[b] Obtained from UPM (Finland);
[c] FP Innovations (Canada).

References

(1) Pajer, N.; Danelon, U.; Crestini, C. NMR Spectroscopy: An Invaluable Tool to Identify Lignin Structural Modifications Induced by Oxidative Enzymes. In *Lignin-Degrading Enzymes*; Methods in Enzymology; 2025; Vol. 716.

(2) Pajer, N.; Crestini, C. Quantification of Carbonyl Groups in Lignin: 31P NMR after Reduction. *ACS Sustainable Chem. Eng.* **2025**, *13* (36), 15189–15200. https://doi.org/10.1021/acssuschemeng.5c06762.

(3) Granata, A.; Argyropoulos, D. S. 2-Chloro-4,4,5,5-Tetramethyl-1,3,2-Dioxaphospholane, a Reagent for the Accurate Determination of the Uncondensed and Condensed Phenolic Moieties in Lignins. *J. Agric. Food Chem.* **1995**, *43* (6), 1538–1544. https://doi.org/10.1021/jf00054a023.

(4) Meng, X.; Parikh, A.; Seemala, B.; Kumar, R.; Pu, Y.; Wyman, C. E.; Cai, C. M.; Ragauskas, A. J. Characterization of Fractional Cuts of Co-Solvent Enhanced Lignocellulosic Fractionation Lignin Isolated by Sequential Precipitation. *Bioresource Technology* **2019**, *272*, 202–208. https://doi.org/10.1016/j.biortech.2018.09.130.

(5) Argyropoulos, D. S.; Pajer, N.; Crestini, C. Quantitative 31P NMR Analysis of Lignins and Tannins. *JoVE (Journal of Visualized Experiments)* **2021**, No. 174, e62696. https://doi.org/10.3791/62696.
